# Circular RNA Profiling by Illumina Sequencing via Template-Dependent Multiple Displacement Amplification

**DOI:** 10.1155/2019/2756516

**Published:** 2019-01-28

**Authors:** Ashirbad Guria, Kavitha Velayudha Vimala Kumar, Nagesh Srikakulam, Anakha Krishnamma, Saibal Chanda, Satyam Sharma, Xiaofeng Fan, Gopal Pandi

**Affiliations:** ^1^Department of Plant Biotechnology, School of Biotechnology, Madurai Kamaraj University, Madurai 625021, Tamil Nadu, India; ^2^Division of Gastroenterology and Hepatology, Saint Louis University Liver Center, Saint Louis University, St. Louis, MO 63110, USA

## Abstract

Circular RNAs (circRNAs) are newly discovered incipient non-coding RNAs with potential roles in disease progression in living organisms. Significant reports, since their inception, highlight the abundance and putative functional roles of circRNAs in every organism checked for, like* O. sativa*,* Arabidopsis*, human, and mouse. CircRNA expression is generally less than their linear mRNA counterparts which fairly explains the competitive edge of canonical splicing over non-canonical splicing. However, existing methods may not be sensitive enough for the discovery of low-level expressed circRNAs. By combining template-dependent multiple displacement amplification (tdMDA), Illumina sequencing, and bioinformatics tools, we have developed an experimental protocol that is able to detect 1,875 novel and known circRNAs from* O. sativa*. The same method also revealed 9,242 putative circRNAs in less than 40 million reads for the first time from the* Nicotiana benthamiana* whose genome has not been fully annotated. Supported by the PCR-based validation and Sanger sequencing of selective circRNAs, our method represents a valuable tool in profiling circRNAs from the organisms with or without genome annotation.

## 1. Introduction

Circular RNA (circRNAs) is an emerging class of non-coding RNA that attracts significant attention in scientific community. They are covalently closed without the 5′ cap and polyadenylation in the 3′ end. CircRNAs were initially described from plant viroids [1] and subsequently identified in human and visualised by electron microscopy over 35 years ago [2]. Consequent work in sex-determining region Y (SRY) gene was recognised as splicing errors [3, 4]. However, studies on antisense non-coding RNA in INK4 locus (ANRIL) [5] and cerebellar degeneration-related protein 1 (CDR1) [6] have provided compelling evidence for circRNA in human [7], mammals [8], archaea [9],* C. elegans*, and mice [10]. CircRNAs are synthesized by backsplicing of downstream donor site with the upstream acceptor site using the canonical spliceosomal signals and machinery [11, 12]. Recently ever growing reports on circRNAs reveal their pivotal roles in fundamental biological pathways by their multiple functional aspects, such as microRNAs (miRNAs) sponges [10, 13–17], cap-independent translation [18–21], modulation of cellular proliferation [19, 22–24], scaffolding the protein activity [12, 25, 26], and competition with linear mRNAs [19].

With the assistance from computational algorithms [7, 8, 27–32], numerous approaches have been developed to detect and validate the presence of circRNAs in different species across the kingdoms [12, 15, 33, 34]. A major challenge in circRNA discovery might be attributed to its extremely low abundance in samples. The cutting-edge method involves the enrichment of circRNA by enzymatic digestion, RNA-Seq, and bioinformatics identification, followed by PCR-based validation [13, 35, 36]. A limitation in this approach is the sensitivity because library preparation in next-generation sequencing (NGS) is often associated with the loss of low-abundant molecules [12, 37]. Thereby significant sequencing depth is required in order to identify putative circRNAs [12]. In the present study, we have introduced a step of template-dependent multiple displacement amplification (tdMDA) prior to library preparation. Together with a newly developed computer program, we have built an experimental pipeline that shows an enhanced sensitivity to identify circRNAs from the plants.

## 2. Materials and Methods

### 2.1. Plant Materials

*O. sativa* plants were grown in green house maintained at 32°C and (70-80) % humidity for 2 months. Similarly,* N. benthamiana* were grown in plant growth chamber with 16 hrs/8hrs light/dark condition and 85% humidity for 2 months. Plant leaves at 30- and 45-day old were collected for RNA isolation from* O. sativa* and* N. benthamiana,* respectively.

### 2.2. RNA Extraction, Reverse Transcription (RT), and Template-Dependent Multiple Displacement Amplification (tdMDA)

Total RNA was extracted from approximately 100 mg of leaves of* N. benthamiana* and* O. sativa* using Tri Reagent (Sigma, St. Louis, MO, USA) according to the manufacturer's instruction (TRI Reagent (T9424)-Technical bulletin). Extracted RNA was treated with 4U of Turbo DNase (2U/*μ*L, Ambion, Austin, TX, USA) at 37°C for 30 minutes and then inactivated at 72°C for 10 minutes, followed by phenol/chloroform purification. Ten microgram of DNase-treated RNA was subjected to 10U of RNase R (20U/*μ*L, Epicentre, Madison, WI, USA) digestion at 37°C for 15 minutes. Both integrity and concentration were determined respectively on 1.2% agarose gel and Nanodrop ND-1000 (Thermo Scientific, Waltham, MA, USA).

RNA amplification was achieved by RT-tdMDA protocol [38]. In that protocol, background amplification in MDA was eliminated by using exo-resistant random pentamer primers with their 5′ ends blocked by C18 spacer [38]. Its efficiency was first evaluated by using extracted RNA and plasmid APTR9 [39, 40] with the final primer concentration ranging from 50 to 200 *μ*M. About 1 *μ*g of RNase R-treated RNA was converted into cDNA using RevertAid or RevertAid H minus first strand cDNA synthesis kit according to manufacturer's instructions (Thermo Scientific, Waltham, MA, USA). Approximately 50 ng of converted cDNA was directly used for tdMDA in a 20-*μ*L reaction consisting of 2 *μ*l of 10 mM dNTP mix, 2 *μ*l of 10X reaction buffer, 2 *μ*l of 500 *μ*M 5′end-blocked exo-resistant random pentamer primers, 0.6 *μ*l of Phi29 DNA polymerase (10U/*μ*l, Thermo Scientific, Waltham, MA, USA), and 2 *μ*l of pyrophosphatase (0.01U/*μ*l) (Thermo Scientific, Waltham, MA, USA). The reaction mixture was incubated at 28°C for 18 hours and terminated by heating at 65°C for 10 minutes. An aliquot of 3 *μ*l reaction was loaded on 1% TAE agarose gel to check for tdMDA performance.

### 2.3. Identification of circRNA from the tdMDA Amplicons by Cloning and Sequencing

The tdMDA amplicon was randomly digested with the restriction enzymes,* Sac*I,* Hind*III,* Ssp*I,* Bam*HI,* Eco*RV, and* Eco*RI (10U/*μ*l, Thermo Scientific, Waltham, MA, USA) for 3 hours at 37°C. The* Hind*III,* Sac*I digested fragments were purified by GeNei PCR purification kit (Bangalore, Karnataka, India) and cloned in pOK12 or pBluescript II KS (+) vector at the corresponding site at 16°C for 12 hours. After the confirmation by restriction digestion, a total of nine clones were sequenced, seven for* N. benthamiana* and two for* O. sativa*. The mapped clone sequences such as* Hind*III 10,* Hind*III 33,* Hind*III 38, and* Sac*I 11 for* N. benthamiana* and* Hind*III 1 and* Hind*III 2 for* O. sativa* were subjected to prediction for their possibility of forming circRNA in PlantcircBase [41] (http://ibi.zju.edu.cn/plantcircbase/index.php). Predicted putative circRNA sequences were validated by RT-PCR with divergent primers (Table 1) [42].

### 2.4. Identification of circRNA from the tdMDA Amplicons by Illumina Sequencing

About 200 ng of tdMDA products was used for library construction using Illumina-compatible NEXTflex Rapid DNA sequencing kit (BIOO Scientific, Austin, Texas, USA) according to manufacturer's instructions, followed by sequencing at the Illumina NextSeq 500 platform (150-nt paired end) at Genotypic Technology, Bangalore as previously described [40, 43]. Under genomic annotations from Ensembl plant release 29 [44], trimmed reads at Phred 23 were aligned with* O. sativa Indica* genome and* N. benthamiana* draft genome for subsequent circRNA identification using DCC software (v 0.4.4) [45]. In addition to the consideration of non-canonical splice junction, other parameters were also included for circRNA identification as postulated in the DCC [45]. All the analysis was carried out using Biolinux 8 OS [46].

### 2.5. Validation of circRNAs Derived from tdMDA-Illumina Sequencing

Divergent primers were designed from the circRNA derived from NGS-tdMDA containing the junction site (Table 1). Most primers designed for* O. sativa* and* N. benthamiana* were tested for the validation of corresponding circRNAs using genomic DNA and cDNA by the standardised annealing temperature (T_A_). Divergent PCR products were subjected to sequencing or digestion with restriction enzymes.

### 2.6. Northern Hybridization

Non-radioactive northern hybridization was performed with the purified PCR fragment (>200 nt) as the probe, which spanned the corresponding circRNA junction site. Probe preparation was followed with the DIG DNA labelling kit (Roche, Basel, Switzerland) according to manufacturer's instructions.

### 2.7. Analysis on circRNA Conservation and miRNA Binding Sites

NCBI-BLASTN was used to examine the conservation of circRNAs with other reported plant species, such as* S. bicolour* [47],* S. italica* [47],* B. distachyon* [47], and those included in plant circular RNA database [41]. The psRNATarget, a plant small RNA target analysis server (2017 release) [48], was applied to annotate the possible role of reported* O. sativa* and* N. tabacum* miRNAs on predicted circRNAs.

## 3. Results

### 3.1. The Elimination of Background Amplification by tdMDA

Template independent amplification (TIA) in MDA is a major concern owing to high concentrations of random hexamers and an extended incubation period [38]. To eliminate TIA, we followed the protocol proposed by Wang et al. [38]. Total RNA extracted from* O. sativa* was mixed with the plasmid pAPTR9 that harboured* Bhendi yellow vein mosaic virus* (BYVMV). After DNase treatment, BYVMV-specific PCR yielded no amplification, suggesting a complete DNA digestion in the template (Figure S1a). This template was subsequently used to test the efficiency of RT-tdMDA protocol [38]. In four primer concentrations (50, 100, 150, and 200 *μ*M), no amplicon was found in the controls (no template) (Figure S1b). In contrast, 50 ng of template along with 50 *μ*M final primer concentration showed an apparent amplification (Figure S1b). Therefore, the use of exo-resistant random pentamer primers with blocked 5′ ends efficiently eliminates TIA.

### 3.2. Novel circRNAs Identified by RT-tdMDA, Cloning, and Sanger Sequencing

**Figure 1:**
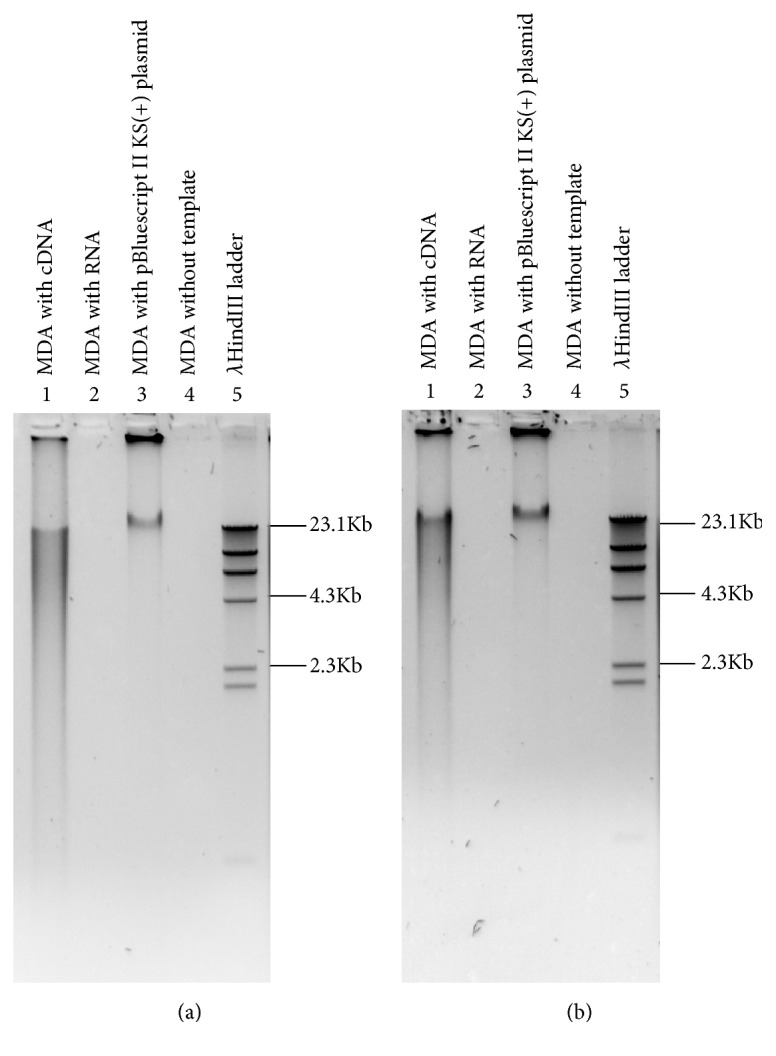
**Amplification of cDNA by Phi29 DNA polymerase**. Total RNA from* N. benthamiana* (a) and* O. sativa* (b) was treated with DNase and RNase R to enrich circRNAs. The enriched circRNAs were converted into cDNA using random hexamer and subjected to amplification by Phi29 DNA polymerase.

After DNase and RNase R treatment (Figure S2), total RNA from* N. benthamiana* and* O. sativa* plants was successfully amplified (Figure 1). Again, no amplification was observed from the negative controls (no template) (Figure 1). Of seven sequenced clones derived from* N. benthamiana*, four sequences, named* Sac*I 11,* Hind*III 10,* Hind*III 33, and* Hind*III 38, showed 100% sequence identity in BLAST analysis against* N. benthamiana* genome [49] (https://solgenomics.net/organism/Nicotiana_benthamiana/ genome). Two clones from* O. sativa*,* Hind*III 1 and* Hind*III 2, were aligned onto the intron region in chromosomes 7 and 1 of* O. sativa* with 100% and 99% identity respectively. These sequences were then analyzed in PlantcircBase for the potential of circRNA formation. As a result, three sequences from* N. benthamiana*,* Hind*III 10,* Hind*III 33, and* Sac*I 11, were predicted to be putative circRNAs. The* Hind*III 10 sequence was partially mapped onto the intron domain of N3 disease resistance protein gene of* Nicotiana paniculata* with 96% sequence identity (Figure S3, Figure S5, and Figure S6), suggesting its intronic nature. The* Hind*III 33 sequence was aligned to multiple domains, including the unannotated region of the retrotransposon of* Nicotiana tabacum* (1-156 bp, 88% identity), Frigida like protein gene of* N. benthamiana* (156-259 bp, 87% identity), and 40S ribosomal protein gene (227-282 bp, 94% identity). Therefore,* Hind*III 33 sequence might be an intronic-exonic circRNA (Figure S3, Figure S5 and Figure S6). The* Sac*I 11 sequence (Acc. No. MF066173) was predicted as a circRNA. However, this sequence was mapped onto* Nicotiana sylvestris *mitochondrial genome and thus not included for further experimentation. Analysis in PlantcircBase also suggested that two clones from* O. sativa, Hind*III 1 (osa_circ_032545) and* Hind*III 2 (osa_circ_000547), were existing intronic and exon-intronic circRNAs respectively. For putative circRNA sequences* Hind*III 10 and* Hind*III 33, PCR amplification with divergent primers provided a positive result when cDNA was used as template whereas no amplification was observed at the same size when various concentration of genomic DNA was used as template (Figure S4).

### 3.3. Complete circRNA Profiles Revealed by Illumina Sequencing

**Figure 2:**
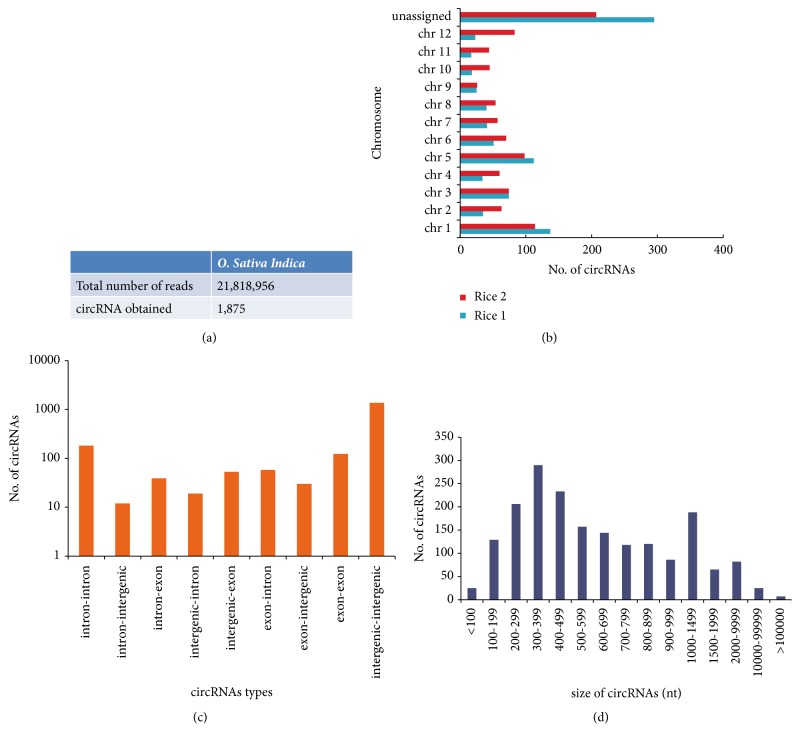
**Identification of rice circRNAs from NGS data.** CircRNAs identified from total number of reads obtained in rice (a); their chromosome wise distribution (b); types (c); size distribution (d).

**Figure 3:**
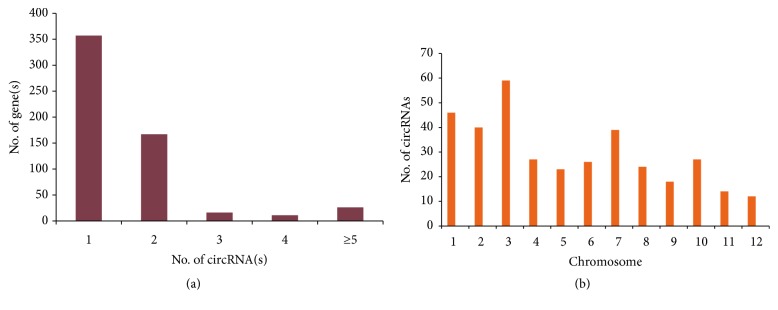
**Rice circRNAs analysis.** Number of rice genes giving circRNA(s) (a); number of overexpressed circRNAs than their linear counterparts across chromosomes (b).

Encouraged by the positive outcome from the cloning and Sanger sequencing, the amplicons from RT-tdMDA were subjected to Illumina sequencing for possible capture of entire circRNA repertoire. The total number of 150-nt paired end reads obtained from* O. sativa* and* N. benthamiana* were 21,818,956 and 38,060,238, respectively. Using the raw reads from the* O. sativa*, the DCC computational pipeline discovered thousands of circular splicing events that yielded 1,875 circRNAs (Figure 2(a)). These putative circRNAs are predominantly distributed on the chromosomes 1 and 5 (Figure 2(b)). Perhaps due to the unannotated genome of Indian cultivar (Pusa Basmati 1), around 200-300 circRNAs came from the genes without any particular chromosome assignment (Figure 2(b)). With respect to circRNA types, the intergenic-intergenic type (n=1,359) was the most abundant type followed by the intronic-intronic type (n=182) and the exonic-exonic type (n=123) (Figure 2(c)). Furthermore, 79% of putative circRNAs had the length between 100 and 999 nt whereas ~1% and ~20% had the size below 100 nt and larger than 1000 nt respectively (Figure 2(d)). The smallest circRNA was found to be of 32 nt between positions 5,187-5,219 in the genome. This putative circRNA is surprisingly an intergenic-intergenic type with CT/AC splice junction on scaffold ID AAAA02040137.1. On the other hand, the largest size of circRNA identified was 737,782 nt on the chromosome 11 between positions 18852424 and19590206, which harbours many functional genes like MIR genes, tRNA genes, and the genes encoding for hypothetical protein. The largest circRNA is assumingly formed in a non-canonical manner and categorised as an intron-intergenic type. Finally, all putative circRNAs were associated with a total of 578 genes in which ~72% had the translation of hypothetical proteins. The gene ID BGIOSGA000405 on the chromosome 1 contributed the maximum number of circRNAs (n=35) while most genes gave only one or two circRNAs (Figure 3(a)). Individually, less than 10% of the genes could produce more than two circRNAs.

**Figure 4:**
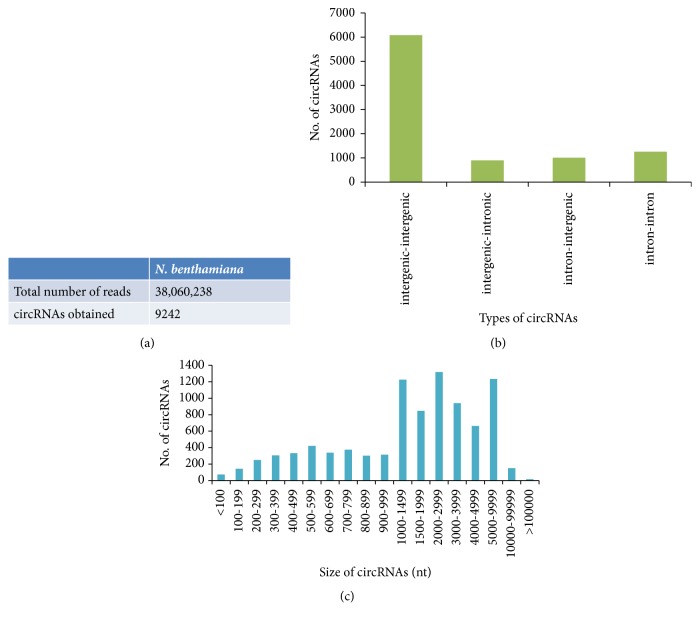
**Identification of* N. benthamiana* circRNA from NGS data.** CircRNAs identified from total number of reads obtained in* N. benthamiana* (a); their types (b); size distribution (c).

Similar analysis in* N. benthamiana* yielded 9,242 circRNAs, including 6,080 intergenic-intergenic, 1,257 intron-intron, 1,009 intron-intergenic, and 896 intergenic-intronic circRNAs (Figures 4(a) and 4(b)). Interestingly, no exonic circRNAs were identified probably because of unavailability of complete genome annotation. In comparison with* O. sativa*, circRNAs from* N. benthamiana* were larger in size with 69% of total identified circRNAs above 1,000 nt (Figure 4(c)). The Niben101Scf02816, an intergenic-intergenic circRNA formed by the non-canonical splice junction, had the smallest size with about 35 nt located between positions 85,132, and 85,167 on the genome. The longest circRNA was Niben101Scf03154 with 299,801 nt, also an intergenic-intergenic type between 589 and 300,390 genome positions.

### 3.4. Validation of circRNAs Identified by RT-tdMDA and Illumina Sequencing

**Figure 5:**
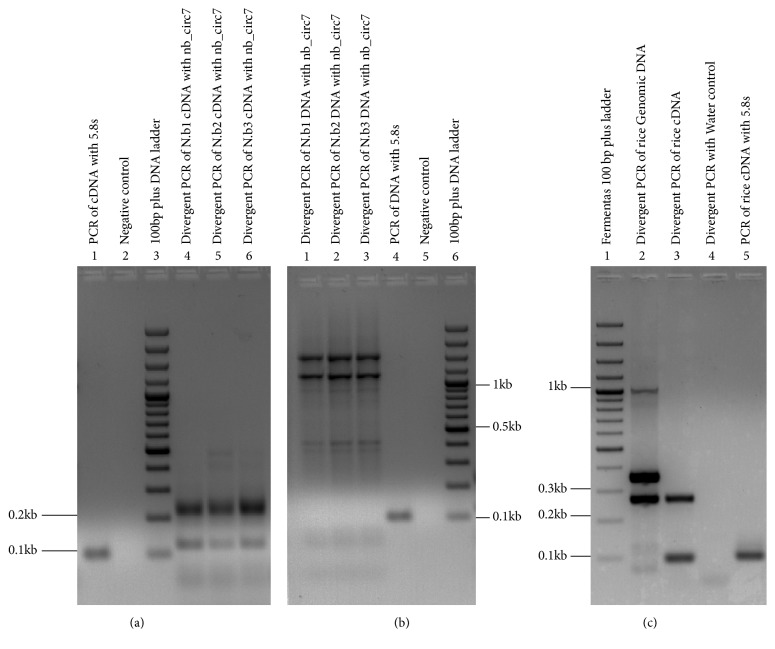
**Divergent PCR for validation of NGS-tdMDA derived circRNA**. Two products (>100bp and >200bp) were amplified with nb_circ7 primer upon divergent PCR (lane 4,5,6) with three* N. benthamiana* cDNA (a). With same primer, it did not give same size amplicon with genomic DNA (lane 1,2,3) as template (b). Two amplicons at ~100bp and 270 bp formed from rice cDNA (lane3) with osi_circ10 divergent primer whereas non-specific amplicon also formed when taking genomic DNA as template (lane 2) (c). Non-template PCR was taken as negative control (lane 2 in (a), lane 5 in (b), and lane 4 in (c)) and 100 bp amplicon formed from 5.8s as positive control (lane 1 in (a), lane 4 in (b), and lane 5 in (c)). Generuler 100 bp plus ladder in lane 3 (a), lane 6 (b), and Fermentas 100 bp ladder in lane 1 (c).

PCR and northern hybridization were used for the validation of selective circRNAs. For circRNA Niben101Scf27324 (Nb_circ7 primer, Table 1), PCR produced two distinct DNA bands with the sizes at ~150 and ~250 bp. There was no DNA amplification at similar sizes upon the use of the genomic DNA as template (Figures 5(a) and 5(b)). Sanger sequencing of PCR product mapped the larger fragment to the circRNA with extra sequence, suggesting a potential alternative splicing event involved for the biogenesis of this particular circRNA (Figure S7). Further evidence came from the northern hybridization that signalled an apparent presence of circRNA Niben101Scf27324 (Figure S8). Divergent PCR also gave positive amplification for the osi_circ10 (Figure 5(c)) as well as other putative circRNAs including Nb_circ3, Nb_circ6, osi_circ2, osi_circ4, osi_circ6, and osi_circ8 (data not shown). Their authenticity was supported by restriction digestion of purified DNA bands from the gel (data not shown).

### 3.5. CircRNAs Are Conserved across the Species

**Figure 6:**
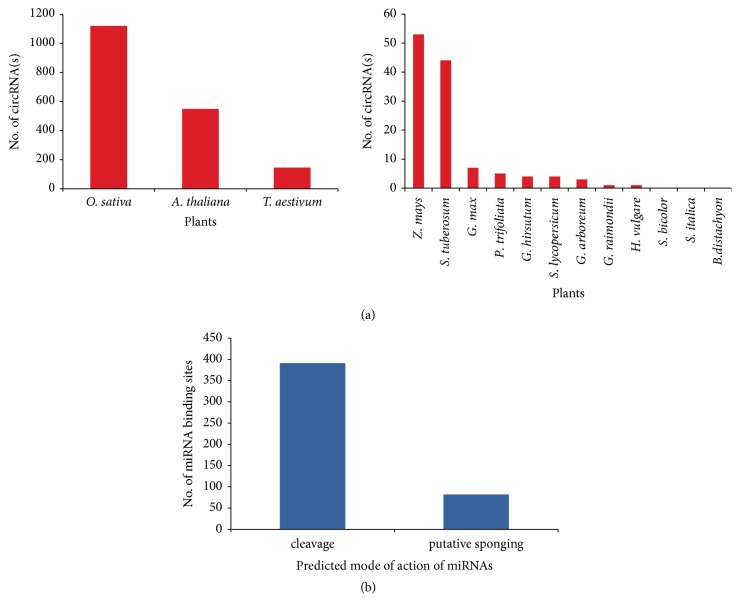
**CircRNA conservation and miRNA action in rice**. Conservation of 1,875 predicted Indica circRNAs against reported circRNA from fifteen plants (a); mode of action of reported Japonica miRNAs on predicted circRNAs (b).

**Figure 7:**
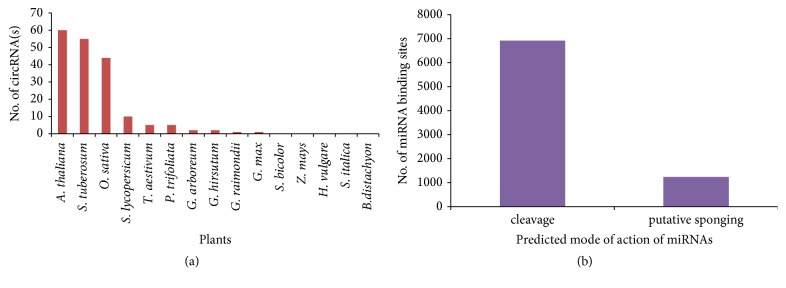
**CircRNA conservation and miRNA action in* N. benthamiana*.** Conservation of predicted 9,242 circRNAs against reported circRNAs from fifteen plants (a); mode of action of reported* N. tabacum* miRNAs on predicted circRNAs (b).

Several reports claimed conservative nature of circRNA across species [8]. Therefore, we compared our circRNAs with all circRNAs either reported [15] or deposited in the plant circular RNA database [41]. Of 1,875 cicrRNAs from the* O. sativa*, significant similarity was found for 1,120 (60%) to* O. Sativa* ssp.* Nipponbare, *549 (29.2%) to* A. thaliana,* and 145 (7.75%) to* T. aestivum* (Figure 6(a)). For* N. benthamiana*, the sequence similarity was also shared for 55 circRNAs with* S. tuberosum,* 60 circRNAs with* A. thaliana,* and 44 circRNAs with* O. Sativa* (Figure 7(a)). There was no or little conservation between our putative circRNAs and the circRNAs discovered in the plants like* S. bicolour*,* S. italica*,* B. distachyon, H. vulgare*, and* P. trifoliata.* This is probably due to a rare number of the circRNAs identified from these plants that could not provide a full scenario to explore the conservation (Table 2).

## 4. Discussion

CircRNAs encompass a transcript family with distinctive structures. Various methods are used to detect the circRNAs in both plants and animals [10, 13]. The difficulty in circRNA identification lies in the inability to separate the circRNAs from other RNA species based on their size or electrophoretic mobility. Molecular techniques that involve amplification or fragmentation may destroy their circular nature since circRNAs lack a free 5′ or 3′ end. Likewise, methods that use polyadenylation ends, such as rapid amplification of cDNA ends (RACE) or poly (A) enrichment, cannot be employed for circRNA identification. These hindrances have been overcome by the emergence of exonuclease based enrichment procedures and high throughput sequencing techniques [12]. For instance, RNA sequencing has been used for the identification of circRNAs in* Arabidopsis* and* O. sativa* [14]. However, owing to an extremely low expression of circRNAs comparing to their linear mRNA counterparts, a high sequencing depth is demanded for productive capture of circRNAs. In order to improve the sensitivity, we exploited the use of tdMDA to identify circRNAs from the* N. benthamiana* and* O. sativa* plants.

Our data have demonstrated the feasibility of tdMDA in the discovery of circRNAs from small amount of RNA samples. First, both novel and known circRNAs were identified from RT-tdMDA product by random enzymatic digestion, cloning, and Sanger sequencing. Two novel circRNAs from the* N. benthamiana* were validated by divergent PCR and had no significant similarity with* Arabidopsis* and other plant candidates. The function of newly identified circRNAs from* N. benthamiana* has to be deciphered. Second, Illumina sequencing of the RT-tdMDA product and bioinformatics analysis captured 1,875 and 9,242 putative circRNAs from* O. sativa* and* N. benthamiana* respectively. The authenticity of selective cirRNAs was confirmed by PCR and northern hybridization. Using RNA sequencing, Jakobi et al. [50] reported the prediction of 575 circRNAs from 33.5 million reads in adult mice heart. Assuming a similar abundance among the samples, we could be able to identify much higher number of circRNAs from almost equal number of reads using the same computational pipeline. Earlier, Jeck and Sharpless (2013) stressed on the need of having 300,000-300,000,000 reads using traditional sequencing to get a single circRNA event whereas exonic circRNA is thought to present roughly 1% of poly(A) RNA [12, 31]. Again, Wang et al. in 2017 analyzed over 90 million raw reads and could obtain only 88 circRNAs. Analyzing say, more than 500 million reads will surely increase the chances of getting low abundance circRNAs but it will spike up the overall cost tremendously. Our method reduces the cost significantly without compromising on findings of lowly expressed circRNAs. Finally, the conservative nature of most predicted circRNAs across the plants further suggests the methodological reliability.

Besides tdMDA, our experimental pipeline also takes the power of the bioinformatics tool. We have applied the DCC that gives the expression count of putative circRNAs as well as the linear RNAs expected from the same genome positions. Interestingly, 19% of* O. sativa* circRNAs showed overexpression with respect to their linear counterparts (Figure 3(b)). Functional aspects of circRNAs have not been fully understood in spite of the reports for their roles in miRNA sponging, transcriptional inhibition and protein formation [12]. Our analysis revealed there are 33 circRNAs that bind to 156 miRNAs in* O. sativa Nipponbare* (Table S1). This number is translated into 473 miRNA binding sites as a single circRNA can bind to more than one miRNA or* vice versa* (Table S2). Of the 473 miRNA binding sites, 391 sites (~83%) are cleavage specific while the remaining 82 sites (~17%) are possibly getting sponged by their targets (Figure 6(b)). In* N. benthamiana, *2,099 circRNAs could have 8,149 miRNA binding sites on 163 published* N. tabacum* miRNAs (Table S3 and S4). Approximately 85% (n=6,916) of miRNA binding sites are cleavage specific and 15% (n=1,233) are target inhibitory in action that need to be deciphered (Figure 7(b)).

## 5. Conclusion

In summary, through the combination of tdMDA and bioinformatics tools, we have established an experimental protocol to detect circRNAs from plant samples. Currently, efficient circRNA discovery requires the treatment of RNA sample by DNase and RNase R which are often associated with the abundance loss of RNA species including circRNAs. Our method is thus particularly useful in working with limited amount of biological samples. A comprehensive profiling of circRNAs, as illustrated in* O. sativa* and* N. benthamiana*, represents an essential step toward biological understating of circRNAs in plants as well as other organisms. This is the first report of circRNA identification from the model plant,* N. benthamiana.*

## Figures and Tables

**Table 1:** List of divergent primers designed for use in confirmation of putative circRNA.

**Divergent primer**	**Sequence **
*Hind*III 10	Forward: 5′-CTATAGTTGAAGCACCTGATGGTGT-3′
	Reverse: 5′-GAGCCATAAAGATAGGCAGTAACTACA-3′

*Hind*III 33	Forward: 5′-TGGTTCACCACAACCCGT-3′
	Reverse: 5′-TGTGTGACTCAAGTTCTCAGTTTGTAA-3′

osi_circ1 (1:36416264-36418547)	Forward: 5′-TGGTAGCAACCGCACAAA-3′
	Reverse: 5′-ATGCTTCCAGGCACATCA-3′

osi_circ2 (2:19273316-20009087)	Forward: 5′-GGGAGCTCAAGGTGAAGAT-3′
	Reverse: 5′-GTTGAACAAACAACACACAAC-3′

osi_circ3 (8:24552647-24573025)	Forward: 5′-ACGTTGAGAGTAAGTTTCCG-3′
	Reverse: 5′-CCCTTTACGATACCACTAGCC-3′

osi_circ4 (12:16650523-17328210)	Forward: 5′-TAGGCTCACGATGTGTTGC-3′
	Reverse: 5′-CGATGAGGGCTGCGAAC-3′

osi_circ5 (9:15720676-15721227)	Forward: 5′-ATCCTTGGAGCTGGCTATGA-3′
	Reverse: 5′-ATCTCGGTTGACCACACACT-3′

osi_circ6 (7:15534138-15534682)	Forward: 5′-TCAAGTCCGCCGTCAAATC-3′
	Reverse: 5′-CCCAAGGGCAGGTTCTTAC-3′

osi_circ7 (6:27117575-27118530)	Forward: 5′-TGCAGAAACAGCATGGTCA-3′
	Reverse: 5′-ATAGGGTGCAAACCTGTGAG-3′

osi_circ8 (8:4494958-4495647)	Forward: 5′-AGAGTCTCTGGCAGTCTCC-3′
	Reverse: 5′-AACCAGTGACTAGCAACTAAGAA-3′

osi_circ9 (1:41518651-41519075)	Forward: 5′-GCGACCTTACTGCACGAATA-3′
	Reverse: 5′-TTGCAAGCGCAACACAAC-3′

osi_circ10 (8:15854661-15861841)	Forward: 5′-GCTAGCAGGGACAGGTTATC-3′
	Reverse: 5′-CAGAAGACGTGTGTGCCTAT-3′

Nb_circ1 (Niben101Scf01334:583095–583645)	Forward: 5′-CTGGGTCAGTCCTCCATTT-3′
	Reverse: 5′-AGATACGCATGCCTCCAA-3′

Nb_circ2 (Niben101Scf01481:214685-215144)	Forward: 5′-TCAACGTGCTTCCTGAACT-3′
	Reverse: 5′-AAATGCTTGGGTCCTACTCC-3′

Nb_circ3 (Niben101Scf01671:738307-738555)	Forward: 5′-TCTTGTCCCAGTCCAGAGA-3′
	Reverse: 5′-TGTCTCCGCGTGTTAATGT-3′

Nb_circ4 (Niben101Scf01820:33613-33924)	Forward: 5′-GTTGTGCTCATTCCATTGGG-3′
	Reverse: 5′-TGCTTCCTGAGCAAGTTCTG-3′

Nb_circ5 (Niben101Scf01505:317983-318653)	Forward: 5′-CCCAATCCACCTTGATCCTT-3′
	Reverse: 5′-CACGACTGGATTTGGCGATA-3′

Nb_circ6 (Niben101Scf32276:10732-11201)	Forward: 5′-TGGGTACCGAAGTGTACTGT-3′
	Reverse: 5′-AAACCTTGGACCGAGATCAAAT-3′

Nb_circ7 (Niben101Scf27324:1438–11811)	Forward: 5′-TGAGCCATTCGCAGTTTCA-3′
	Reverse: 5′-GGTCGTCTCGTCCCTTCT-3′

Nb_circ8 (Niben101Scf15187:11992–12579)	Forward: 5′-TGGCTAGAATGCGGGTTTC-3′
	Reverse: 5′-ATCTTGAAAGTCGTGGTTTCCT-3′

Nb_circ9 (Niben101Scf09703:266605–266722)	Forward: 5′-GCAGTTGGAGACTTTGAGGT-3′
	Reverse: 5′-TGCCGCAAGGGTGATATG-3′

Nb_circ10 (Niben101Scf11535:99376–100075)	Forward: 5′-ACAGGTAGTCTGTTCCGACA-3′
	Reverse: 5′-AGATGCCGAGGAGTTGGA-3′

**Table 2:** List of circRNAs reported from different plants.

**Plants**	**Total no. of circRNAs**	**Reference**
*O. sativa Japonica*	40311	Chu et al, 2017
*A. thaliana*	38938	Chu et al, 2017
*T. aestivum*	88	Chu et al, 2017
*Z. mays*	3238	Chu et al, 2017
*H. vulgare*	39	Chu et al, 2017
*G. max*	5323	Chu et al, 2017
*S. tuberosum*	1728	Chu et al, 2017
*S. lycopersicum*	1904	Chu et al, 2017
*G. arboreum*	1041	Chu et al, 2017
*G. raimondii*	1478	Chu et al, 2017
*G. hirsutum*	499	Chu et al, 2017
*P. trifoliata*	556	Chu et al, 2017
*S. bicolor*	73	Lu et al, 2015
*S. italica*	113	Lu et al, 2015
*B. distachyon*	26	Lu et al, 2015

## Data Availability

Three cloned sequences that denote putative circRNAs are deposited in the GenBank of NCBI under accession number MF066173 through MF066175. Raw Illumina sequence data were placed under Bioproject ID PRJNA422356 (SRA accession nos. SRX4502417, SRX4502416, SRX4502415, SRX3470454, and SRX3470453).

## Supplementary Materials

Supplementary Materials Table S1: list of 156 miRNAs binding with 33 predicted Indica circRNAs. Table S2: number of miRNA binding sites for each of the 33 Indica circRNAs with their position in the genome. Table S3: number of miRNA binding sites for each of the 2099* N. benthamiana* circRNAs with their position in the genome. Table S4: list of* N. tabacum* miRNAs with their number of binding sites on 2099* N. benthamiana* circRNAs. Figure S1: absence of TIA in MDA. Figure S2: RNA extraction from* N. benthamiana* and* O. sativa* plants. Figure S3: linear and predicted circular maps of the putative circRNAs of* N. benthamiana*. Figure S4: divergent PCR for circRNA confirmation. Figure S5: BLAST analysis of* N. benthamiana* cloned sequences in Sol Genomics Network. Figure S6: BLAST analysis of* N. benthamiana* cloned sequences in NCBI. Figure S7: mapping and validation of* N. benthamiana* circRNA. Figure S8: northern blotting for confirmation of circRNA.
