# Supplementary material for: Circular RNA Profiling by Illumina Sequencing via Template-Dependent Multiple Displacement Amplification
Source: Biomed Res Int. 2019 Jan 28;2019:2756516. doi: 10.1155/2019/2756516 (PMC6369502; doi:10.1155/2019/2756516)
Supplement: Supplementary Materials — Table S1: list of 156 miRNAs binding with 33 predicted Indica circRNAs. Table S2: number of miRNA binding sites for each of the 33 Indica circRNAs with their position in the genome. Table S3: number of miRNA binding sites for each of the 2099 N. benthamiana circRNAs with their position in the genome. Table S4: list of N. tabacum miRNAs with their number of binding sites on 2099 N. benthamiana circRNAs. Figure S1: absence of TIA in MDA. Figure S2: RNA extraction from N. benthamiana and O. sativa plants. Figure S3: linear and predicted circular maps of the putative circRNAs of N. benthamiana. Figure S4: divergent PCR for circRNA confirmation. Figure S5: BLAST analysis of N. benthamiana cloned sequences in Sol Genomics Network. Figure S6: BLAST analysis of N. benthamiana cloned sequences in NCBI. Figure S7: mapping and validation of N. benthamiana circRNA. Figure S8: northern blotting for confirmation of circRNA. [file 2756516.f1.doc]

**Supplemental Tables**

**Table S1.** List of 156 miRNAs binding with 33 predicted Indica circRNAs

| **miRNA** | **CircRNA(s) locus with their chromosome** |
| --- | --- |
| osa-miR5502 | 12:16650523-17328210, 2:19273316-20009087 |
| osa-miR2102-5p | 8:13643045-14361331, 8:13631802-14361340 |
| osa-miR5487 | 11:18852424-19590206 |
| osa-miR5507 | 12:16650523-17328210 |
| osa-miR414 | 1:36416264-36418547, 1:36416434-36418549, 1:36416463-36418129, 12:16650523-17328210, 11:18852424-19590206 |
| osa-miR166c-3p | 8:24552647-24573025, 8:24552872-24572189 |
| osa-miR5489 | 11:18852424-19590206 |
| osa-miR418 | 12:16650523-17328210 |
| osa-miR166j-3p | 8:24552647-24573025, 8:24552872-24572189 |
| osa-miR1862a | 11:18852424-19590206 |
| osa-miR1862b | 11:18852424-19590206 |
| osa-miR169i-5p.1 | 2:19273316-20009087 |
| osa-miR5540 | 11:18852424-19590206 |
| osa-miR1862c | 11:18852424-19590206 |
| osa-miR1862d | 2:19273316-20009087 |
| osa-miR435 | 3:20510077-20521871 |
| osa-miR812a | 2:6016860-6017819, 12:16650523-17328210, 8:13643045-14361331, 8:13631802-14361340, 2:3974871-3976712, 11:18852424-19590206 |
| osa-miR2101-5p | 12:16650523-17328210 |
| osa-miR812b | 2:6016860-6017819, 12:16650523-17328210, 8:13643045-14361331, 8:13631802-14361340, 2:3974871-3976712, 11:18852424-19590206 |
| osa-miR171d-5p | 2:19273316-20009087 |
| osa-miR812c | 2:6016860-6017819, 12:16650523-17328210, 8:13643045-14361331, 8:13631802-14361340, 2:3974871-3976712, 11:18852424-19590206 |
| osa-miR812d | 2:6016860-6017819, 12:16650523-17328210, 8:13643045-14361331, 8:13631802-14361340, 2:3974871-3976712, 11:18852424-19590206 |
| osa-miR812e | 2:6016860-6017819, 12:16650523-17328210, 8:13643045-14361331, 8:13631802-14361340, 2:3974871-3976712, 11:18852424-19590206 |
| osa-miR166b-3p | 8:24552647-24573025, 8:24552872-24572189 |
| osa-miR5150-3p | 2:19273316-20009087 |
| osa-miR812f | 11:18852424-19590206, 7:24752845-25058138 |
| osa-miR5148a | 11:18852424-19590206, 7:24752845-25058138 |
| osa-miR2118c | 12:16650523-17328210 |
| osa-miR5148b | 11:18852424-19590206, 7:24752845-25058138 |
| osa-miR2118d | 12:16650523-17328210 |
| osa-miR2871a-3p | 12:16650523-17328210 |
| osa-miR814a | 2:19273316-20009087 |
| osa-miR5148c | 11:18852424-19590206, 7:24752845-25058138 |
| osa-miR2118e | 12:16650523-17328210 |
| osa-miR814b | 2:19273316-20009087 |
| osa-miR5810 | 2:19273316-20009087, 8:13643045-14361331, 8:13631802-14361340 |
| osa-miR166i-3p | 8:24552647-24573025, 8:24552872-24572189 |
| osa-miR2118f | 12:16650523-17328210 |
| osa-miR814c | 2:19273316-20009087 |
| osa-miR812k | 7:24752845-25058138, 12:16650523-17328210 |
| osa-miR5812 | 2:19273316-20009087, 12:16650523-17328210 |
| osa-miR1846c-5p | 12:492996-496643 |
| osa-miR812l | 7:24752845-25058138, 12:16650523-17328210 |
| osa-miR812m | 7:24752845-25058138, 12:16650523-17328210 |
| osa-miR2118j | 12:16650523-17328210 |
| osa-miR5795 | 12:16650523-17328210 |
| osa-miR5817 | 11:18852424-19590206 |
| osa-miR812p | 11:18852424-19590206, 7:24752845-25058138, 12:16650523-17328210 |
| osa-miR2118m | 12:16650523-17328210 |
| osa-miR5799 | 2:19273316-20009087, 7:24752845-25058138 |
| osa-miR1866-3p | 7:24752845-25058138 |
| osa-miR166a-3p | 8:24552647-24573025, 8:24552872-24572189 |
| osa-miR5832 | 12:16650523-17328210 |
| osa-miR812s | 7:24752845-25058138, 12:16650523-17328210 |
| osa-miR2118o | 12:16650523-17328210 |
| osa-miR2118p | 12:16650523-17328210 |
| osa-miR2118q | 12:16650523-17328210 |
| osa-miR5160 | 11:18852424-19590206 |
| osa-miR2118r | 12:16650523-17328210 |
| osa-miR812v | 11:18852424-19590206, 12:16650523-17328210 |
| osa-miR818a | 7:24752845-25058138, 11:18852424-19590206, 4:34169369-34174124, 4:34169374-34174129, 12:16650523-17328210, 4:11710410-11711496, 2:19273316-20009087 |
| osa-miR5162 | 8:13631802-14361340, 8:13643045-14361331, 2:19273316-20009087 |
| osa-miR818b | 7:24752845-25058138, 11:18852424-19590206, 4:34169369-34174124, 4:34169374-34174129, 12:16650523-17328210, 4:11710410-11711496, 2:19273316-20009087 |
| osa-miR812o-3p | 11:18852424-19590206 |
| osa-miR818c | 7:24752845-25058138, 11:18852424-19590206, 4:34169369-34174124, 4:34169374-34174129, 12:16650523-17328210, 4:11710410-11711496, 2:19273316-20009087 |
| osa-miR1846b-5p | 12:492996-496643 |
| osa-miR818d | 7:24752845-25058138, 11:18852424-19590206, 4:34169369-34174124, 4:34169374-34174129, 12:16650523-17328210, 4:11710410-11711496, 2:19273316-20009087 |
| osa-miR7692-3p | 2:19273316-20009087 |
| osa-miR818e | 7:24752845-25058138, 11:18852424-19590206, 4:34169369-34174124, 4:34169374-34174129, 12:16650523-17328210, 4:11710410-11711496, 2:19273316-20009087 |
| osa-miR1319b | 2:19273316-20009087, 11:18852424-19590206 |
| osa-miR166f | 8:24552647-24573025, 8:24552872-24572189 |
| osa-miR812o-5p | 8:11673138-11747061, 7:24752845-25058138, 2:19273316-20009087 |
| osa-miR6245 | 2:19273316-20009087 |
| osa-miR166m | 8:24552647-24573025, 8:24552872-24572189 |
| osa-miR6248 | 2:19273316-20009087 |
| osa-miR166h-3p | 8:24552647-24573025, 8:24552872-24572189 |
| osa-miR1441 | 8:13643045-14361331, 8:13631802-14361340, 2:19273316-20009087 |
| osa-miR1442 | 2:19273316-20009087 |
| osa-miR1850.3 | 8:13643045-14361331, 8:13631802-14361340, 2:19273316-20009087, 7:24752845-25058138, 6:8886531-8887163, 6:8886533-8887165, 11:18852424-19590206 |
| osa-miR812n-5p | 11:18852424-19590206, 7:24752845-25058138 |
| osa-miR531a | 2:19273316-20009087 |
| osa-miR169f.1 | 2:19273316-20009087 |
| osa-miR529b | 8:11673138-11747061, 7:24752845-25058138 |
| osa-miR2120 | 11:18852424-19590206, 2:19273316-20009087, 12:16650523-17328210, 10:9391464-9418480 |
| osa-miR531b | 2:19273316-20009087 |
| osa-miR531c | 2:19273316-20009087 |
| osa-miR166g-3p | 8:24552647-24573025, 8:24552872-24572189 |
| osa-miR1882a | 12:16650523-17328210 |
| osa-miR1846a-5p | 12:492996-496643 |
| osa-miR1882b | 12:16650523-17328210 |
| osa-miR1882c | 12:16650523-17328210 |
| osa-miR1882d | 12:16650523-17328210 |
| osa-miR1882f | 12:16650523-17328210 |
| osa-miR2878-5p | 2:19273316-20009087 |
| osa-miR1882g | 12:16650523-17328210 |
| osa-miR1882h | 12:16650523-17328210 |
| osa-miR5532 | 8:13631802-14361340, 8:13643045-14361331 |
| osa-miR5073 | CH399504.1:2285-2982 |
| osa-miR5074 | 8:13643045-14361331, 12:16650523-17328210, 8:13631802-14361340 |
| osa-miR5536 | 12:16650523-17328210 |
| osa-miR1882e-3p | 12:16650523-17328210 |
| osa-miR5076 | 5:13940133-13960655 |
| osa-miR2867-5p | 11:18852424-19590206 |
| osa-miR2121a | 8:13631802-14361340, 8:13643045-14361331, 12:16650523-17328210 |
| osa-miR1882e-5p | 12:16650523-17328210 |
| osa-miR2874 | 12:16650523-17328210 |
| osa-miR2121b | 8:13631802-14361340, 8:13643045-14361331, 12:16650523-17328210 |
| osa-miR3979-5p | 10:5370769-5371233 |
| osa-miR166e-3p | 8:24552647-24573025, 8:24552872-24572189 |
| osa-miR2876-3p | 11:18852424-19590206 |
| osa-miR5825 | 8:13643045-14361331, 8:13631802-14361340 |
| osa-miR2931 | 12:15668423-15668877, 12:15668421-15668875 |
| osa-miR5829 | 12:16650523-17328210 |
| osa-miR2919 | 2:19273316-20009087, 8:9838649-9890843 |
| osa-miR2932 | 11:18852424-19590206 |
| osa-miR2275a | 11:18852424-19590206 |
| osa-miR439a | 12:16650523-17328210 |
| osa-miR2275b | 11:18852424-19590206 |
| osa-miR439b | 12:16650523-17328210 |
| osa-miR439c | 12:16650523-17328210 |
| osa-miR2275d | 11:18852424-19590206 |
| osa-miR439d | 12:16650523-17328210 |
| osa-miR821a | 12:16650523-17328210, 8:13631802-14361340, 8:13643045-14361331, CH398305.1:15566-19571, 11:18852424-19590206 |
| osa-miR2871b | 12:16650523-17328210 |
| osa-miR439e | 12:16650523-17328210 |
| osa-miR821b | 12:16650523-17328210, 8:13631802-14361340, 8:13643045-14361331, CH398305.1:15566-19571, 11:18852424-19590206 |
| osa-miR166d-3p | 8:24552647-24573025, 8:24552872-24572189 |
| osa-miR439f | 12:16650523-17328210 |
| osa-miR821c | 12:16650523-17328210, 8:13631802-14361340, 8:13643045-14361331, CH398305.1:15566-19571, 11:18852424-19590206 |
| osa-miR439g | 12:16650523-17328210 |
| osa-miR439h | 12:16650523-17328210 |
| osa-miR439i | 12:16650523-17328210 |
| osa-miR2873b | 2:19273316-20009087 |
| osa-miR166l-3p | 8:24552647-24573025, 8:24552872-24572189 |
| osa-miR2873c | 2:19273316-20009087, 12:16650523-17328210 |
| osa-miR1873 | 7:24752845-25058138 |
| osa-miR169e | 2:19273316-20009087 |
| osa-miR169g | 2:19273316-20009087 |
| osa-miR5539a | 1:37004272-37005266 |
| osa-miR1876 | 8:13631802-14361340, 8:13643045-14361331 |
| osa-miR1435 | 8:24552882-24573260, 8:24553718-24573035, 12:16650523-17328210 |
| osa-miR169h | 2:19273316-20009087 |
| osa-miR5539b | 1:37004272-37005266 |
| osa-miR7695-5p | 8:13643045-14361331, 8:13631802-14361340, 12:16650523-17328210 |
| osa-miR1436 | 2:19273316-20009087, 4:34169374-34174129, 4:34169369-34174124, 7:24752845-25058138, 12:16650523-17328210, 11:18852424-19590206 |
| osa-miR2090 | AAAA02040277.1:1090-1861 |
| osa-miR816 | 11:18852424-19590206, 2:19273316-20009087 |
| osa-miR169j | 2:19273316-20009087 |
| osa-miR1438 | 12:16650523-17328210 |
| osa-miR817 | 12:16650523-17328210 |
| osa-miR169k | 2:19273316-20009087 |
| osa-miR1439 | 11:18852424-19590206, 12:16650523-17328210, 2:19273316-20009087, 7:24752845-25058138 |
| osa-miR169l | 2:19273316-20009087 |
| osa-miR169m | 2:19273316-20009087 |
| osa-miR166k-3p | 8:24552647-24573025, 8:24552872-24572189 |
| osa-miR1857-3p | 2:19273316-20009087 |

**Table S2.** Number of miRNA binding sites for each of the 33 Indica circRNAs with their position in the genome

| **Chromosome no.** | **Genome position of circRNA** | **miRNA binding sites** |
| --- | --- | --- |
| 12 | 16650523-17328210 | 140 |
| 11 | 18852424-19590206 | 66 |
| 2 | 19273316-20009087 | 57 |
| 8 | 13631802-14361340 | 42 |
| 8 | 13643045-14361331 | 40 |
| 7 | 24752845-25058138 | 33 |
| 8 | 24552647-24573025 | 13 |
| 8 | 24552872-24572189 | 13 |
| 4 | 34169369-34174124 | 11 |
| 4 | 34169374-34174129 | 11 |
| CH398305.1 | 15566-19571 | 9 |
| 2 | 3974871-3976712 | 5 |
| 2 | 6016860-6017819 | 5 |
| 4 | 11710410-11711496 | 5 |
| 12 | 492996-496643 | 3 |
| 1 | 37004272-37005266 | 2 |
| 8 | 11673138-11747061 | 2 |
| 1 | 36416264-36418547 | 1 |
| 1 | 36416434-36418549 | 1 |
| 1 | 36416463-36418129 | 1 |
| 10 | 5370769-5371233 | 1 |
| 10 | 9391464-9418480 | 1 |
| 12 | 15668421-15668875 | 1 |
| 12 | 15668423-15668877 | 1 |
| 3 | 20510077-20521871 | 1 |
| 5 | 13940133-13960655 | 1 |
| 6 | 8886531-8887163 | 1 |
| 6 | 8886533-8887165 | 1 |
| 8 | 24552882-24573260 | 1 |
| 8 | 24553718-24573035 | 1 |
| 8 | 9838649-9890843 | 1 |
| AAAA02040277.1 | 1090-1861 | 1 |
| CH399504.1 | 2285-2982 | 1 |

**Table S3.** Number of miRNA binding sites for each of the 2099 *N. benthamiana* circRNAs with their position in the genome

| **Predicted *N. benthamiana* CircRNA ID** | **Genome position of circRNA** | **miRNA binding sites** |
| --- | --- | --- |
| Niben101Scf01681 | 29165-316789 | 184 |
| Niben101Scf03154 | 589-300390 | 183 |
| Niben101Scf01163 | 245033-466988 | 172 |
| Niben101Scf00360 | 167578-426971 | 141 |
| Niben101Scf00360 | 167579-426971 | 141 |
| Niben101Scf04196 | 59672-217066 | 115 |
| Niben101Scf03577 | 22975-202191 | 107 |
| Niben101Scf04914 | 16159-120277 | 85 |
| Niben101Scf06127 | 34787-146013 | 85 |
| Niben101Scf02147 | 34529-186448 | 75 |
| Niben101Scf08622 | 1113-44016 | 75 |
| Niben101Scf02147 | 34722-186641 | 74 |
| Niben101Scf01991 | 400734-513923 | 64 |
| Niben101Scf05247 | 250324-321594 | 59 |
| Niben101Scf01098 | 3908-56324 | 49 |
| Niben101Scf03860 | 436920-487848 | 46 |
| Niben101Scf03860 | 437909-488818 | 45 |
| Niben101Scf04196 | 2069-60495 | 45 |
| Niben101Scf04196 | 60153-84389 | 30 |
| Niben101Scf07850 | 290213-345268 | 29 |
| Niben101Scf12084 | 70383-91601 | 27 |
| Niben101Scf01098 | 2382-56322 | 26 |
| Niben101Scf01098 | 3903-56309 | 26 |
| Niben101Scf02023 | 149122-150939 | 26 |
| Niben101Scf12084 | 68484-91014 | 26 |
| Niben101Scf12084 | 68873-90265 | 26 |
| Niben101Scf12084 | 70771-91985 | 26 |
| Niben101Scf30223 | 3112-59295 | 26 |
| Niben101Scf12084 | 70864-92080 | 24 |
| Niben101Scf03860 | 441269-485109 | 21 |
| Niben101Scf03085 | 650086-665650 | 20 |
| Niben101Scf06437 | 730098-731060 | 20 |
| Niben101Scf08622 | 6419-43846 | 20 |
| Niben101Scf08622 | 6422-43849 | 20 |
| Niben101Scf29276 | 5674-6350 | 20 |
| Niben101Scf29276 | 5820-7356 | 20 |
| Niben101Scf29276 | 5832-7084 | 20 |
| Niben101Scf00182 | 198886-218649 | 19 |
| Niben101Scf03860 | 457186-493753 | 19 |
| Niben101Scf03860 | 457189-493645 | 19 |
| Niben101Scf07690 | 52855-53111 | 19 |
| Niben101Scf04639 | 1014145-1036883 | 18 |
| Niben101Scf04639 | 1014148-1036886 | 18 |
| Niben101Scf04675 | 133818-138702 | 18 |
| Niben101Scf06240 | 58273-58649 | 18 |
| Niben101Scf08030 | 538844-539529 | 18 |
| Niben101Scf08030 | 538848-539533 | 18 |
| Niben101Scf02370 | 424697-425566 | 16 |
| Niben101Scf03860 | 457689-490596 | 16 |
| Niben101Scf03860 | 458317-491192 | 16 |
| Niben101Scf03068 | 6771-24535 | 15 |
| Niben101Scf03860 | 456722-485901 | 14 |
| Niben101Scf03860 | 458313-491188 | 14 |
| Niben101Scf06508 | 106004-120987 | 14 |
| Niben101Scf08519 | 3245-12177 | 14 |
| Niben101Scf14566 | 118546-122670 | 14 |
| Niben101Scf03860 | 457078-489990 | 13 |
| Niben101Scf06674 | 328276-330978 | 13 |
| Niben101Scf16974 | 63055-81613 | 13 |
| Niben101Scf03860 | 457205-490117 | 12 |
| Niben101Scf03860 | 457695-489127 | 12 |
| Niben101Scf03860 | 457719-488781 | 12 |
| Niben101Scf12084 | 89252-93179 | 12 |
| Niben101Scf12084 | 89337-92720 | 12 |
| Niben101Scf12084 | 89497-93197 | 12 |
| Niben101Scf12084 | 89503-93203 | 12 |
| Niben101Scf12084 | 89506-92517 | 12 |
| Niben101Scf12084 | 89506-93324 | 12 |
| Niben101Scf12084 | 89509-93327 | 12 |
| Niben101Scf12084 | 89511-92522 | 12 |
| Niben101Scf12084 | 89580-92442 | 12 |
| Niben101Scf12084 | 89594-93307 | 12 |
| Niben101Scf12084 | 89599-93312 | 12 |
| Niben101Scf12084 | 89603-93485 | 12 |
| Niben101Scf12084 | 89606-93488 | 12 |
| Niben101Scf12084 | 90947-93445 | 12 |
| Niben101Scf12084 | 91318-92601 | 12 |
| Niben101Scf12084 | 91322-92605 | 12 |
| Niben101Scf12084 | 91331-93448 | 12 |
| Niben101Scf12084 | 91339-93456 | 12 |
| Niben101Scf13569 | 24930-26218 | 12 |
| Niben101Scf13569 | 24936-26224 | 12 |
| Niben101Scf01072 | 358718-380958 | 11 |
| Niben101Scf03860 | 484962-500124 | 11 |
| Niben101Scf02023 | 151708-154013 | 10 |
| Niben101Scf03860 | 487133-500074 | 10 |
| Niben101Scf06898 | 202080-203330 | 10 |
| Niben101Scf06898 | 202084-203334 | 10 |
| Niben101Scf01602 | 614894-615556 | 9 |
| Niben101Scf03860 | 483572-493602 | 9 |
| Niben101Scf03860 | 483960-499743 | 9 |
| Niben101Scf03860 | 484735-493926 | 9 |
| Niben101Scf03860 | 485287-494593 | 9 |
| Niben101Scf03860 | 485294-494600 | 9 |
| Niben101Scf03860 | 485333-499684 | 9 |
| Niben101Scf03860 | 491861-500281 | 9 |
| Niben101Scf03860 | 499131-509081 | 9 |
| Niben101Scf03860 | 499135-509085 | 9 |
| Niben101Scf06570 | 295003-298424 | 9 |
| Niben101Scf07650 | 350854-352699 | 9 |
| Niben101Scf07650 | 350860-352705 | 9 |
| Niben101Scf12084 | 89295-92541 | 9 |
| Niben101Scf12084 | 89346-92729 | 9 |
| Niben101Scf12084 | 89518-92488 | 9 |
| Niben101Scf12084 | 89531-93276 | 9 |
| Niben101Scf12084 | 89541-93355 | 9 |
| Niben101Scf12084 | 89547-92431 | 9 |
| Niben101Scf12084 | 89562-92548 | 9 |
| Niben101Scf12084 | 89573-93321 | 9 |
| Niben101Scf12084 | 89575-92574 | 9 |
| Niben101Scf12084 | 89576-93291 | 9 |
| Niben101Scf12084 | 89580-92579 | 9 |
| Niben101Scf12084 | 89582-93330 | 9 |
| Niben101Scf12084 | 89583-93298 | 9 |
| Niben101Scf12084 | 89585-92447 | 9 |
| Niben101Scf12084 | 89593-93475 | 9 |
| Niben101Scf12084 | 89598-93312 | 9 |
| Niben101Scf12084 | 89599-93481 | 9 |
| Niben101Scf12084 | 89604-92581 | 9 |
| Niben101Scf12084 | 89606-93129 | 9 |
| Niben101Scf12084 | 89610-93133 | 9 |
| Niben101Scf12084 | 89771-92464 | 9 |
| Niben101Scf12084 | 89815-92477 | 9 |
| Niben101Scf12084 | 89818-92480 | 9 |
| Niben101Scf12084 | 89960-93416 | 9 |
| Niben101Scf12084 | 90157-93473 | 9 |
| Niben101Scf12084 | 90179-92925 | 9 |
| Niben101Scf12084 | 90216-92674 | 9 |
| Niben101Scf12084 | 90221-92679 | 9 |
| Niben101Scf12084 | 90381-93299 | 9 |
| Niben101Scf12084 | 90386-93304 | 9 |
| Niben101Scf12084 | 90451-92899 | 9 |
| Niben101Scf12084 | 90459-92907 | 9 |
| Niben101Scf12084 | 90472-92919 | 9 |
| Niben101Scf12084 | 90579-93307 | 9 |
| Niben101Scf12084 | 90747-93326 | 9 |
| Niben101Scf12084 | 90773-92858 | 9 |
| Niben101Scf12084 | 90778-92863 | 9 |
| Niben101Scf12084 | 90782-93275 | 9 |
| Niben101Scf12084 | 90787-93280 | 9 |
| Niben101Scf12084 | 90804-93295 | 9 |
| Niben101Scf12084 | 90805-92532 | 9 |
| Niben101Scf12084 | 90808-93299 | 9 |
| Niben101Scf12084 | 90870-93428 | 9 |
| Niben101Scf12084 | 90883-92658 | 9 |
| Niben101Scf12084 | 90926-92524 | 9 |
| Niben101Scf12084 | 90940-93144 | 9 |
| Niben101Scf12084 | 90945-93149 | 9 |
| Niben101Scf12084 | 90946-93194 | 9 |
| Niben101Scf12084 | 90946-93335 | 9 |
| Niben101Scf12084 | 90947-93241 | 9 |
| Niben101Scf12084 | 90948-93340 | 9 |
| Niben101Scf12084 | 90952-93450 | 9 |
| Niben101Scf12084 | 90955-93344 | 9 |
| Niben101Scf12084 | 90980-93204 | 9 |
| Niben101Scf12084 | 91051-93347 | 9 |
| Niben101Scf12084 | 91054-93350 | 9 |
| Niben101Scf12084 | 91200-93036 | 9 |
| Niben101Scf12084 | 91207-93043 | 9 |
| Niben101Scf12084 | 91337-92373 | 9 |
| Niben101Scf12084 | 91343-92379 | 9 |
| Niben101Scf12084 | 91380-93055 | 9 |
| Niben101Scf12084 | 91382-92986 | 9 |
| Niben101Scf12084 | 91388-93063 | 9 |
| Niben101Scf12084 | 91815-92710 | 9 |
| Niben101Scf12084 | 92078-92637 | 9 |
| Niben101Scf12084 | 92082-92658 | 9 |
| Niben101Scf12084 | 92116-92898 | 9 |
| Niben101Scf14771 | 194340-195564 | 9 |
| Niben101Scf00712 | 908116-909293 | 8 |
| Niben101Scf00712 | 908119-909296 | 8 |
| Niben101Scf00790 | 59476-60371 | 8 |
| Niben101Scf00790 | 59481-60376 | 8 |
| Niben101Scf01556 | 414224-415574 | 8 |
| Niben101Scf01587 | 275520-280086 | 8 |
| Niben101Scf01587 | 275524-280090 | 8 |
| Niben101Scf02370 | 424906-425450 | 8 |
| Niben101Scf03860 | 487003-497188 | 8 |
| Niben101Scf03860 | 487427-498111 | 8 |
| Niben101Scf03860 | 487436-498120 | 8 |
| Niben101Scf03860 | 489505-497993 | 8 |
| Niben101Scf03860 | 489507-497995 | 8 |
| Niben101Scf03860 | 489592-499763 | 8 |
| Niben101Scf03860 | 489593-499764 | 8 |
| Niben101Scf03860 | 492984-500192 | 8 |
| Niben101Scf03860 | 494953-500233 | 8 |
| Niben101Scf03860 | 495860-500486 | 8 |
| Niben101Scf03860 | 496011-508776 | 8 |
| Niben101Scf03860 | 496074-500476 | 8 |
| Niben101Scf03860 | 497861-500240 | 8 |
| Niben101Scf03860 | 497869-500248 | 8 |
| Niben101Scf03860 | 497876-500256 | 8 |
| Niben101Scf03860 | 498043-500253 | 8 |
| Niben101Scf03860 | 498178-500183 | 8 |
| Niben101Scf03860 | 498184-500189 | 8 |
| Niben101Scf03860 | 498291-500133 | 8 |
| Niben101Scf03860 | 498405-500408 | 8 |
| Niben101Scf03860 | 498410-500413 | 8 |
| Niben101Scf03860 | 498901-500497 | 8 |
| Niben101Scf03860 | 498976-508681 | 8 |
| Niben101Scf03860 | 498992-500497 | 8 |
| Niben101Scf03860 | 498995-500500 | 8 |
| Niben101Scf03860 | 499054-500411 | 8 |
| Niben101Scf03860 | 499059-500416 | 8 |
| Niben101Scf03860 | 499059-500419 | 8 |
| Niben101Scf03860 | 499622-500486 | 8 |
| Niben101Scf04675 | 133814-138698 | 8 |
| Niben101Scf05629 | 100495-101945 | 8 |
| Niben101Scf05629 | 100497-101947 | 8 |
| Niben101Scf06249 | 263020-263395 | 8 |
| Niben101Scf06388 | 163140-165094 | 8 |
| Niben101Scf06388 | 163144-165098 | 8 |
| Niben101Scf07488 | 103664-115894 | 8 |
| Niben101Scf07662 | 273998-275395 | 8 |
| Niben101Scf07662 | 274450-276122 | 8 |
| Niben101Scf07662 | 274455-276127 | 8 |
| Niben101Scf07788 | 80443-81540 | 8 |
| Niben101Scf09760 | 79772-80552 | 8 |
| Niben101Scf11891 | 32858-34648 | 8 |
| Niben101Scf11891 | 32859-34649 | 8 |
| Niben101Scf00238 | 437212-438031 | 7 |
| Niben101Scf00238 | 437217-438036 | 7 |
| Niben101Scf00321 | 181314-183725 | 7 |
| Niben101Scf00360 | 166167-168243 | 7 |
| Niben101Scf00360 | 166173-168249 | 7 |
| Niben101Scf00525 | 561585-562099 | 7 |
| Niben101Scf01033 | 83674-84448 | 7 |
| Niben101Scf01033 | 83677-84451 | 7 |
| Niben101Scf01964 | 861045-862258 | 7 |
| Niben101Scf03839 | 447047-449485 | 7 |
| Niben101Scf03860 | 484204-492263 | 7 |
| Niben101Scf03860 | 484673-492724 | 7 |
| Niben101Scf03860 | 484681-492732 | 7 |
| Niben101Scf28139 | 9403-10013 | 7 |
| Niben101Scf00056 | 777462-778794 | 6 |
| Niben101Scf00056 | 777467-778799 | 6 |
| Niben101Scf00225 | 536114-537251 | 6 |
| Niben101Scf00225 | 536115-537252 | 6 |
| Niben101Scf00225 | 536121-537258 | 6 |
| Niben101Scf00360 | 166844-168244 | 6 |
| Niben101Scf00360 | 166910-168306 | 6 |
| Niben101Scf00360 | 167149-168307 | 6 |
| Niben101Scf00360 | 167150-168307 | 6 |
| Niben101Scf00360 | 167171-168328 | 6 |
| Niben101Scf02083 | 193875-194899 | 6 |
| Niben101Scf02792 | 206997-208726 | 6 |
| Niben101Scf03004 | 253297-254239 | 6 |
| Niben101Scf03004 | 253299-254241 | 6 |
| Niben101Scf03506 | 313451-314562 | 6 |
| Niben101Scf03860 | 483630-492526 | 6 |
| Niben101Scf03860 | 483770-491913 | 6 |
| Niben101Scf03860 | 483776-491919 | 6 |
| Niben101Scf03860 | 483833-491946 | 6 |
| Niben101Scf03860 | 483965-499748 | 6 |
| Niben101Scf03860 | 484045-492718 | 6 |
| Niben101Scf03860 | 484049-492722 | 6 |
| Niben101Scf03860 | 484063-493215 | 6 |
| Niben101Scf03860 | 484083-492091 | 6 |
| Niben101Scf03860 | 484170-493639 | 6 |
| Niben101Scf03860 | 484206-493820 | 6 |
| Niben101Scf03860 | 484212-493826 | 6 |
| Niben101Scf03860 | 484215-492274 | 6 |
| Niben101Scf03860 | 484247-493806 | 6 |
| Niben101Scf03860 | 484248-494244 | 6 |
| Niben101Scf03860 | 484407-492802 | 6 |
| Niben101Scf03860 | 484424-495879 | 6 |
| Niben101Scf03860 | 484429-495884 | 6 |
| Niben101Scf03860 | 484511-497330 | 6 |
| Niben101Scf03860 | 484529-498968 | 6 |
| Niben101Scf03860 | 484678-492729 | 6 |
| Niben101Scf03860 | 484683-498994 | 6 |
| Niben101Scf03860 | 484690-492741 | 6 |
| Niben101Scf03860 | 484764-497828 | 6 |
| Niben101Scf03860 | 484870-493332 | 6 |
| Niben101Scf03860 | 484870-499078 | 6 |
| Niben101Scf03860 | 484881-495017 | 6 |
| Niben101Scf03860 | 484958-494624 | 6 |
| Niben101Scf03860 | 484991-493315 | 6 |
| Niben101Scf03860 | 484996-493320 | 6 |
| Niben101Scf03860 | 485005-500046 | 6 |
| Niben101Scf03860 | 485031-493205 | 6 |
| Niben101Scf03860 | 485107-494257 | 6 |
| Niben101Scf03860 | 485107-496269 | 6 |
| Niben101Scf03860 | 485216-497939 | 6 |
| Niben101Scf03860 | 485220-493385 | 6 |
| Niben101Scf03860 | 485220-494799 | 6 |
| Niben101Scf03860 | 485262-493217 | 6 |
| Niben101Scf03860 | 485290-498187 | 6 |
| Niben101Scf03860 | 485291-498188 | 6 |
| Niben101Scf03860 | 485338-499689 | 6 |
| Niben101Scf03860 | 485383-497971 | 6 |
| Niben101Scf03860 | 485391-493512 | 6 |
| Niben101Scf03860 | 485392-493855 | 6 |
| Niben101Scf03860 | 485419-493986 | 6 |
| Niben101Scf03860 | 485549-496671 | 6 |
| Niben101Scf03860 | 485555-493961 | 6 |
| Niben101Scf03860 | 485587-494129 | 6 |
| Niben101Scf03860 | 485626-493824 | 6 |
| Niben101Scf03860 | 485634-493832 | 6 |
| Niben101Scf03860 | 485745-494376 | 6 |
| Niben101Scf03860 | 485773-498806 | 6 |
| Niben101Scf03860 | 485775-498808 | 6 |
| Niben101Scf03860 | 485867-494456 | 6 |
| Niben101Scf03860 | 485948-499287 | 6 |
| Niben101Scf03860 | 485951-499290 | 6 |
| Niben101Scf03860 | 486007-497265 | 6 |
| Niben101Scf03860 | 486043-495924 | 6 |
| Niben101Scf03860 | 486108-494180 | 6 |
| Niben101Scf03860 | 486213-497245 | 6 |
| Niben101Scf03860 | 486356-499471 | 6 |
| Niben101Scf03860 | 486670-496580 | 6 |
| Niben101Scf03860 | 486784-495048 | 6 |
| Niben101Scf03860 | 486884-496346 | 6 |
| Niben101Scf03860 | 486952-496436 | 6 |
| Niben101Scf03860 | 487010-498364 | 6 |
| Niben101Scf03860 | 487012-497062 | 6 |
| Niben101Scf03860 | 487068-498077 | 6 |
| Niben101Scf03860 | 487298-496356 | 6 |
| Niben101Scf03860 | 487302-498087 | 6 |
| Niben101Scf03860 | 487428-498702 | 6 |
| Niben101Scf03860 | 487515-496115 | 6 |
| Niben101Scf03860 | 487590-496858 | 6 |
| Niben101Scf03860 | 487597-496027 | 6 |
| Niben101Scf03860 | 487702-497198 | 6 |
| Niben101Scf03860 | 487706-497202 | 6 |
| Niben101Scf03860 | 487798-496102 | 6 |
| Niben101Scf03860 | 487951-495958 | 6 |
| Niben101Scf03860 | 488092-497045 | 6 |
| Niben101Scf03860 | 488170-498682 | 6 |
| Niben101Scf03860 | 488177-498689 | 6 |
| Niben101Scf03860 | 488227-498713 | 6 |
| Niben101Scf03860 | 488230-498716 | 6 |
| Niben101Scf03860 | 488319-496294 | 6 |
| Niben101Scf03860 | 488352-496492 | 6 |
| Niben101Scf03860 | 488406-499717 | 6 |
| Niben101Scf03860 | 488539-496504 | 6 |
| Niben101Scf03860 | 488573-497207 | 6 |
| Niben101Scf03860 | 488577-498763 | 6 |
| Niben101Scf03860 | 488579-498765 | 6 |
| Niben101Scf03860 | 488582-497216 | 6 |
| Niben101Scf03860 | 488643-499538 | 6 |
| Niben101Scf03860 | 488735-498090 | 6 |
| Niben101Scf03860 | 488740-497549 | 6 |
| Niben101Scf03860 | 488839-499275 | 6 |
| Niben101Scf03860 | 488900-498518 | 6 |
| Niben101Scf03860 | 488902-498520 | 6 |
| Niben101Scf03860 | 489059-497124 | 6 |
| Niben101Scf03860 | 489064-497129 | 6 |
| Niben101Scf03860 | 489086-498563 | 6 |
| Niben101Scf03860 | 489133-499949 | 6 |
| Niben101Scf03860 | 489134-498962 | 6 |
| Niben101Scf03860 | 489140-499956 | 6 |
| Niben101Scf03860 | 489161-497065 | 6 |
| Niben101Scf03860 | 489223-499813 | 6 |
| Niben101Scf03860 | 489226-499816 | 6 |
| Niben101Scf03860 | 489300-497195 | 6 |
| Niben101Scf03860 | 489306-497201 | 6 |
| Niben101Scf03860 | 489342-499894 | 6 |
| Niben101Scf03860 | 489361-499669 | 6 |
| Niben101Scf03860 | 489363-497905 | 6 |
| Niben101Scf03860 | 489459-499793 | 6 |
| Niben101Scf03860 | 489463-499797 | 6 |
| Niben101Scf03860 | 489522-500177 | 6 |
| Niben101Scf03860 | 489528-500183 | 6 |
| Niben101Scf03860 | 489584-497899 | 6 |
| Niben101Scf03860 | 489600-499771 | 6 |
| Niben101Scf03860 | 489654-498306 | 6 |
| Niben101Scf03860 | 489662-498314 | 6 |
| Niben101Scf03860 | 489698-497790 | 6 |
| Niben101Scf03860 | 489721-497813 | 6 |
| Niben101Scf03860 | 489728-497820 | 6 |
| Niben101Scf03860 | 489846-499649 | 6 |
| Niben101Scf03860 | 489848-499651 | 6 |
| Niben101Scf03860 | 489874-497823 | 6 |
| Niben101Scf03860 | 489917-498249 | 6 |
| Niben101Scf03860 | 489924-498256 | 6 |
| Niben101Scf03860 | 489941-498755 | 6 |
| Niben101Scf03860 | 490036-499472 | 6 |
| Niben101Scf03860 | 490044-498274 | 6 |
| Niben101Scf03860 | 490047-498277 | 6 |
| Niben101Scf03860 | 490052-497819 | 6 |
| Niben101Scf03860 | 490060-497827 | 6 |
| Niben101Scf03860 | 490114-498227 | 6 |
| Niben101Scf05017 | 599264-601101 | 6 |
| Niben101Scf05017 | 599268-601105 | 6 |
| Niben101Scf07662 | 273544-275356 | 6 |
| Niben101Scf07662 | 273987-274956 | 6 |
| Niben101Scf07662 | 274011-275334 | 6 |
| Niben101Scf07662 | 274182-275185 | 6 |
| Niben101Scf07662 | 274187-275397 | 6 |
| Niben101Scf07662 | 274218-276374 | 6 |
| Niben101Scf07662 | 274226-276382 | 6 |
| Niben101Scf07662 | 274250-275230 | 6 |
| Niben101Scf07662 | 274261-275241 | 6 |
| Niben101Scf07662 | 274450-275419 | 6 |
| Niben101Scf07662 | 274455-275424 | 6 |
| Niben101Scf07662 | 274502-276234 | 6 |
| Niben101Scf07662 | 274509-276241 | 6 |
| Niben101Scf12670 | 79752-81322 | 6 |
| Niben101Scf12670 | 79755-81325 | 6 |
| Niben101Scf15364 | 99182-99520 | 6 |
| Niben101Scf15364 | 99185-99523 | 6 |
| Niben101Scf00756 | 273932-274493 | 5 |
| Niben101Scf00756 | 273937-274498 | 5 |
| Niben101Scf00870 | 511687-512276 | 5 |
| Niben101Scf01559 | 462885-466457 | 5 |
| Niben101Scf01559 | 462885-466458 | 5 |
| Niben101Scf01559 | 462885-466459 | 5 |
| Niben101Scf01559 | 462888-466462 | 5 |
| Niben101Scf01559 | 462889-466462 | 5 |
| Niben101Scf01559 | 462890-466462 | 5 |
| Niben101Scf01559 | 464932-466458 | 5 |
| Niben101Scf01559 | 464932-466459 | 5 |
| Niben101Scf01559 | 465255-466465 | 5 |
| Niben101Scf01559 | 465263-466473 | 5 |
| Niben101Scf01559 | 465316-466322 | 5 |
| Niben101Scf01559 | 465321-466327 | 5 |
| Niben101Scf01559 | 465425-466017 | 5 |
| Niben101Scf01559 | 465429-466021 | 5 |
| Niben101Scf02232 | 57739-59112 | 5 |
| Niben101Scf02232 | 57880-59102 | 5 |
| Niben101Scf02232 | 57885-59107 | 5 |
| Niben101Scf02513 | 415836-418461 | 5 |
| Niben101Scf02793 | 1415315-1416397 | 5 |
| Niben101Scf03227 | 525683-531463 | 5 |
| Niben101Scf03404 | 42859-49374 | 5 |
| Niben101Scf03460 | 791272-794563 | 5 |
| Niben101Scf03860 | 489487-491005 | 5 |
| Niben101Scf03860 | 489493-491011 | 5 |
| Niben101Scf03860 | 490153-498112 | 5 |
| Niben101Scf03860 | 490202-498364 | 5 |
| Niben101Scf03860 | 490207-498369 | 5 |
| Niben101Scf03860 | 490208-499490 | 5 |
| Niben101Scf03860 | 490345-498024 | 5 |
| Niben101Scf03860 | 490402-499831 | 5 |
| Niben101Scf03860 | 490404-499833 | 5 |
| Niben101Scf04133 | 117962-119900 | 5 |
| Niben101Scf04133 | 117967-119905 | 5 |
| Niben101Scf04196 | 60354-84590 | 5 |
| Niben101Scf07650 | 351470-352709 | 5 |
| Niben101Scf07650 | 351472-352711 | 5 |
| Niben101Scf07662 | 274168-275083 | 5 |
| Niben101Scf07662 | 274483-274970 | 5 |
| Niben101Scf07662 | 274532-274906 | 5 |
| Niben101Scf07662 | 274539-274913 | 5 |
| Niben101Scf10369 | 232409-233145 | 5 |
| Niben101Scf00173 | 472831-473271 | 4 |
| Niben101Scf00173 | 472837-473277 | 4 |
| Niben101Scf00360 | 165858-167579 | 4 |
| Niben101Scf00360 | 165862-167554 | 4 |
| Niben101Scf00360 | 165872-167855 | 4 |
| Niben101Scf00360 | 166169-167456 | 4 |
| Niben101Scf00360 | 166970-168400 | 4 |
| Niben101Scf00360 | 166975-168405 | 4 |
| Niben101Scf00381 | 644924-645972 | 4 |
| Niben101Scf00381 | 644931-645979 | 4 |
| Niben101Scf00870 | 511693-512282 | 4 |
| Niben101Scf02275 | 411837-420954 | 4 |
| Niben101Scf02513 | 416026-418741 | 4 |
| Niben101Scf02793 | 1415313-1416395 | 4 |
| Niben101Scf02812 | 236644-239082 | 4 |
| Niben101Scf03860 | 483753-491555 | 4 |
| Niben101Scf03860 | 483976-491584 | 4 |
| Niben101Scf03860 | 483980-491588 | 4 |
| Niben101Scf03860 | 484290-491927 | 4 |
| Niben101Scf03860 | 484293-491930 | 4 |
| Niben101Scf03860 | 484371-492029 | 4 |
| Niben101Scf03860 | 484377-492035 | 4 |
| Niben101Scf03860 | 484539-492346 | 4 |
| Niben101Scf03860 | 484544-492351 | 4 |
| Niben101Scf03860 | 484817-492691 | 4 |
| Niben101Scf03860 | 485071-492923 | 4 |
| Niben101Scf03860 | 485078-492930 | 4 |
| Niben101Scf03860 | 485320-493042 | 4 |
| Niben101Scf03860 | 485968-493611 | 4 |
| Niben101Scf03860 | 485974-493617 | 4 |
| Niben101Scf03860 | 486824-494659 | 4 |
| Niben101Scf03860 | 487165-494761 | 4 |
| Niben101Scf03860 | 487224-494817 | 4 |
| Niben101Scf03860 | 488217-495957 | 4 |
| Niben101Scf03860 | 488224-495964 | 4 |
| Niben101Scf03860 | 489214-491064 | 4 |
| Niben101Scf03860 | 489218-491068 | 4 |
| Niben101Scf03860 | 489232-491038 | 4 |
| Niben101Scf03860 | 489236-491042 | 4 |
| Niben101Scf03860 | 489328-491251 | 4 |
| Niben101Scf03860 | 489334-491254 | 4 |
| Niben101Scf03860 | 489433-491377 | 4 |
| Niben101Scf03860 | 489438-491382 | 4 |
| Niben101Scf03860 | 489446-491350 | 4 |
| Niben101Scf03860 | 489449-491353 | 4 |
| Niben101Scf03860 | 489487-490837 | 4 |
| Niben101Scf03860 | 489491-490841 | 4 |
| Niben101Scf03860 | 489569-491521 | 4 |
| Niben101Scf03860 | 489594-490970 | 4 |
| Niben101Scf03860 | 489636-491049 | 4 |
| Niben101Scf03860 | 489680-491185 | 4 |
| Niben101Scf03860 | 489691-491208 | 4 |
| Niben101Scf03860 | 489734-491224 | 4 |
| Niben101Scf03860 | 489739-491229 | 4 |
| Niben101Scf03860 | 489755-491176 | 4 |
| Niben101Scf03860 | 489914-497589 | 4 |
| Niben101Scf03860 | 489917-497592 | 4 |
| Niben101Scf03860 | 490780-492761 | 4 |
| Niben101Scf04285 | 144030-145756 | 4 |
| Niben101Scf04285 | 144034-145760 | 4 |
| Niben101Scf04285 | 144423-145407 | 4 |
| Niben101Scf04285 | 144431-145415 | 4 |
| Niben101Scf04422 | 383546-384781 | 4 |
| Niben101Scf04664 | 166523-167762 | 4 |
| Niben101Scf04964 | 39374-43948 | 4 |
| Niben101Scf05017 | 485707-486445 | 4 |
| Niben101Scf05155 | 22554-23399 | 4 |
| Niben101Scf05229 | 44718-51982 | 4 |
| Niben101Scf05862 | 97272-97581 | 4 |
| Niben101Scf05862 | 97278-97587 | 4 |
| Niben101Scf06132 | 57337-58445 | 4 |
| Niben101Scf06132 | 57341-58449 | 4 |
| Niben101Scf07141 | 48329-51248 | 4 |
| Niben101Scf08453 | 15503-31355 | 4 |
| Niben101Scf11130 | 9740-11016 | 4 |
| Niben101Scf11778 | 88867-90231 | 4 |
| Niben101Scf13683 | 130071-131887 | 4 |
| Niben101Scf13683 | 130075-131891 | 4 |
| Niben101Scf18902 | 15127-16046 | 4 |
| Niben101Scf18902 | 15135-16054 | 4 |
| Niben101Scf24272 | 9600-10487 | 4 |
| Niben101Scf24272 | 9605-10492 | 4 |
| Niben101Scf00148 | 131928-132823 | 3 |
| Niben101Scf00397 | 288735-290211 | 3 |
| Niben101Scf00539 | 278295-279649 | 3 |
| Niben101Scf00539 | 278302-279656 | 3 |
| Niben101Scf00870 | 511711-512200 | 3 |
| Niben101Scf00870 | 511712-512201 | 3 |
| Niben101Scf01008 | 216027-217458 | 3 |
| Niben101Scf01008 | 216579-217386 | 3 |
| Niben101Scf01085 | 167311-168395 | 3 |
| Niben101Scf01517 | 793008-793815 | 3 |
| Niben101Scf01964 | 861112-862021 | 3 |
| Niben101Scf01964 | 861117-862026 | 3 |
| Niben101Scf02285 | 162393-163158 | 3 |
| Niben101Scf02285 | 162398-163163 | 3 |
| Niben101Scf02985 | 3952-5812 | 3 |
| Niben101Scf02985 | 4166-5839 | 3 |
| Niben101Scf02985 | 4169-5842 | 3 |
| Niben101Scf02985 | 5635-6275 | 3 |
| Niben101Scf03226 | 172320-176448 | 3 |
| Niben101Scf03351 | 205357-206227 | 3 |
| Niben101Scf03518 | 488502-494729 | 3 |
| Niben101Scf03860 | 483567-493597 | 3 |
| Niben101Scf03860 | 483696-499345 | 3 |
| Niben101Scf03860 | 483934-496108 | 3 |
| Niben101Scf03860 | 484246-493805 | 3 |
| Niben101Scf03860 | 484293-499740 | 3 |
| Niben101Scf03860 | 484294-499741 | 3 |
| Niben101Scf03860 | 484348-494875 | 3 |
| Niben101Scf03860 | 484372-498205 | 3 |
| Niben101Scf03860 | 484401-497041 | 3 |
| Niben101Scf03860 | 484406-493591 | 3 |
| Niben101Scf03860 | 484409-497049 | 3 |
| Niben101Scf03860 | 484414-493599 | 3 |
| Niben101Scf03860 | 484534-495977 | 3 |
| Niben101Scf03860 | 484547-494068 | 3 |
| Niben101Scf03860 | 484555-494076 | 3 |
| Niben101Scf03860 | 484570-494948 | 3 |
| Niben101Scf03860 | 484882-498412 | 3 |
| Niben101Scf03860 | 484883-498413 | 3 |
| Niben101Scf03860 | 484952-494618 | 3 |
| Niben101Scf03860 | 485072-493427 | 3 |
| Niben101Scf03860 | 485078-493433 | 3 |
| Niben101Scf03860 | 485083-493511 | 3 |
| Niben101Scf03860 | 485205-493816 | 3 |
| Niben101Scf03860 | 485220-498984 | 3 |
| Niben101Scf03860 | 485222-497864 | 3 |
| Niben101Scf03860 | 485304-493900 | 3 |
| Niben101Scf03860 | 485310-493906 | 3 |
| Niben101Scf03860 | 485418-495807 | 3 |
| Niben101Scf03860 | 487229-494822 | 3 |
| Niben101Scf03860 | 488962-491260 | 3 |
| Niben101Scf03860 | 489052-490880 | 3 |
| Niben101Scf03860 | 489125-491392 | 3 |
| Niben101Scf03860 | 489137-490875 | 3 |
| Niben101Scf03860 | 489220-491512 | 3 |
| Niben101Scf03860 | 489224-491516 | 3 |
| Niben101Scf03860 | 489225-491518 | 3 |
| Niben101Scf03860 | 489227-491507 | 3 |
| Niben101Scf03860 | 489229-491522 | 3 |
| Niben101Scf03860 | 489351-491514 | 3 |
| Niben101Scf03860 | 489353-491505 | 3 |
| Niben101Scf03860 | 489357-491509 | 3 |
| Niben101Scf03860 | 489358-491521 | 3 |
| Niben101Scf03860 | 489365-491560 | 3 |
| Niben101Scf03860 | 489371-491566 | 3 |
| Niben101Scf03860 | 489587-491738 | 3 |
| Niben101Scf03860 | 489610-491284 | 3 |
| Niben101Scf03860 | 489621-491386 | 3 |
| Niben101Scf03860 | 489626-491262 | 3 |
| Niben101Scf03860 | 489633-491398 | 3 |
| Niben101Scf03860 | 489652-497190 | 3 |
| Niben101Scf03860 | 489658-497196 | 3 |
| Niben101Scf03860 | 489730-490855 | 3 |
| Niben101Scf03860 | 489733-490858 | 3 |
| Niben101Scf03860 | 489801-491628 | 3 |
| Niben101Scf03860 | 489849-491705 | 3 |
| Niben101Scf03860 | 489850-491705 | 3 |
| Niben101Scf03860 | 489854-491709 | 3 |
| Niben101Scf03860 | 489863-491718 | 3 |
| Niben101Scf03860 | 489891-491505 | 3 |
| Niben101Scf03860 | 489897-491263 | 3 |
| Niben101Scf03860 | 489899-491513 | 3 |
| Niben101Scf03860 | 489902-491760 | 3 |
| Niben101Scf03860 | 489903-491722 | 3 |
| Niben101Scf03860 | 489906-491445 | 3 |
| Niben101Scf03860 | 489906-491764 | 3 |
| Niben101Scf03860 | 489915-491628 | 3 |
| Niben101Scf03860 | 489919-491654 | 3 |
| Niben101Scf03860 | 489922-491506 | 3 |
| Niben101Scf03860 | 489924-491659 | 3 |
| Niben101Scf03860 | 489947-491907 | 3 |
| Niben101Scf03860 | 489964-492058 | 3 |
| Niben101Scf03860 | 489972-491883 | 3 |
| Niben101Scf03860 | 489978-492031 | 3 |
| Niben101Scf03860 | 489980-491606 | 3 |
| Niben101Scf03860 | 489980-491948 | 3 |
| Niben101Scf03860 | 489997-491915 | 3 |
| Niben101Scf03860 | 490000-491999 | 3 |
| Niben101Scf03860 | 490004-491896 | 3 |
| Niben101Scf03860 | 490010-491967 | 3 |
| Niben101Scf03860 | 490012-491969 | 3 |
| Niben101Scf03860 | 490029-491663 | 3 |
| Niben101Scf03860 | 490029-491966 | 3 |
| Niben101Scf03860 | 490035-491953 | 3 |
| Niben101Scf03860 | 490040-491417 | 3 |
| Niben101Scf03860 | 490045-491836 | 3 |
| Niben101Scf03860 | 490051-491842 | 3 |
| Niben101Scf03860 | 490057-492083 | 3 |
| Niben101Scf03860 | 490061-492089 | 3 |
| Niben101Scf03860 | 490070-491120 | 3 |
| Niben101Scf03860 | 490078-491711 | 3 |
| Niben101Scf03860 | 490110-491518 | 3 |
| Niben101Scf03860 | 490119-491309 | 3 |
| Niben101Scf03860 | 490141-491549 | 3 |
| Niben101Scf03860 | 490150-491392 | 3 |
| Niben101Scf03860 | 490151-491676 | 3 |
| Niben101Scf03860 | 490155-490989 | 3 |
| Niben101Scf03860 | 490157-491399 | 3 |
| Niben101Scf03860 | 490158-490992 | 3 |
| Niben101Scf03860 | 490161-491761 | 3 |
| Niben101Scf03860 | 490164-491550 | 3 |
| Niben101Scf03860 | 490166-491552 | 3 |
| Niben101Scf03860 | 490168-491768 | 3 |
| Niben101Scf03860 | 490179-491822 | 3 |
| Niben101Scf03860 | 490180-491694 | 3 |
| Niben101Scf03860 | 490187-491701 | 3 |
| Niben101Scf03860 | 490194-492082 | 3 |
| Niben101Scf03860 | 490202-491569 | 3 |
| Niben101Scf03860 | 490203-491801 | 3 |
| Niben101Scf03860 | 490207-492236 | 3 |
| Niben101Scf03860 | 490233-492198 | 3 |
| Niben101Scf03860 | 490234-491749 | 3 |
| Niben101Scf03860 | 490255-491740 | 3 |
| Niben101Scf03860 | 490260-497957 | 3 |
| Niben101Scf03860 | 490289-491919 | 3 |
| Niben101Scf03860 | 490328-491188 | 3 |
| Niben101Scf03860 | 490334-491513 | 3 |
| Niben101Scf03860 | 490336-491590 | 3 |
| Niben101Scf03860 | 490337-491324 | 3 |
| Niben101Scf03860 | 490340-491594 | 3 |
| Niben101Scf03860 | 490341-491520 | 3 |
| Niben101Scf03860 | 490342-491330 | 3 |
| Niben101Scf03860 | 490344-491542 | 3 |
| Niben101Scf03860 | 490347-491541 | 3 |
| Niben101Scf03860 | 490348-491335 | 3 |
| Niben101Scf03860 | 490352-491512 | 3 |
| Niben101Scf03860 | 490352-491550 | 3 |
| Niben101Scf03860 | 490353-491583 | 3 |
| Niben101Scf03860 | 490354-498033 | 3 |
| Niben101Scf03860 | 490362-491522 | 3 |
| Niben101Scf03860 | 490362-491754 | 3 |
| Niben101Scf03860 | 490363-491867 | 3 |
| Niben101Scf03860 | 490368-491249 | 3 |
| Niben101Scf03860 | 490368-491759 | 3 |
| Niben101Scf03860 | 490373-492085 | 3 |
| Niben101Scf03860 | 490375-491766 | 3 |
| Niben101Scf03860 | 490381-492093 | 3 |
| Niben101Scf03860 | 490388-492148 | 3 |
| Niben101Scf03860 | 490390-491714 | 3 |
| Niben101Scf03860 | 490396-491417 | 3 |
| Niben101Scf03860 | 490396-491527 | 3 |
| Niben101Scf03860 | 490399-491530 | 3 |
| Niben101Scf03860 | 490399-491723 | 3 |
| Niben101Scf03860 | 490405-491426 | 3 |
| Niben101Scf03860 | 490408-491142 | 3 |
| Niben101Scf03860 | 490409-491322 | 3 |
| Niben101Scf03860 | 490409-491587 | 3 |
| Niben101Scf03860 | 490410-492141 | 3 |
| Niben101Scf03860 | 490411-491326 | 3 |
| Niben101Scf03860 | 490414-491489 | 3 |
| Niben101Scf03860 | 490415-491529 | 3 |
| Niben101Scf03860 | 490416-491329 | 3 |
| Niben101Scf03860 | 490418-492149 | 3 |
| Niben101Scf03860 | 490419-491597 | 3 |
| Niben101Scf03860 | 490421-491336 | 3 |
| Niben101Scf03860 | 490422-491335 | 3 |
| Niben101Scf03860 | 490423-491349 | 3 |
| Niben101Scf03860 | 490628-492410 | 3 |
| Niben101Scf03860 | 490658-492335 | 3 |
| Niben101Scf03860 | 490662-492339 | 3 |
| Niben101Scf03860 | 490688-491661 | 3 |
| Niben101Scf03860 | 490688-491773 | 3 |
| Niben101Scf03860 | 490694-491085 | 3 |
| Niben101Scf03860 | 490699-491090 | 3 |
| Niben101Scf03860 | 490703-491992 | 3 |
| Niben101Scf03860 | 490710-491999 | 3 |
| Niben101Scf03860 | 490730-492862 | 3 |
| Niben101Scf03860 | 490734-492866 | 3 |
| Niben101Scf03860 | 490780-492647 | 3 |
| Niben101Scf03860 | 490785-492652 | 3 |
| Niben101Scf03860 | 490788-492769 | 3 |
| Niben101Scf03860 | 490790-491669 | 3 |
| Niben101Scf03860 | 490790-492771 | 3 |
| Niben101Scf03860 | 490952-499073 | 3 |
| Niben101Scf03964 | 437318-438158 | 3 |
| Niben101Scf03985 | 562126-564936 | 3 |
| Niben101Scf04313 | 111505-112011 | 3 |
| Niben101Scf04436 | 1436949-1438047 | 3 |
| Niben101Scf04487 | 205893-206598 | 3 |
| Niben101Scf04727 | 332606-333161 | 3 |
| Niben101Scf05078 | 424257-425942 | 3 |
| Niben101Scf05245 | 134574-135357 | 3 |
| Niben101Scf06819 | 198723-200275 | 3 |
| Niben101Scf06819 | 198727-200279 | 3 |
| Niben101Scf07005 | 94148-98362 | 3 |
| Niben101Scf07441 | 132124-132797 | 3 |
| Niben101Scf07441 | 132125-132798 | 3 |
| Niben101Scf07441 | 132383-133285 | 3 |
| Niben101Scf07441 | 132388-133290 | 3 |
| Niben101Scf07895 | 145360-146325 | 3 |
| Niben101Scf07895 | 145363-146328 | 3 |
| Niben101Scf07895 | 145382-146377 | 3 |
| Niben101Scf07895 | 145385-146380 | 3 |
| Niben101Scf07933 | 222213-223135 | 3 |
| Niben101Scf07933 | 222219-223141 | 3 |
| Niben101Scf08108 | 45212-46575 | 3 |
| Niben101Scf08108 | 45216-46579 | 3 |
| Niben101Scf08519 | 3236-12168 | 3 |
| Niben101Scf08519 | 3242-12174 | 3 |
| Niben101Scf08566 | 444659-445722 | 3 |
| Niben101Scf08566 | 444661-445724 | 3 |
| Niben101Scf09355 | 61670-68250 | 3 |
| Niben101Scf09355 | 61672-68252 | 3 |
| Niben101Scf10283 | 75224-76839 | 3 |
| Niben101Scf10465 | 432283-433538 | 3 |
| Niben101Scf10579 | 210435-211153 | 3 |
| Niben101Scf10579 | 210439-211157 | 3 |
| Niben101Scf11653 | 79186-79835 | 3 |
| Niben101Scf11653 | 79192-79841 | 3 |
| Niben101Scf11778 | 88864-90228 | 3 |
| Niben101Scf12084 | 89253-93305 | 3 |
| Niben101Scf12084 | 89260-93312 | 3 |
| Niben101Scf12084 | 89357-91765 | 3 |
| Niben101Scf12084 | 89436-91570 | 3 |
| Niben101Scf12084 | 89490-93320 | 3 |
| Niben101Scf12084 | 89497-93198 | 3 |
| Niben101Scf12084 | 89499-93200 | 3 |
| Niben101Scf12084 | 89504-93204 | 3 |
| Niben101Scf12084 | 89505-93324 | 3 |
| Niben101Scf12084 | 89509-93328 | 3 |
| Niben101Scf12084 | 89510-93328 | 3 |
| Niben101Scf12084 | 89517-93335 | 3 |
| Niben101Scf12084 | 89525-93270 | 3 |
| Niben101Scf12084 | 89526-93271 | 3 |
| Niben101Scf12084 | 89535-93349 | 3 |
| Niben101Scf12084 | 89543-93261 | 3 |
| Niben101Scf12084 | 89564-93365 | 3 |
| Niben101Scf12084 | 89575-93462 | 3 |
| Niben101Scf12084 | 89577-93464 | 3 |
| Niben101Scf12084 | 89603-92033 | 3 |
| Niben101Scf12084 | 89607-93376 | 3 |
| Niben101Scf12084 | 89616-92706 | 3 |
| Niben101Scf12084 | 89629-93018 | 3 |
| Niben101Scf12084 | 89663-91770 | 3 |
| Niben101Scf12084 | 89701-92801 | 3 |
| Niben101Scf12084 | 89708-92808 | 3 |
| Niben101Scf12084 | 89713-92016 | 3 |
| Niben101Scf12084 | 89746-92599 | 3 |
| Niben101Scf12084 | 89748-92541 | 3 |
| Niben101Scf12084 | 89755-92548 | 3 |
| Niben101Scf12084 | 89766-92459 | 3 |
| Niben101Scf12084 | 89877-92456 | 3 |
| Niben101Scf12084 | 89880-92459 | 3 |
| Niben101Scf12084 | 90107-92457 | 3 |
| Niben101Scf12084 | 90110-92460 | 3 |
| Niben101Scf12084 | 90116-92466 | 3 |
| Niben101Scf12084 | 90152-93402 | 3 |
| Niben101Scf12084 | 90182-93420 | 3 |
| Niben101Scf12084 | 90183-93412 | 3 |
| Niben101Scf12084 | 90188-93417 | 3 |
| Niben101Scf12084 | 90189-93427 | 3 |
| Niben101Scf12084 | 90523-91774 | 3 |
| Niben101Scf12084 | 90527-91778 | 3 |
| Niben101Scf12084 | 90549-91482 | 3 |
| Niben101Scf12084 | 90554-93384 | 3 |
| Niben101Scf12084 | 90562-93392 | 3 |
| Niben101Scf12084 | 90693-93306 | 3 |
| Niben101Scf12084 | 90719-91552 | 3 |
| Niben101Scf12084 | 90722-91555 | 3 |
| Niben101Scf12084 | 90769-92866 | 3 |
| Niben101Scf12084 | 90795-91895 | 3 |
| Niben101Scf12084 | 90798-91898 | 3 |
| Niben101Scf12084 | 90813-93380 | 3 |
| Niben101Scf12084 | 90835-92601 | 3 |
| Niben101Scf12084 | 90851-91601 | 3 |
| Niben101Scf12084 | 90859-91609 | 3 |
| Niben101Scf12084 | 90862-93420 | 3 |
| Niben101Scf12084 | 90916-92757 | 3 |
| Niben101Scf12084 | 90961-92738 | 3 |
| Niben101Scf12084 | 90965-92742 | 3 |
| Niben101Scf12084 | 91061-93416 | 3 |
| Niben101Scf12084 | 91140-92877 | 3 |
| Niben101Scf12084 | 91140-93366 | 3 |
| Niben101Scf12084 | 91147-92884 | 3 |
| Niben101Scf12084 | 91215-93340 | 3 |
| Niben101Scf12084 | 91237-93027 | 3 |
| Niben101Scf12084 | 91242-93032 | 3 |
| Niben101Scf12084 | 91243-93296 | 3 |
| Niben101Scf12084 | 91249-92952 | 3 |
| Niben101Scf12084 | 91253-92956 | 3 |
| Niben101Scf12084 | 91254-93338 | 3 |
| Niben101Scf12084 | 91272-93418 | 3 |
| Niben101Scf12084 | 91276-93422 | 3 |
| Niben101Scf12084 | 91307-92971 | 3 |
| Niben101Scf12084 | 91308-92989 | 3 |
| Niben101Scf12084 | 91340-93457 | 3 |
| Niben101Scf12084 | 91360-91785 | 3 |
| Niben101Scf12868 | 64227-65471 | 3 |
| Niben101Scf14144 | 48486-52145 | 3 |
| Niben101Scf15187 | 11089-12316 | 3 |
| Niben101Scf15533 | 6219-6917 | 3 |
| Niben101Scf15533 | 6221-6919 | 3 |
| Niben101Scf16974 | 63053-81611 | 3 |
| Niben101Scf16974 | 79676-81498 | 3 |
| Niben101Scf16974 | 79679-81501 | 3 |
| Niben101Scf26545 | 40842-42354 | 3 |
| Niben101Scf26545 | 40847-42359 | 3 |
| Niben101Ctg16374 | 6223-7168 | 2 |
| Niben101Ctg16374 | 6228-7173 | 2 |
| Niben101Scf00056 | 364085-364832 | 2 |
| Niben101Scf00278 | 989043-991016 | 2 |
| Niben101Scf00278 | 989050-991023 | 2 |
| Niben101Scf00278 | 989073-991043 | 2 |
| Niben101Scf00397 | 289564-290102 | 2 |
| Niben101Scf00397 | 289571-290109 | 2 |
| Niben101Scf00439 | 1070938-1071550 | 2 |
| Niben101Scf00539 | 277987-278412 | 2 |
| Niben101Scf00577 | 1132837-1134073 | 2 |
| Niben101Scf00577 | 1132844-1134080 | 2 |
| Niben101Scf00635 | 47397-48015 | 2 |
| Niben101Scf00635 | 47399-48017 | 2 |
| Niben101Scf00705 | 31791-32463 | 2 |
| Niben101Scf00705 | 31799-32471 | 2 |
| Niben101Scf00918 | 476606-478945 | 2 |
| Niben101Scf01409 | 1355188-1356616 | 2 |
| Niben101Scf01505 | 245015-251324 | 2 |
| Niben101Scf01505 | 245018-251327 | 2 |
| Niben101Scf01623 | 397179-398374 | 2 |
| Niben101Scf01750 | 959383-959528 | 2 |
| Niben101Scf01750 | 959385-959530 | 2 |
| Niben101Scf01983 | 938547-939927 | 2 |
| Niben101Scf01986 | 213124-213713 | 2 |
| Niben101Scf02073 | 207142-209335 | 2 |
| Niben101Scf02159 | 119928-120846 | 2 |
| Niben101Scf02182 | 140331-141603 | 2 |
| Niben101Scf02262 | 171392-172180 | 2 |
| Niben101Scf02268 | 544364-549039 | 2 |
| Niben101Scf02268 | 544373-549048 | 2 |
| Niben101Scf02279 | 565691-566717 | 2 |
| Niben101Scf02279 | 565692-566718 | 2 |
| Niben101Scf02353 | 135850-136518 | 2 |
| Niben101Scf02535 | 287786-288481 | 2 |
| Niben101Scf02627 | 113073-113996 | 2 |
| Niben101Scf02792 | 206993-208722 | 2 |
| Niben101Scf02805 | 751330-753028 | 2 |
| Niben101Scf02985 | 3953-5812 | 2 |
| Niben101Scf02985 | 4511-5796 | 2 |
| Niben101Scf02985 | 5315-6114 | 2 |
| Niben101Scf03173 | 74525-75348 | 2 |
| Niben101Scf03173 | 74531-75354 | 2 |
| Niben101Scf03253 | 302102-306782 | 2 |
| Niben101Scf03371 | 339655-339986 | 2 |
| Niben101Scf03376 | 499051-499916 | 2 |
| Niben101Scf03404 | 42864-49379 | 2 |
| Niben101Scf03450 | 159014-159544 | 2 |
| Niben101Scf03592 | 93281-94050 | 2 |
| Niben101Scf03788 | 64700-65350 | 2 |
| Niben101Scf03860 | 485426-495815 | 2 |
| Niben101Scf03860 | 485445-494024 | 2 |
| Niben101Scf03860 | 485636-499341 | 2 |
| Niben101Scf03860 | 485661-493767 | 2 |
| Niben101Scf03860 | 485682-497113 | 2 |
| Niben101Scf03860 | 485718-493930 | 2 |
| Niben101Scf03860 | 485725-493937 | 2 |
| Niben101Scf03860 | 485780-493305 | 2 |
| Niben101Scf03860 | 485955-493375 | 2 |
| Niben101Scf03860 | 485957-498670 | 2 |
| Niben101Scf03860 | 485959-493379 | 2 |
| Niben101Scf03860 | 486351-494880 | 2 |
| Niben101Scf03860 | 486356-494885 | 2 |
| Niben101Scf03860 | 486370-497694 | 2 |
| Niben101Scf03860 | 486478-496090 | 2 |
| Niben101Scf03860 | 486507-494450 | 2 |
| Niben101Scf03860 | 486590-499692 | 2 |
| Niben101Scf03860 | 486647-498346 | 2 |
| Niben101Scf03860 | 486666-496035 | 2 |
| Niben101Scf03860 | 486847-494348 | 2 |
| Niben101Scf03860 | 486857-494985 | 2 |
| Niben101Scf03860 | 486862-494990 | 2 |
| Niben101Scf03860 | 486960-497814 | 2 |
| Niben101Scf03860 | 486998-497183 | 2 |
| Niben101Scf03860 | 487174-494684 | 2 |
| Niben101Scf03860 | 487299-496631 | 2 |
| Niben101Scf03860 | 487456-498135 | 2 |
| Niben101Scf03860 | 487575-498557 | 2 |
| Niben101Scf03860 | 487582-498564 | 2 |
| Niben101Scf03860 | 487582-499069 | 2 |
| Niben101Scf03860 | 487600-497338 | 2 |
| Niben101Scf03860 | 487628-496922 | 2 |
| Niben101Scf03860 | 487672-498073 | 2 |
| Niben101Scf03860 | 487773-498316 | 2 |
| Niben101Scf03860 | 487781-498324 | 2 |
| Niben101Scf03860 | 487782-497835 | 2 |
| Niben101Scf03860 | 487877-496905 | 2 |
| Niben101Scf03860 | 488165-498352 | 2 |
| Niben101Scf03860 | 488201-497900 | 2 |
| Niben101Scf03860 | 488208-497907 | 2 |
| Niben101Scf03860 | 488262-497183 | 2 |
| Niben101Scf03860 | 488270-497191 | 2 |
| Niben101Scf03860 | 488380-498964 | 2 |
| Niben101Scf03860 | 488471-499808 | 2 |
| Niben101Scf03860 | 488635-499530 | 2 |
| Niben101Scf03860 | 488930-490221 | 2 |
| Niben101Scf03860 | 488934-490225 | 2 |
| Niben101Scf03860 | 488937-498118 | 2 |
| Niben101Scf03860 | 488945-498126 | 2 |
| Niben101Scf03860 | 488962-490421 | 2 |
| Niben101Scf03860 | 489034-490283 | 2 |
| Niben101Scf03860 | 489041-490225 | 2 |
| Niben101Scf03860 | 489123-490244 | 2 |
| Niben101Scf03860 | 489128-490390 | 2 |
| Niben101Scf03860 | 489132-490394 | 2 |
| Niben101Scf03860 | 489139-490401 | 2 |
| Niben101Scf03860 | 489174-497927 | 2 |
| Niben101Scf03860 | 489179-497932 | 2 |
| Niben101Scf03860 | 489189-497470 | 2 |
| Niben101Scf03860 | 489205-490229 | 2 |
| Niben101Scf03860 | 489208-490232 | 2 |
| Niben101Scf03860 | 489225-498078 | 2 |
| Niben101Scf03860 | 489236-497183 | 2 |
| Niben101Scf03860 | 489236-499717 | 2 |
| Niben101Scf03860 | 489322-490515 | 2 |
| Niben101Scf03860 | 489323-498118 | 2 |
| Niben101Scf03860 | 489331-498126 | 2 |
| Niben101Scf03860 | 489332-490381 | 2 |
| Niben101Scf03860 | 489340-490342 | 2 |
| Niben101Scf03860 | 489341-490340 | 2 |
| Niben101Scf03860 | 489351-490353 | 2 |
| Niben101Scf03860 | 489354-499494 | 2 |
| Niben101Scf03860 | 489358-490360 | 2 |
| Niben101Scf03860 | 489358-490511 | 2 |
| Niben101Scf03860 | 489361-499501 | 2 |
| Niben101Scf03860 | 489457-490347 | 2 |
| Niben101Scf03860 | 489460-490350 | 2 |
| Niben101Scf03860 | 489460-498566 | 2 |
| Niben101Scf03860 | 489461-498567 | 2 |
| Niben101Scf03860 | 489470-490409 | 2 |
| Niben101Scf03860 | 489477-490416 | 2 |
| Niben101Scf03860 | 489483-498194 | 2 |
| Niben101Scf03860 | 489484-498151 | 2 |
| Niben101Scf03860 | 489484-498195 | 2 |
| Niben101Scf03860 | 489486-498153 | 2 |
| Niben101Scf03860 | 489515-490659 | 2 |
| Niben101Scf03860 | 489541-497925 | 2 |
| Niben101Scf03860 | 489552-490808 | 2 |
| Niben101Scf03860 | 489562-490444 | 2 |
| Niben101Scf03860 | 489580-491764 | 2 |
| Niben101Scf03860 | 489613-498101 | 2 |
| Niben101Scf03860 | 489615-498103 | 2 |
| Niben101Scf03860 | 489629-491858 | 2 |
| Niben101Scf03860 | 489640-491869 | 2 |
| Niben101Scf03860 | 489642-491870 | 2 |
| Niben101Scf03860 | 489676-498716 | 2 |
| Niben101Scf03860 | 489683-498723 | 2 |
| Niben101Scf03860 | 489714-497593 | 2 |
| Niben101Scf03860 | 489735-498377 | 2 |
| Niben101Scf03860 | 489777-491957 | 2 |
| Niben101Scf03860 | 489848-492075 | 2 |
| Niben101Scf03860 | 489853-492080 | 2 |
| Niben101Scf03860 | 489873-498219 | 2 |
| Niben101Scf03860 | 489890-497768 | 2 |
| Niben101Scf03860 | 489891-497769 | 2 |
| Niben101Scf03860 | 489913-492213 | 2 |
| Niben101Scf03860 | 489913-497772 | 2 |
| Niben101Scf03860 | 489936-492088 | 2 |
| Niben101Scf03860 | 489947-498828 | 2 |
| Niben101Scf03860 | 489965-492148 | 2 |
| Niben101Scf03860 | 489971-497840 | 2 |
| Niben101Scf03860 | 489998-497579 | 2 |
| Niben101Scf03860 | 489999-497580 | 2 |
| Niben101Scf03860 | 490030-497972 | 2 |
| Niben101Scf03860 | 490030-499466 | 2 |
| Niben101Scf03860 | 490048-492263 | 2 |
| Niben101Scf03860 | 490050-492266 | 2 |
| Niben101Scf03860 | 490056-492271 | 2 |
| Niben101Scf03860 | 490057-492272 | 2 |
| Niben101Scf03860 | 490057-492273 | 2 |
| Niben101Scf03860 | 490059-492274 | 2 |
| Niben101Scf03860 | 490070-490847 | 2 |
| Niben101Scf03860 | 490070-498486 | 2 |
| Niben101Scf03860 | 490075-498491 | 2 |
| Niben101Scf03860 | 490075-499811 | 2 |
| Niben101Scf03860 | 490154-497849 | 2 |
| Niben101Scf03860 | 490162-497857 | 2 |
| Niben101Scf03860 | 490308-498486 | 2 |
| Niben101Scf03860 | 490367-498301 | 2 |
| Niben101Scf03860 | 490381-497975 | 2 |
| Niben101Scf03860 | 490385-497964 | 2 |
| Niben101Scf03860 | 490386-497980 | 2 |
| Niben101Scf03860 | 490390-499209 | 2 |
| Niben101Scf03860 | 490395-499214 | 2 |
| Niben101Scf03860 | 490407-497789 | 2 |
| Niben101Scf03860 | 490459-492638 | 2 |
| Niben101Scf03860 | 490477-492725 | 2 |
| Niben101Scf03860 | 490659-499948 | 2 |
| Niben101Scf03860 | 490671-498649 | 2 |
| Niben101Scf03860 | 490680-499732 | 2 |
| Niben101Scf03860 | 490711-492953 | 2 |
| Niben101Scf03860 | 490712-490879 | 2 |
| Niben101Scf03860 | 490716-490883 | 2 |
| Niben101Scf03860 | 490725-499331 | 2 |
| Niben101Scf03860 | 490808-498957 | 2 |
| Niben101Scf03860 | 491109-499098 | 2 |
| Niben101Scf03860 | 491196-500187 | 2 |
| Niben101Scf03860 | 491262-500351 | 2 |
| Niben101Scf03860 | 491263-500352 | 2 |
| Niben101Scf03860 | 491293-499818 | 2 |
| Niben101Scf03860 | 491305-499830 | 2 |
| Niben101Scf03964 | 437454-437978 | 2 |
| Niben101Scf03985 | 562125-564935 | 2 |
| Niben101Scf04285 | 144825-145511 | 2 |
| Niben101Scf04285 | 144831-145517 | 2 |
| Niben101Scf04285 | 147012-148145 | 2 |
| Niben101Scf04285 | 147017-148150 | 2 |
| Niben101Scf04664 | 167427-167824 | 2 |
| Niben101Scf04664 | 167428-167825 | 2 |
| Niben101Scf04745 | 545330-547711 | 2 |
| Niben101Scf04860 | 134780-135577 | 2 |
| Niben101Scf04860 | 134787-135584 | 2 |
| Niben101Scf04943 | 210767-213183 | 2 |
| Niben101Scf04943 | 210771-213187 | 2 |
| Niben101Scf05035 | 567295-570006 | 2 |
| Niben101Scf05035 | 567299-570010 | 2 |
| Niben101Scf05490 | 70322-70992 | 2 |
| Niben101Scf05490 | 70323-70993 | 2 |
| Niben101Scf06347 | 464621-468399 | 2 |
| Niben101Scf06926 | 673583-673999 | 2 |
| Niben101Scf07662 | 273731-275084 | 2 |
| Niben101Scf07662 | 273737-275090 | 2 |
| Niben101Scf07662 | 273916-275052 | 2 |
| Niben101Scf07662 | 274349-275879 | 2 |
| Niben101Scf07662 | 274769-276425 | 2 |
| Niben101Scf07662 | 274771-276427 | 2 |
| Niben101Scf07662 | 274920-275445 | 2 |
| Niben101Scf07662 | 274923-275448 | 2 |
| Niben101Scf07662 | 275021-275458 | 2 |
| Niben101Scf07798 | 374026-375237 | 2 |
| Niben101Scf07812 | 73142-74270 | 2 |
| Niben101Scf07812 | 73146-74274 | 2 |
| Niben101Scf08319 | 127-958 | 2 |
| Niben101Scf08597 | 267730-268402 | 2 |
| Niben101Scf08899 | 86656-87928 | 2 |
| Niben101Scf08899 | 86664-87936 | 2 |
| Niben101Scf09260 | 119209-122130 | 2 |
| Niben101Scf09782 | 8233-9228 | 2 |
| Niben101Scf10065 | 14186-14863 | 2 |
| Niben101Scf10065 | 14188-14865 | 2 |
| Niben101Scf10369 | 232408-233144 | 2 |
| Niben101Scf10519 | 210482-212368 | 2 |
| Niben101Scf10519 | 210485-212371 | 2 |
| Niben101Scf11130 | 9737-11013 | 2 |
| Niben101Scf11653 | 78072-79174 | 2 |
| Niben101Scf11653 | 78165-79438 | 2 |
| Niben101Scf11653 | 78169-79442 | 2 |
| Niben101Scf12084 | 90105-91419 | 2 |
| Niben101Scf12966 | 43648-44145 | 2 |
| Niben101Scf12966 | 43653-44150 | 2 |
| Niben101Scf13146 | 129335-130593 | 2 |
| Niben101Scf13146 | 129337-130595 | 2 |
| Niben101Scf13180 | 307117-311172 | 2 |
| Niben101Scf13180 | 307121-311176 | 2 |
| Niben101Scf13540 | 499012-499421 | 2 |
| Niben101Scf13540 | 499077-499540 | 2 |
| Niben101Scf13540 | 499083-499546 | 2 |
| Niben101Scf18902 | 15147-15754 | 2 |
| Niben101Scf18902 | 15150-15757 | 2 |
| Niben101Scf28696 | 2757-4133 | 2 |
| Niben101Scf35487 | 22484-23272 | 2 |
| Niben101Scf00011 | 27862-28492 | 1 |
| Niben101Scf00125 | 210501-211245 | 1 |
| Niben101Scf00138 | 131102-131495 | 1 |
| Niben101Scf00138 | 131104-131497 | 1 |
| Niben101Scf00148 | 131934-132829 | 1 |
| Niben101Scf00254 | 550882-552367 | 1 |
| Niben101Scf00262 | 162014-163389 | 1 |
| Niben101Scf00262 | 162018-163393 | 1 |
| Niben101Scf00360 | 165851-167571 | 1 |
| Niben101Scf00360 | 165854-167575 | 1 |
| Niben101Scf00360 | 165867-167850 | 1 |
| Niben101Scf00360 | 165989-168217 | 1 |
| Niben101Scf00360 | 165993-168221 | 1 |
| Niben101Scf00360 | 166163-167329 | 1 |
| Niben101Scf00360 | 166166-167332 | 1 |
| Niben101Scf00381 | 644355-644728 | 1 |
| Niben101Scf00381 | 644357-644730 | 1 |
| Niben101Scf00481 | 207571-210587 | 1 |
| Niben101Scf00501 | 271769-272303 | 1 |
| Niben101Scf00501 | 271773-272307 | 1 |
| Niben101Scf00503 | 152555-154918 | 1 |
| Niben101Scf00503 | 152557-154920 | 1 |
| Niben101Scf00525 | 558739-559450 | 1 |
| Niben101Scf00539 | 278466-279605 | 1 |
| Niben101Scf00650 | 231292-232038 | 1 |
| Niben101Scf00650 | 231293-232039 | 1 |
| Niben101Scf00735 | 623055-623513 | 1 |
| Niben101Scf00797 | 1851078-1851609 | 1 |
| Niben101Scf00797 | 1851082-1851613 | 1 |
| Niben101Scf00801 | 3370-4094 | 1 |
| Niben101Scf00819 | 155545-156213 | 1 |
| Niben101Scf00819 | 155555-156223 | 1 |
| Niben101Scf00870 | 512067-512529 | 1 |
| Niben101Scf00875 | 1219354-1220099 | 1 |
| Niben101Scf00927 | 17025-17609 | 1 |
| Niben101Scf00927 | 17031-17615 | 1 |
| Niben101Scf00953 | 75720-76229 | 1 |
| Niben101Scf01063 | 429105-430206 | 1 |
| Niben101Scf01063 | 429113-430214 | 1 |
| Niben101Scf01068 | 564561-565294 | 1 |
| Niben101Scf01068 | 564564-565297 | 1 |
| Niben101Scf01068 | 564635-566561 | 1 |
| Niben101Scf01068 | 564680-566568 | 1 |
| Niben101Scf01068 | 564691-566579 | 1 |
| Niben101Scf01068 | 564710-566484 | 1 |
| Niben101Scf01068 | 564714-566488 | 1 |
| Niben101Scf01068 | 564750-564974 | 1 |
| Niben101Scf01068 | 564755-564979 | 1 |
| Niben101Scf01068 | 564771-566520 | 1 |
| Niben101Scf01068 | 564776-566525 | 1 |
| Niben101Scf01068 | 564786-566637 | 1 |
| Niben101Scf01090 | 658318-658678 | 1 |
| Niben101Scf01146 | 1365639-1366099 | 1 |
| Niben101Scf01146 | 1365643-1366103 | 1 |
| Niben101Scf01150 | 387434-387723 | 1 |
| Niben101Scf01150 | 387442-388001 | 1 |
| Niben101Scf01150 | 387446-388005 | 1 |
| Niben101Scf01150 | 387460-387698 | 1 |
| Niben101Scf01150 | 387469-387707 | 1 |
| Niben101Scf01150 | 387710-388183 | 1 |
| Niben101Scf01222 | 69976-70595 | 1 |
| Niben101Scf01329 | 6139-7174 | 1 |
| Niben101Scf01329 | 6143-7178 | 1 |
| Niben101Scf01329 | 6354-7488 | 1 |
| Niben101Scf01329 | 6683-7004 | 1 |
| Niben101Scf01329 | 6695-7016 | 1 |
| Niben101Scf01385 | 718653-720380 | 1 |
| Niben101Scf01385 | 718660-720387 | 1 |
| Niben101Scf01412 | 140231-140687 | 1 |
| Niben101Scf01433 | 941947-942479 | 1 |
| Niben101Scf01463 | 880603-881185 | 1 |
| Niben101Scf01494 | 961757-962621 | 1 |
| Niben101Scf01498 | 720758-721501 | 1 |
| Niben101Scf01517 | 793005-793812 | 1 |
| Niben101Scf01555 | 484992-485767 | 1 |
| Niben101Scf01587 | 166473-167331 | 1 |
| Niben101Scf01634 | 440079-442895 | 1 |
| Niben101Scf01634 | 440135-442951 | 1 |
| Niben101Scf01701 | 83468-83999 | 1 |
| Niben101Scf01777 | 395668-396290 | 1 |
| Niben101Scf01857 | 152549-152921 | 1 |
| Niben101Scf01857 | 152555-152927 | 1 |
| Niben101Scf01956 | 259683-260322 | 1 |
| Niben101Scf01956 | 259685-260324 | 1 |
| Niben101Scf01971 | 246760-247569 | 1 |
| Niben101Scf01971 | 246769-247578 | 1 |
| Niben101Scf02033 | 20720-21068 | 1 |
| Niben101Scf02114 | 505828-506740 | 1 |
| Niben101Scf02114 | 505837-506749 | 1 |
| Niben101Scf02155 | 54345-54511 | 1 |
| Niben101Scf02164 | 385788-386459 | 1 |
| Niben101Scf02164 | 385791-386462 | 1 |
| Niben101Scf02211 | 311776-312009 | 1 |
| Niben101Scf02272 | 264040-265153 | 1 |
| Niben101Scf02272 | 264046-265159 | 1 |
| Niben101Scf02293 | 288846-289124 | 1 |
| Niben101Scf02293 | 288849-289127 | 1 |
| Niben101Scf02430 | 330575-331447 | 1 |
| Niben101Scf02430 | 330668-331699 | 1 |
| Niben101Scf02430 | 330765-331940 | 1 |
| Niben101Scf02430 | 330770-331945 | 1 |
| Niben101Scf02430 | 330777-331359 | 1 |
| Niben101Scf02471 | 50683-51530 | 1 |
| Niben101Scf02471 | 50688-51535 | 1 |
| Niben101Scf02497 | 253295-255977 | 1 |
| Niben101Scf02525 | 439511-445581 | 1 |
| Niben101Scf02626 | 662302-662517 | 1 |
| Niben101Scf02626 | 662305-662520 | 1 |
| Niben101Scf02812 | 236654-237857 | 1 |
| Niben101Scf02869 | 204276-205003 | 1 |
| Niben101Scf02985 | 3957-5817 | 1 |
| Niben101Scf02985 | 3963-5822 | 1 |
| Niben101Scf02985 | 4583-5265 | 1 |
| Niben101Scf02985 | 7630-8330 | 1 |
| Niben101Scf02985 | 7931-9246 | 1 |
| Niben101Scf02985 | 7941-9256 | 1 |
| Niben101Scf02985 | 8704-9363 | 1 |
| Niben101Scf03169 | 662072-662417 | 1 |
| Niben101Scf03169 | 662077-662422 | 1 |
| Niben101Scf03184 | 726572-726933 | 1 |
| Niben101Scf03307 | 151291-152137 | 1 |
| Niben101Scf03307 | 151294-152140 | 1 |
| Niben101Scf03428 | 115014-116514 | 1 |
| Niben101Scf03450 | 159018-159548 | 1 |
| Niben101Scf03468 | 324984-326370 | 1 |
| Niben101Scf03479 | 356247-356753 | 1 |
| Niben101Scf03492 | 122867-123569 | 1 |
| Niben101Scf03503 | 224250-226891 | 1 |
| Niben101Scf03503 | 224255-226896 | 1 |
| Niben101Scf03573 | 129596-131551 | 1 |
| Niben101Scf03573 | 129601-131556 | 1 |
| Niben101Scf03584 | 289290-289808 | 1 |
| Niben101Scf03595 | 1896418-1897413 | 1 |
| Niben101Scf03595 | 1896422-1897417 | 1 |
| Niben101Scf03595 | 453785-454313 | 1 |
| Niben101Scf03595 | 453789-454317 | 1 |
| Niben101Scf03839 | 173867-174888 | 1 |
| Niben101Scf03839 | 173868-174889 | 1 |
| Niben101Scf03850 | 223271-223781 | 1 |
| Niben101Scf03850 | 223274-223784 | 1 |
| Niben101Scf03860 | 483543-486419 | 1 |
| Niben101Scf03860 | 483547-486423 | 1 |
| Niben101Scf03860 | 483638-491974 | 1 |
| Niben101Scf03860 | 483644-491980 | 1 |
| Niben101Scf03860 | 483646-487171 | 1 |
| Niben101Scf03860 | 483678-490790 | 1 |
| Niben101Scf03860 | 483691-486995 | 1 |
| Niben101Scf03860 | 483705-486999 | 1 |
| Niben101Scf03860 | 483736-486837 | 1 |
| Niben101Scf03860 | 483757-491095 | 1 |
| Niben101Scf03860 | 483761-486423 | 1 |
| Niben101Scf03860 | 483764-486999 | 1 |
| Niben101Scf03860 | 483765-487000 | 1 |
| Niben101Scf03860 | 483766-486428 | 1 |
| Niben101Scf03860 | 483784-487064 | 1 |
| Niben101Scf03860 | 483916-487341 | 1 |
| Niben101Scf03860 | 483923-486740 | 1 |
| Niben101Scf03860 | 483923-492328 | 1 |
| Niben101Scf03860 | 483930-486747 | 1 |
| Niben101Scf03860 | 483948-492319 | 1 |
| Niben101Scf03860 | 483959-491713 | 1 |
| Niben101Scf03860 | 483960-491714 | 1 |
| Niben101Scf03860 | 484113-491848 | 1 |
| Niben101Scf03860 | 484146-487533 | 1 |
| Niben101Scf03860 | 484178-492055 | 1 |
| Niben101Scf03860 | 484181-492058 | 1 |
| Niben101Scf03860 | 484192-492067 | 1 |
| Niben101Scf03860 | 484193-492068 | 1 |
| Niben101Scf03860 | 484232-491507 | 1 |
| Niben101Scf03860 | 484237-491512 | 1 |
| Niben101Scf03860 | 484257-487699 | 1 |
| Niben101Scf03860 | 484264-487706 | 1 |
| Niben101Scf03860 | 484286-492752 | 1 |
| Niben101Scf03860 | 484288-492031 | 1 |
| Niben101Scf03860 | 484293-492036 | 1 |
| Niben101Scf03860 | 484311-493128 | 1 |
| Niben101Scf03860 | 484329-493035 | 1 |
| Niben101Scf03860 | 484333-487081 | 1 |
| Niben101Scf03860 | 484347-487169 | 1 |
| Niben101Scf03860 | 484347-487621 | 1 |
| Niben101Scf03860 | 484371-491622 | 1 |
| Niben101Scf03860 | 484377-491628 | 1 |
| Niben101Scf03860 | 484377-492036 | 1 |
| Niben101Scf03860 | 484398-487868 | 1 |
| Niben101Scf03860 | 484402-492237 | 1 |
| Niben101Scf03860 | 484406-492241 | 1 |
| Niben101Scf03860 | 484413-487582 | 1 |
| Niben101Scf03860 | 484419-493107 | 1 |
| Niben101Scf03860 | 484420-487647 | 1 |
| Niben101Scf03860 | 484420-487959 | 1 |
| Niben101Scf03860 | 484426-487653 | 1 |
| Niben101Scf03860 | 484534-487860 | 1 |
| Niben101Scf03860 | 484554-488206 | 1 |
| Niben101Scf03860 | 484561-486830 | 1 |
| Niben101Scf03860 | 484572-488327 | 1 |
| Niben101Scf03860 | 484575-488142 | 1 |
| Niben101Scf03860 | 484589-487882 | 1 |
| Niben101Scf03860 | 484593-487886 | 1 |
| Niben101Scf03860 | 484600-491983 | 1 |
| Niben101Scf03860 | 484604-491987 | 1 |
| Niben101Scf03860 | 484671-488410 | 1 |
| Niben101Scf03860 | 484691-493172 | 1 |
| Niben101Scf03860 | 484695-487944 | 1 |
| Niben101Scf03860 | 484701-488060 | 1 |
| Niben101Scf03860 | 484703-488062 | 1 |
| Niben101Scf03860 | 484734-488397 | 1 |
| Niben101Scf03860 | 484757-492760 | 1 |
| Niben101Scf03860 | 484819-486726 | 1 |
| Niben101Scf03860 | 484850-487675 | 1 |
| Niben101Scf03860 | 484853-487678 | 1 |
| Niben101Scf03860 | 484864-487700 | 1 |
| Niben101Scf03860 | 484915-486764 | 1 |
| Niben101Scf03860 | 484929-486777 | 1 |
| Niben101Scf03860 | 484937-486785 | 1 |
| Niben101Scf03860 | 484951-486894 | 1 |
| Niben101Scf03860 | 484955-486898 | 1 |
| Niben101Scf03860 | 484972-488749 | 1 |
| Niben101Scf03860 | 484990-487746 | 1 |
| Niben101Scf03860 | 485056-492292 | 1 |
| Niben101Scf03860 | 485061-492297 | 1 |
| Niben101Scf03860 | 485074-486815 | 1 |
| Niben101Scf03860 | 485077-486818 | 1 |
| Niben101Scf03860 | 485093-487782 | 1 |
| Niben101Scf03860 | 485096-487785 | 1 |
| Niben101Scf03860 | 485136-488625 | 1 |
| Niben101Scf03860 | 485137-488726 | 1 |
| Niben101Scf03860 | 485139-488704 | 1 |
| Niben101Scf03860 | 485139-488904 | 1 |
| Niben101Scf03860 | 485143-488908 | 1 |
| Niben101Scf03860 | 485146-488711 | 1 |
| Niben101Scf03860 | 485186-492344 | 1 |
| Niben101Scf03860 | 485207-488924 | 1 |
| Niben101Scf03860 | 485218-487807 | 1 |
| Niben101Scf03860 | 485219-492720 | 1 |
| Niben101Scf03860 | 485224-488927 | 1 |
| Niben101Scf03860 | 485224-492725 | 1 |
| Niben101Scf03860 | 485267-487697 | 1 |
| Niben101Scf03860 | 485272-487702 | 1 |
| Niben101Scf03860 | 485283-488713 | 1 |
| Niben101Scf03860 | 485288-489136 | 1 |
| Niben101Scf03860 | 485306-487120 | 1 |
| Niben101Scf03860 | 485329-492555 | 1 |
| Niben101Scf03860 | 485353-487164 | 1 |
| Niben101Scf03860 | 485363-487174 | 1 |
| Niben101Scf03860 | 485393-489012 | 1 |
| Niben101Scf03860 | 485395-489014 | 1 |
| Niben101Scf03860 | 485405-486716 | 1 |
| Niben101Scf03860 | 485421-486694 | 1 |
| Niben101Scf03860 | 485427-486700 | 1 |
| Niben101Scf03860 | 485433-488470 | 1 |
| Niben101Scf03860 | 485447-489286 | 1 |
| Niben101Scf03860 | 485466-488660 | 1 |
| Niben101Scf03860 | 485475-488318 | 1 |
| Niben101Scf03860 | 485480-489021 | 1 |
| Niben101Scf03860 | 485493-487568 | 1 |
| Niben101Scf03860 | 485495-488591 | 1 |
| Niben101Scf03860 | 485495-489297 | 1 |
| Niben101Scf03860 | 485496-488593 | 1 |
| Niben101Scf03860 | 485497-487572 | 1 |
| Niben101Scf03860 | 485502-488599 | 1 |
| Niben101Scf03860 | 485524-488615 | 1 |
| Niben101Scf03860 | 485526-489042 | 1 |
| Niben101Scf03860 | 485528-489044 | 1 |
| Niben101Scf03860 | 485531-489317 | 1 |
| Niben101Scf03860 | 485540-486743 | 1 |
| Niben101Scf03860 | 485547-486750 | 1 |
| Niben101Scf03860 | 485547-486752 | 1 |
| Niben101Scf03860 | 485547-487341 | 1 |
| Niben101Scf03860 | 485547-488123 | 1 |
| Niben101Scf03860 | 485552-489223 | 1 |
| Niben101Scf03860 | 485558-489344 | 1 |
| Niben101Scf03860 | 485563-489349 | 1 |
| Niben101Scf03860 | 485568-487474 | 1 |
| Niben101Scf03860 | 485569-489092 | 1 |
| Niben101Scf03860 | 485570-487146 | 1 |
| Niben101Scf03860 | 485578-488764 | 1 |
| Niben101Scf03860 | 485580-488766 | 1 |
| Niben101Scf03860 | 485631-487666 | 1 |
| Niben101Scf03860 | 485634-486879 | 1 |
| Niben101Scf03860 | 485636-488537 | 1 |
| Niben101Scf03860 | 485638-486883 | 1 |
| Niben101Scf03860 | 485681-487027 | 1 |
| Niben101Scf03860 | 485686-487032 | 1 |
| Niben101Scf03860 | 485744-486786 | 1 |
| Niben101Scf03860 | 485780-489295 | 1 |
| Niben101Scf03860 | 485781-489296 | 1 |
| Niben101Scf03860 | 485790-487598 | 1 |
| Niben101Scf03860 | 485823-488036 | 1 |
| Niben101Scf03860 | 485832-488295 | 1 |
| Niben101Scf03860 | 485836-488299 | 1 |
| Niben101Scf03860 | 485840-489184 | 1 |
| Niben101Scf03860 | 485841-489615 | 1 |
| Niben101Scf03860 | 485846-489620 | 1 |
| Niben101Scf03860 | 485891-487750 | 1 |
| Niben101Scf03860 | 485895-488665 | 1 |
| Niben101Scf03860 | 485896-487755 | 1 |
| Niben101Scf03860 | 485897-488667 | 1 |
| Niben101Scf03860 | 485916-493224 | 1 |
| Niben101Scf03860 | 485917-488618 | 1 |
| Niben101Scf03860 | 485945-487053 | 1 |
| Niben101Scf03860 | 485945-488117 | 1 |
| Niben101Scf03860 | 485949-488121 | 1 |
| Niben101Scf03860 | 485961-488547 | 1 |
| Niben101Scf03860 | 485965-489268 | 1 |
| Niben101Scf03860 | 485970-489273 | 1 |
| Niben101Scf03860 | 485973-488390 | 1 |
| Niben101Scf03860 | 485983-487455 | 1 |
| Niben101Scf03860 | 485983-489043 | 1 |
| Niben101Scf03860 | 485991-488657 | 1 |
| Niben101Scf03860 | 486003-487797 | 1 |
| Niben101Scf03860 | 486005-487799 | 1 |
| Niben101Scf03860 | 486031-489668 | 1 |
| Niben101Scf03860 | 486034-489671 | 1 |
| Niben101Scf03860 | 486065-488109 | 1 |
| Niben101Scf03860 | 486078-488231 | 1 |
| Niben101Scf03860 | 486079-486768 | 1 |
| Niben101Scf03860 | 486155-489212 | 1 |
| Niben101Scf03860 | 486158-489215 | 1 |
| Niben101Scf03860 | 486170-489593 | 1 |
| Niben101Scf03860 | 486171-488482 | 1 |
| Niben101Scf03860 | 486177-489072 | 1 |
| Niben101Scf03860 | 486177-489600 | 1 |
| Niben101Scf03860 | 486190-489103 | 1 |
| Niben101Scf03860 | 486195-489108 | 1 |
| Niben101Scf03860 | 486242-487217 | 1 |
| Niben101Scf03860 | 486272-490077 | 1 |
| Niben101Scf03860 | 486273-489619 | 1 |
| Niben101Scf03860 | 486273-489889 | 1 |
| Niben101Scf03860 | 486293-488813 | 1 |
| Niben101Scf03860 | 486298-488818 | 1 |
| Niben101Scf03860 | 486321-489836 | 1 |
| Niben101Scf03860 | 486324-488693 | 1 |
| Niben101Scf03860 | 486351-489226 | 1 |
| Niben101Scf03860 | 486353-489596 | 1 |
| Niben101Scf03860 | 486360-489603 | 1 |
| Niben101Scf03860 | 486364-489282 | 1 |
| Niben101Scf03860 | 486369-489287 | 1 |
| Niben101Scf03860 | 486385-487155 | 1 |
| Niben101Scf03860 | 486386-488756 | 1 |
| Niben101Scf03860 | 486389-487159 | 1 |
| Niben101Scf03860 | 486393-489999 | 1 |
| Niben101Scf03860 | 486394-490057 | 1 |
| Niben101Scf03860 | 486451-493816 | 1 |
| Niben101Scf03860 | 486926-487575 | 1 |
| Niben101Scf03860 | 487421-494597 | 1 |
| Niben101Scf03860 | 487939-490206 | 1 |
| Niben101Scf03860 | 488362-490351 | 1 |
| Niben101Scf03860 | 488365-490358 | 1 |
| Niben101Scf03860 | 488366-490355 | 1 |
| Niben101Scf03860 | 488372-490365 | 1 |
| Niben101Scf03860 | 488379-490375 | 1 |
| Niben101Scf03860 | 488380-490376 | 1 |
| Niben101Scf03860 | 488381-490377 | 1 |
| Niben101Scf03860 | 488390-490386 | 1 |
| Niben101Scf03860 | 488613-495819 | 1 |
| Niben101Scf03860 | 488620-495826 | 1 |
| Niben101Scf03860 | 488651-490351 | 1 |
| Niben101Scf03860 | 488701-490627 | 1 |
| Niben101Scf03860 | 488721-490075 | 1 |
| Niben101Scf03860 | 488733-490045 | 1 |
| Niben101Scf03860 | 488737-490049 | 1 |
| Niben101Scf03860 | 488737-490403 | 1 |
| Niben101Scf03860 | 488741-490369 | 1 |
| Niben101Scf03860 | 488745-490373 | 1 |
| Niben101Scf03860 | 488784-490647 | 1 |
| Niben101Scf03860 | 488798-489977 | 1 |
| Niben101Scf03860 | 488853-490012 | 1 |
| Niben101Scf03860 | 488858-490017 | 1 |
| Niben101Scf03860 | 488886-490090 | 1 |
| Niben101Scf03860 | 488888-490081 | 1 |
| Niben101Scf03860 | 488895-490099 | 1 |
| Niben101Scf03860 | 488936-490129 | 1 |
| Niben101Scf03860 | 488958-490027 | 1 |
| Niben101Scf03860 | 488959-490028 | 1 |
| Niben101Scf03860 | 488970-490006 | 1 |
| Niben101Scf03860 | 489075-489946 | 1 |
| Niben101Scf03860 | 489104-489925 | 1 |
| Niben101Scf03860 | 489191-496555 | 1 |
| Niben101Scf03860 | 489197-496561 | 1 |
| Niben101Scf03860 | 489215-490034 | 1 |
| Niben101Scf03860 | 489223-489979 | 1 |
| Niben101Scf03860 | 489243-491550 | 1 |
| Niben101Scf03860 | 489247-491554 | 1 |
| Niben101Scf03860 | 489260-496493 | 1 |
| Niben101Scf03860 | 489263-489925 | 1 |
| Niben101Scf03860 | 489270-489932 | 1 |
| Niben101Scf03860 | 489284-490002 | 1 |
| Niben101Scf03860 | 489288-490006 | 1 |
| Niben101Scf03860 | 489299-490021 | 1 |
| Niben101Scf03860 | 489313-489918 | 1 |
| Niben101Scf03860 | 489317-489922 | 1 |
| Niben101Scf03860 | 489318-490024 | 1 |
| Niben101Scf03860 | 489328-489946 | 1 |
| Niben101Scf03860 | 489330-489978 | 1 |
| Niben101Scf03860 | 489336-490039 | 1 |
| Niben101Scf03860 | 489380-490096 | 1 |
| Niben101Scf03860 | 489392-490063 | 1 |
| Niben101Scf03860 | 489460-490033 | 1 |
| Niben101Scf03860 | 489485-489965 | 1 |
| Niben101Scf03860 | 489562-490067 | 1 |
| Niben101Scf03860 | 489566-490071 | 1 |
| Niben101Scf03860 | 489607-490104 | 1 |
| Niben101Scf03860 | 489619-489957 | 1 |
| Niben101Scf03860 | 489628-489966 | 1 |
| Niben101Scf03860 | 489637-490101 | 1 |
| Niben101Scf03860 | 489643-490111 | 1 |
| Niben101Scf03860 | 489669-490034 | 1 |
| Niben101Scf03860 | 489682-490048 | 1 |
| Niben101Scf03860 | 489690-490056 | 1 |
| Niben101Scf03860 | 489706-490056 | 1 |
| Niben101Scf03860 | 489717-490067 | 1 |
| Niben101Scf03860 | 489728-490375 | 1 |
| Niben101Scf03860 | 489733-490381 | 1 |
| Niben101Scf03860 | 489762-490076 | 1 |
| Niben101Scf03860 | 490187-497478 | 1 |
| Niben101Scf03860 | 490200-497316 | 1 |
| Niben101Scf03860 | 490336-497539 | 1 |
| Niben101Scf03860 | 490410-497792 | 1 |
| Niben101Scf03860 | 490425-497608 | 1 |
| Niben101Scf03860 | 490425-497793 | 1 |
| Niben101Scf03860 | 490430-497798 | 1 |
| Niben101Scf03860 | 490577-498286 | 1 |
| Niben101Scf03860 | 490631-492895 | 1 |
| Niben101Scf03860 | 490774-492755 | 1 |
| Niben101Scf03860 | 490795-491081 | 1 |
| Niben101Scf03860 | 490801-492343 | 1 |
| Niben101Scf03860 | 490803-491089 | 1 |
| Niben101Scf03860 | 490803-493052 | 1 |
| Niben101Scf03860 | 490805-492347 | 1 |
| Niben101Scf03860 | 490811-493060 | 1 |
| Niben101Scf03860 | 490827-491686 | 1 |
| Niben101Scf03860 | 490829-491571 | 1 |
| Niben101Scf03860 | 490834-498558 | 1 |
| Niben101Scf03860 | 490838-492066 | 1 |
| Niben101Scf03860 | 490847-491884 | 1 |
| Niben101Scf03860 | 490848-492155 | 1 |
| Niben101Scf03860 | 490851-491114 | 1 |
| Niben101Scf03860 | 490851-498578 | 1 |
| Niben101Scf03860 | 490857-491894 | 1 |
| Niben101Scf03860 | 490858-491895 | 1 |
| Niben101Scf03860 | 490860-491042 | 1 |
| Niben101Scf03860 | 490862-491044 | 1 |
| Niben101Scf03860 | 490867-498654 | 1 |
| Niben101Scf03860 | 490876-499177 | 1 |
| Niben101Scf03860 | 490892-499423 | 1 |
| Niben101Scf03860 | 490931-499086 | 1 |
| Niben101Scf03860 | 490936-499091 | 1 |
| Niben101Scf03860 | 490944-493271 | 1 |
| Niben101Scf03860 | 490945-499066 | 1 |
| Niben101Scf03860 | 490949-493278 | 1 |
| Niben101Scf03860 | 490950-493277 | 1 |
| Niben101Scf03860 | 490976-493228 | 1 |
| Niben101Scf03860 | 490994-493253 | 1 |
| Niben101Scf03860 | 490994-499096 | 1 |
| Niben101Scf03860 | 491031-493265 | 1 |
| Niben101Scf03860 | 491054-493209 | 1 |
| Niben101Scf03860 | 491067-499412 | 1 |
| Niben101Scf03860 | 491102-498983 | 1 |
| Niben101Scf03860 | 491105-498986 | 1 |
| Niben101Scf03860 | 491110-493273 | 1 |
| Niben101Scf03860 | 491117-493403 | 1 |
| Niben101Scf03860 | 491122-493408 | 1 |
| Niben101Scf03860 | 491129-492829 | 1 |
| Niben101Scf03860 | 491130-493341 | 1 |
| Niben101Scf03860 | 491131-492831 | 1 |
| Niben101Scf03860 | 491138-493349 | 1 |
| Niben101Scf03860 | 491141-498767 | 1 |
| Niben101Scf03860 | 491143-493144 | 1 |
| Niben101Scf03860 | 491170-499724 | 1 |
| Niben101Scf03860 | 491172-492904 | 1 |
| Niben101Scf03860 | 491175-499729 | 1 |
| Niben101Scf03860 | 491176-492908 | 1 |
| Niben101Scf03860 | 491181-492861 | 1 |
| Niben101Scf03860 | 491185-492762 | 1 |
| Niben101Scf03860 | 491186-492866 | 1 |
| Niben101Scf03860 | 491209-492935 | 1 |
| Niben101Scf03860 | 491228-498734 | 1 |
| Niben101Scf03860 | 491267-499409 | 1 |
| Niben101Scf03860 | 491282-498869 | 1 |
| Niben101Scf03860 | 491320-499406 | 1 |
| Niben101Scf03860 | 491326-499411 | 1 |
| Niben101Scf03860 | 491362-492729 | 1 |
| Niben101Scf03860 | 491367-493524 | 1 |
| Niben101Scf03860 | 491368-492735 | 1 |
| Niben101Scf03860 | 491398-492923 | 1 |
| Niben101Scf03860 | 491401-492926 | 1 |
| Niben101Scf03860 | 491462-492762 | 1 |
| Niben101Scf03860 | 491467-492767 | 1 |
| Niben101Scf03860 | 491471-493174 | 1 |
| Niben101Scf03860 | 491480-493183 | 1 |
| Niben101Scf03860 | 491486-493189 | 1 |
| Niben101Scf03860 | 491487-493649 | 1 |
| Niben101Scf03860 | 491487-493765 | 1 |
| Niben101Scf03860 | 491490-493193 | 1 |
| Niben101Scf03860 | 491491-493653 | 1 |
| Niben101Scf03860 | 491497-493734 | 1 |
| Niben101Scf03860 | 491499-493736 | 1 |
| Niben101Scf03860 | 491508-493780 | 1 |
| Niben101Scf03860 | 491513-493785 | 1 |
| Niben101Scf03860 | 491525-493779 | 1 |
| Niben101Scf03860 | 491528-493782 | 1 |
| Niben101Scf03860 | 491553-493485 | 1 |
| Niben101Scf03860 | 491565-492861 | 1 |
| Niben101Scf03860 | 491610-493057 | 1 |
| Niben101Scf03860 | 491617-492715 | 1 |
| Niben101Scf03860 | 491619-492717 | 1 |
| Niben101Scf03860 | 491619-493855 | 1 |
| Niben101Scf03860 | 491623-493859 | 1 |
| Niben101Scf03860 | 491637-499333 | 1 |
| Niben101Scf03860 | 491640-499336 | 1 |
| Niben101Scf03860 | 491677-492888 | 1 |
| Niben101Scf03860 | 491682-493365 | 1 |
| Niben101Scf03860 | 491684-493367 | 1 |
| Niben101Scf03860 | 491719-493710 | 1 |
| Niben101Scf03860 | 491723-493350 | 1 |
| Niben101Scf03860 | 491757-494105 | 1 |
| Niben101Scf03860 | 491774-493615 | 1 |
| Niben101Scf03860 | 491776-493617 | 1 |
| Niben101Scf03860 | 491786-499189 | 1 |
| Niben101Scf03860 | 491798-493264 | 1 |
| Niben101Scf03860 | 491806-493272 | 1 |
| Niben101Scf03860 | 491818-499740 | 1 |
| Niben101Scf03860 | 491828-499750 | 1 |
| Niben101Scf03860 | 491830-492665 | 1 |
| Niben101Scf03860 | 491831-499753 | 1 |
| Niben101Scf03860 | 491837-493835 | 1 |
| Niben101Scf03860 | 491850-492716 | 1 |
| Niben101Scf03860 | 491852-493850 | 1 |
| Niben101Scf03860 | 491857-493855 | 1 |
| Niben101Scf03860 | 491858-492672 | 1 |
| Niben101Scf03860 | 491858-492712 | 1 |
| Niben101Scf03860 | 491861-492675 | 1 |
| Niben101Scf03860 | 491878-492860 | 1 |
| Niben101Scf03860 | 491881-492863 | 1 |
| Niben101Scf03860 | 491882-494049 | 1 |
| Niben101Scf03860 | 491897-493901 | 1 |
| Niben101Scf03860 | 491898-493053 | 1 |
| Niben101Scf03860 | 491900-493055 | 1 |
| Niben101Scf03860 | 491907-493911 | 1 |
| Niben101Scf03860 | 491909-493076 | 1 |
| Niben101Scf03860 | 491910-492720 | 1 |
| Niben101Scf03860 | 491912-493079 | 1 |
| Niben101Scf03860 | 491935-492609 | 1 |
| Niben101Scf03860 | 491941-492615 | 1 |
| Niben101Scf03860 | 491944-493451 | 1 |
| Niben101Scf03860 | 491949-492711 | 1 |
| Niben101Scf03860 | 491962-493882 | 1 |
| Niben101Scf03860 | 491965-493498 | 1 |
| Niben101Scf03860 | 491967-493887 | 1 |
| Niben101Scf03860 | 491970-493208 | 1 |
| Niben101Scf03860 | 491975-494216 | 1 |
| Niben101Scf03860 | 491976-493214 | 1 |
| Niben101Scf03860 | 491976-493518 | 1 |
| Niben101Scf03860 | 491982-493524 | 1 |
| Niben101Scf03860 | 492005-493073 | 1 |
| Niben101Scf03860 | 492010-493858 | 1 |
| Niben101Scf03860 | 492025-493124 | 1 |
| Niben101Scf03860 | 492060-492728 | 1 |
| Niben101Scf03860 | 492067-492735 | 1 |
| Niben101Scf03860 | 492072-493389 | 1 |
| Niben101Scf03860 | 492077-493394 | 1 |
| Niben101Scf03860 | 492080-499748 | 1 |
| Niben101Scf03860 | 492084-492845 | 1 |
| Niben101Scf03860 | 492086-492722 | 1 |
| Niben101Scf03860 | 492087-492986 | 1 |
| Niben101Scf03860 | 492092-499760 | 1 |
| Niben101Scf03860 | 492098-499766 | 1 |
| Niben101Scf03860 | 492101-499712 | 1 |
| Niben101Scf03860 | 492102-494062 | 1 |
| Niben101Scf03860 | 492106-494066 | 1 |
| Niben101Scf03860 | 492116-493529 | 1 |
| Niben101Scf03860 | 492148-493212 | 1 |
| Niben101Scf03860 | 492153-492730 | 1 |
| Niben101Scf03860 | 492176-493809 | 1 |
| Niben101Scf03860 | 492177-494206 | 1 |
| Niben101Scf03860 | 492180-494209 | 1 |
| Niben101Scf03860 | 492185-493905 | 1 |
| Niben101Scf03860 | 492190-493910 | 1 |
| Niben101Scf03860 | 492199-494503 | 1 |
| Niben101Scf03860 | 492203-493384 | 1 |
| Niben101Scf03860 | 492204-493385 | 1 |
| Niben101Scf03860 | 492208-494009 | 1 |
| Niben101Scf03860 | 492212-494013 | 1 |
| Niben101Scf03860 | 492226-494029 | 1 |
| Niben101Scf03860 | 492226-494126 | 1 |
| Niben101Scf03860 | 492228-494031 | 1 |
| Niben101Scf03860 | 492230-494130 | 1 |
| Niben101Scf03860 | 492232-493471 | 1 |
| Niben101Scf03860 | 492236-494015 | 1 |
| Niben101Scf03860 | 492249-493789 | 1 |
| Niben101Scf03860 | 492254-493794 | 1 |
| Niben101Scf03860 | 492261-493952 | 1 |
| Niben101Scf03860 | 492265-493956 | 1 |
| Niben101Scf03860 | 492265-494543 | 1 |
| Niben101Scf03860 | 492267-493820 | 1 |
| Niben101Scf03860 | 492271-493208 | 1 |
| Niben101Scf03860 | 492273-493826 | 1 |
| Niben101Scf03860 | 492275-493395 | 1 |
| Niben101Scf03860 | 492278-493398 | 1 |
| Niben101Scf03860 | 492282-494102 | 1 |
| Niben101Scf03860 | 492293-492771 | 1 |
| Niben101Scf03860 | 492303-492781 | 1 |
| Niben101Scf03860 | 492303-493036 | 1 |
| Niben101Scf03860 | 492307-493040 | 1 |
| Niben101Scf03860 | 492348-494102 | 1 |
| Niben101Scf03860 | 492352-494106 | 1 |
| Niben101Scf03860 | 492355-493078 | 1 |
| Niben101Scf03860 | 492356-493575 | 1 |
| Niben101Scf03860 | 492361-493580 | 1 |
| Niben101Scf03860 | 492374-493272 | 1 |
| Niben101Scf03860 | 492374-499778 | 1 |
| Niben101Scf03860 | 492378-493511 | 1 |
| Niben101Scf03860 | 492379-494531 | 1 |
| Niben101Scf03860 | 492380-492753 | 1 |
| Niben101Scf03860 | 492382-493384 | 1 |
| Niben101Scf03860 | 492400-493796 | 1 |
| Niben101Scf03860 | 492410-492969 | 1 |
| Niben101Scf03860 | 492434-494086 | 1 |
| Niben101Scf03860 | 492450-494544 | 1 |
| Niben101Scf03860 | 492452-493251 | 1 |
| Niben101Scf03860 | 492455-494299 | 1 |
| Niben101Scf03860 | 492457-494661 | 1 |
| Niben101Scf03860 | 492458-494114 | 1 |
| Niben101Scf03860 | 492458-494302 | 1 |
| Niben101Scf03860 | 492458-494552 | 1 |
| Niben101Scf03860 | 492461-499865 | 1 |
| Niben101Scf03860 | 492462-494666 | 1 |
| Niben101Scf03860 | 492468-494123 | 1 |
| Niben101Scf03860 | 492469-494151 | 1 |
| Niben101Scf03860 | 492472-494154 | 1 |
| Niben101Scf03860 | 492484-493042 | 1 |
| Niben101Scf03860 | 492496-493449 | 1 |
| Niben101Scf03860 | 492497-492866 | 1 |
| Niben101Scf03860 | 492511-494295 | 1 |
| Niben101Scf03860 | 492515-494299 | 1 |
| Niben101Scf03860 | 492516-494330 | 1 |
| Niben101Scf03860 | 492521-494335 | 1 |
| Niben101Scf03860 | 492524-492937 | 1 |
| Niben101Scf03860 | 492524-494331 | 1 |
| Niben101Scf03860 | 492527-493069 | 1 |
| Niben101Scf03860 | 492528-493070 | 1 |
| Niben101Scf03860 | 492530-492943 | 1 |
| Niben101Scf03860 | 492538-493080 | 1 |
| Niben101Scf03860 | 492553-493226 | 1 |
| Niben101Scf03860 | 492555-493408 | 1 |
| Niben101Scf03860 | 492562-493415 | 1 |
| Niben101Scf03860 | 495826-498091 | 1 |
| Niben101Scf03860 | 495834-498099 | 1 |
| Niben101Scf03860 | 495853-498117 | 1 |
| Niben101Scf03860 | 495856-498120 | 1 |
| Niben101Scf03860 | 495877-497855 | 1 |
| Niben101Scf03860 | 495881-497818 | 1 |
| Niben101Scf03860 | 495885-497822 | 1 |
| Niben101Scf03860 | 495887-497969 | 1 |
| Niben101Scf03860 | 495896-498052 | 1 |
| Niben101Scf03860 | 495904-498060 | 1 |
| Niben101Scf03860 | 495913-498088 | 1 |
| Niben101Scf03860 | 496032-497977 | 1 |
| Niben101Scf03860 | 496036-497981 | 1 |
| Niben101Scf03860 | 496048-498331 | 1 |
| Niben101Scf03860 | 496096-497827 | 1 |
| Niben101Scf03860 | 496098-497829 | 1 |
| Niben101Scf03860 | 496115-497981 | 1 |
| Niben101Scf03860 | 496141-498286 | 1 |
| Niben101Scf03860 | 496164-498069 | 1 |
| Niben101Scf03860 | 496170-498075 | 1 |
| Niben101Scf03860 | 496176-498232 | 1 |
| Niben101Scf03860 | 496182-498238 | 1 |
| Niben101Scf03860 | 496198-498205 | 1 |
| Niben101Scf03860 | 496200-498090 | 1 |
| Niben101Scf03860 | 496200-498517 | 1 |
| Niben101Scf03860 | 496201-498518 | 1 |
| Niben101Scf03860 | 496229-498629 | 1 |
| Niben101Scf03860 | 496288-497837 | 1 |
| Niben101Scf03860 | 496289-497838 | 1 |
| Niben101Scf03860 | 496290-497839 | 1 |
| Niben101Scf03860 | 496299-498696 | 1 |
| Niben101Scf03860 | 496352-498188 | 1 |
| Niben101Scf03860 | 496363-498684 | 1 |
| Niben101Scf03860 | 496370-498691 | 1 |
| Niben101Scf03860 | 496376-498662 | 1 |
| Niben101Scf03860 | 496384-498670 | 1 |
| Niben101Scf03860 | 496393-498679 | 1 |
| Niben101Scf03860 | 496400-498672 | 1 |
| Niben101Scf03860 | 496405-498677 | 1 |
| Niben101Scf03860 | 496411-498697 | 1 |
| Niben101Scf03860 | 496435-498245 | 1 |
| Niben101Scf03860 | 496439-498249 | 1 |
| Niben101Scf03860 | 496445-498408 | 1 |
| Niben101Scf03860 | 496466-498711 | 1 |
| Niben101Scf03860 | 496469-498714 | 1 |
| Niben101Scf03860 | 496480-498725 | 1 |
| Niben101Scf03860 | 496489-498734 | 1 |
| Niben101Scf03860 | 496504-498748 | 1 |
| Niben101Scf03860 | 496515-498759 | 1 |
| Niben101Scf03860 | 496519-498763 | 1 |
| Niben101Scf03860 | 496524-498768 | 1 |
| Niben101Scf03860 | 496525-498769 | 1 |
| Niben101Scf03860 | 496530-498775 | 1 |
| Niben101Scf03860 | 496533-498777 | 1 |
| Niben101Scf03860 | 496535-498532 | 1 |
| Niben101Scf03860 | 496535-498779 | 1 |
| Niben101Scf03860 | 496546-498790 | 1 |
| Niben101Scf03860 | 496548-498793 | 1 |
| Niben101Scf03860 | 496552-498796 | 1 |
| Niben101Scf03860 | 496559-498804 | 1 |
| Niben101Scf03860 | 496563-498808 | 1 |
| Niben101Scf03860 | 496571-498181 | 1 |
| Niben101Scf03860 | 496575-498820 | 1 |
| Niben101Scf03860 | 496576-498186 | 1 |
| Niben101Scf03860 | 496576-498821 | 1 |
| Niben101Scf03860 | 496586-498484 | 1 |
| Niben101Scf03860 | 496587-498832 | 1 |
| Niben101Scf03860 | 496589-498529 | 1 |
| Niben101Scf03860 | 496590-498488 | 1 |
| Niben101Scf03860 | 496593-498533 | 1 |
| Niben101Scf03860 | 496616-498656 | 1 |
| Niben101Scf03860 | 496616-498862 | 1 |
| Niben101Scf03860 | 496631-497927 | 1 |
| Niben101Scf03860 | 496635-497931 | 1 |
| Niben101Scf03860 | 496636-498577 | 1 |
| Niben101Scf03860 | 496647-498893 | 1 |
| Niben101Scf03860 | 496658-498904 | 1 |
| Niben101Scf03860 | 496669-498915 | 1 |
| Niben101Scf03860 | 496684-498930 | 1 |
| Niben101Scf03860 | 496688-498934 | 1 |
| Niben101Scf03860 | 496693-498939 | 1 |
| Niben101Scf03860 | 496694-498940 | 1 |
| Niben101Scf03860 | 496720-498532 | 1 |
| Niben101Scf03860 | 496722-498500 | 1 |
| Niben101Scf03860 | 496726-498538 | 1 |
| Niben101Scf03860 | 496735-498981 | 1 |
| Niben101Scf03860 | 496749-498995 | 1 |
| Niben101Scf03860 | 496753-498557 | 1 |
| Niben101Scf03860 | 496760-499006 | 1 |
| Niben101Scf03860 | 496761-498565 | 1 |
| Niben101Scf03860 | 496764-499010 | 1 |
| Niben101Scf03860 | 496775-499021 | 1 |
| Niben101Scf03860 | 496777-498878 | 1 |
| Niben101Scf03860 | 496783-499029 | 1 |
| Niben101Scf03860 | 496795-499041 | 1 |
| Niben101Scf03860 | 496805-498502 | 1 |
| Niben101Scf03860 | 496808-498517 | 1 |
| Niben101Scf03860 | 496816-498657 | 1 |
| Niben101Scf03860 | 496821-497976 | 1 |
| Niben101Scf03860 | 496842-498839 | 1 |
| Niben101Scf03860 | 496849-498846 | 1 |
| Niben101Scf03860 | 496849-499096 | 1 |
| Niben101Scf03860 | 496858-497875 | 1 |
| Niben101Scf03860 | 496863-497880 | 1 |
| Niben101Scf03860 | 496868-499115 | 1 |
| Niben101Scf03860 | 496877-497903 | 1 |
| Niben101Scf03860 | 496883-497909 | 1 |
| Niben101Scf03860 | 496885-499135 | 1 |
| Niben101Scf03860 | 496900-499150 | 1 |
| Niben101Scf03860 | 496918-499168 | 1 |
| Niben101Scf03860 | 496940-499184 | 1 |
| Niben101Scf03860 | 496945-499189 | 1 |
| Niben101Scf03860 | 496971-499215 | 1 |
| Niben101Scf03860 | 496980-497835 | 1 |
| Niben101Scf03860 | 496980-499182 | 1 |
| Niben101Scf03860 | 496982-497880 | 1 |
| Niben101Scf03860 | 496986-499188 | 1 |
| Niben101Scf03860 | 497005-499249 | 1 |
| Niben101Scf03860 | 497017-499261 | 1 |
| Niben101Scf03860 | 497024-499268 | 1 |
| Niben101Scf03860 | 497053-499297 | 1 |
| Niben101Scf03860 | 497061-499139 | 1 |
| Niben101Scf03860 | 497073-499317 | 1 |
| Niben101Scf03860 | 497089-497932 | 1 |
| Niben101Scf03860 | 497139-497865 | 1 |
| Niben101Scf03860 | 497152-499398 | 1 |
| Niben101Scf03860 | 497166-499411 | 1 |
| Niben101Scf03860 | 497194-499439 | 1 |
| Niben101Scf03860 | 497210-499455 | 1 |
| Niben101Scf03860 | 497233-499478 | 1 |
| Niben101Scf03860 | 497330-499575 | 1 |
| Niben101Scf03860 | 497366-498497 | 1 |
| Niben101Scf03860 | 497369-498602 | 1 |
| Niben101Scf03860 | 497371-499616 | 1 |
| Niben101Scf03860 | 497372-498605 | 1 |
| Niben101Scf03860 | 497373-498324 | 1 |
| Niben101Scf03860 | 497378-499623 | 1 |
| Niben101Scf03860 | 497379-499624 | 1 |
| Niben101Scf03860 | 497384-499629 | 1 |
| Niben101Scf03860 | 497393-499675 | 1 |
| Niben101Scf03860 | 497397-499679 | 1 |
| Niben101Scf03860 | 497405-499671 | 1 |
| Niben101Scf03860 | 497408-499674 | 1 |
| Niben101Scf03860 | 497425-497939 | 1 |
| Niben101Scf03860 | 497425-498637 | 1 |
| Niben101Scf03860 | 497438-497958 | 1 |
| Niben101Scf03860 | 497450-498751 | 1 |
| Niben101Scf03860 | 497457-498758 | 1 |
| Niben101Scf03860 | 497460-498662 | 1 |
| Niben101Scf03860 | 497461-499858 | 1 |
| Niben101Scf03860 | 497467-498669 | 1 |
| Niben101Scf03860 | 497471-497999 | 1 |
| Niben101Scf03860 | 497475-498249 | 1 |
| Niben101Scf03860 | 497477-498251 | 1 |
| Niben101Scf03860 | 497479-498007 | 1 |
| Niben101Scf03860 | 497479-499880 | 1 |
| Niben101Scf03860 | 497540-499411 | 1 |
| Niben101Scf03860 | 497556-499041 | 1 |
| Niben101Scf03860 | 497656-499377 | 1 |
| Niben101Scf03860 | 497660-499381 | 1 |
| Niben101Scf03860 | 497669-499140 | 1 |
| Niben101Scf03860 | 497673-499549 | 1 |
| Niben101Scf03860 | 497678-498490 | 1 |
| Niben101Scf03860 | 497682-498777 | 1 |
| Niben101Scf03860 | 497683-498531 | 1 |
| Niben101Scf03860 | 497683-499559 | 1 |
| Niben101Scf03860 | 497717-498025 | 1 |
| Niben101Scf03860 | 497738-499693 | 1 |
| Niben101Scf03860 | 497765-499861 | 1 |
| Niben101Scf03860 | 497783-498658 | 1 |
| Niben101Scf03918 | 78280-78701 | 1 |
| Niben101Scf03918 | 78283-78704 | 1 |
| Niben101Scf04083 | 422071-422921 | 1 |
| Niben101Scf04083 | 422074-422924 | 1 |
| Niben101Scf04184 | 729400-730296 | 1 |
| Niben101Scf04184 | 729511-730216 | 1 |
| Niben101Scf04198 | 455387-455801 | 1 |
| Niben101Scf04329 | 362815-363332 | 1 |
| Niben101Scf04329 | 362819-363336 | 1 |
| Niben101Scf04398 | 269956-270829 | 1 |
| Niben101Scf04398 | 269957-270830 | 1 |
| Niben101Scf04398 | 269964-270837 | 1 |
| Niben101Scf04537 | 18510-18644 | 1 |
| Niben101Scf04627 | 518734-519383 | 1 |
| Niben101Scf04639 | 646914-647668 | 1 |
| Niben101Scf04664 | 165318-167450 | 1 |
| Niben101Scf04706 | 207896-208707 | 1 |
| Niben101Scf04706 | 207901-208712 | 1 |
| Niben101Scf04745 | 545541-548456 | 1 |
| Niben101Scf04745 | 545547-548462 | 1 |
| Niben101Scf04745 | 546676-547703 | 1 |
| Niben101Scf04745 | 546858-548449 | 1 |
| Niben101Scf04872 | 119068-120186 | 1 |
| Niben101Scf04901 | 114318-115101 | 1 |
| Niben101Scf04901 | 114328-115111 | 1 |
| Niben101Scf05017 | 485709-486447 | 1 |
| Niben101Scf05035 | 567147-569323 | 1 |
| Niben101Scf05124 | 127331-127923 | 1 |
| Niben101Scf05135 | 461080-461460 | 1 |
| Niben101Scf05135 | 461087-461467 | 1 |
| Niben101Scf05229 | 198723-199025 | 1 |
| Niben101Scf05283 | 42114-42853 | 1 |
| Niben101Scf05283 | 42119-42858 | 1 |
| Niben101Scf05283 | 42133-42316 | 1 |
| Niben101Scf05283 | 42137-42320 | 1 |
| Niben101Scf05283 | 42235-42627 | 1 |
| Niben101Scf05283 | 42237-43137 | 1 |
| Niben101Scf05283 | 42240-42632 | 1 |
| Niben101Scf05283 | 42280-42546 | 1 |
| Niben101Scf05283 | 42288-42554 | 1 |
| Niben101Scf05317 | 69886-71276 | 1 |
| Niben101Scf05422 | 127735-128457 | 1 |
| Niben101Scf05447 | 190453-190836 | 1 |
| Niben101Scf05447 | 190458-191097 | 1 |
| Niben101Scf05447 | 190459-191098 | 1 |
| Niben101Scf05609 | 286448-286884 | 1 |
| Niben101Scf05609 | 287109-287859 | 1 |
| Niben101Scf05732 | 365956-366941 | 1 |
| Niben101Scf05732 | 365960-366945 | 1 |
| Niben101Scf05767 | 76429-77221 | 1 |
| Niben101Scf05767 | 76435-77227 | 1 |
| Niben101Scf05830 | 97209-98162 | 1 |
| Niben101Scf05880 | 341112-342085 | 1 |
| Niben101Scf05880 | 341117-342090 | 1 |
| Niben101Scf05952 | 5154-6289 | 1 |
| Niben101Scf06222 | 125582-126368 | 1 |
| Niben101Scf06222 | 125587-126373 | 1 |
| Niben101Scf06240 | 49226-49940 | 1 |
| Niben101Scf06240 | 49229-49943 | 1 |
| Niben101Scf06240 | 52141-53541 | 1 |
| Niben101Scf06240 | 52148-53548 | 1 |
| Niben101Scf06240 | 52258-53301 | 1 |
| Niben101Scf06240 | 52675-53027 | 1 |
| Niben101Scf06240 | 57360-58497 | 1 |
| Niben101Scf06240 | 57363-58500 | 1 |
| Niben101Scf06286 | 73310-74423 | 1 |
| Niben101Scf06290 | 199857-200476 | 1 |
| Niben101Scf06437 | 466609-467005 | 1 |
| Niben101Scf06674 | 104064-104645 | 1 |
| Niben101Scf06674 | 104068-104649 | 1 |
| Niben101Scf06806 | 330725-332003 | 1 |
| Niben101Scf06949 | 202027-202832 | 1 |
| Niben101Scf06998 | 127523-128226 | 1 |
| Niben101Scf07026 | 26649-26905 | 1 |
| Niben101Scf07026 | 26655-26911 | 1 |
| Niben101Scf07049 | 179304-179592 | 1 |
| Niben101Scf07382 | 179925-181928 | 1 |
| Niben101Scf07382 | 179929-181932 | 1 |
| Niben101Scf07386 | 56403-58464 | 1 |
| Niben101Scf07386 | 56406-58467 | 1 |
| Niben101Scf07441 | 132100-133347 | 1 |
| Niben101Scf07441 | 132383-132624 | 1 |
| Niben101Scf07441 | 132386-132681 | 1 |
| Niben101Scf07441 | 132393-132688 | 1 |
| Niben101Scf07554 | 308018-309076 | 1 |
| Niben101Scf07554 | 308021-309079 | 1 |
| Niben101Scf07554 | 308022-309080 | 1 |
| Niben101Scf07664 | 202986-203773 | 1 |
| Niben101Scf07850 | 804939-805531 | 1 |
| Niben101Scf07939 | 114907-115369 | 1 |
| Niben101Scf08034 | 113986-115436 | 1 |
| Niben101Scf08034 | 113990-115440 | 1 |
| Niben101Scf08044 | 249815-250624 | 1 |
| Niben101Scf08044 | 249823-250632 | 1 |
| Niben101Scf08108 | 45175-46027 | 1 |
| Niben101Scf08111 | 227013-227780 | 1 |
| Niben101Scf08134 | 156740-158695 | 1 |
| Niben101Scf08134 | 156744-158699 | 1 |
| Niben101Scf08222 | 415906-416684 | 1 |
| Niben101Scf08222 | 415915-416693 | 1 |
| Niben101Scf08680 | 343351-346144 | 1 |
| Niben101Scf08738 | 37175-37971 | 1 |
| Niben101Scf08899 | 86887-87536 | 1 |
| Niben101Scf08947 | 135876-136456 | 1 |
| Niben101Scf08947 | 135879-136459 | 1 |
| Niben101Scf09170 | 314093-315012 | 1 |
| Niben101Scf09230 | 459982-460610 | 1 |
| Niben101Scf09230 | 459993-460621 | 1 |
| Niben101Scf09590 | 358948-359576 | 1 |
| Niben101Scf09590 | 358994-359544 | 1 |
| Niben101Scf09590 | 358998-359548 | 1 |
| Niben101Scf10092 | 92655-93041 | 1 |
| Niben101Scf10535 | 1104-2249 | 1 |
| Niben101Scf10535 | 1110-2255 | 1 |
| Niben101Scf10627 | 278092-278546 | 1 |
| Niben101Scf10904 | 21840-22468 | 1 |
| Niben101Scf10904 | 21845-22473 | 1 |
| Niben101Scf11178 | 172896-173720 | 1 |
| Niben101Scf11178 | 178573-179303 | 1 |
| Niben101Scf11306 | 46725-47889 | 1 |
| Niben101Scf11361 | 284902-285409 | 1 |
| Niben101Scf11361 | 284906-285413 | 1 |
| Niben101Scf11383 | 11121-11618 | 1 |
| Niben101Scf11535 | 99376-100075 | 1 |
| Niben101Scf11653 | 78180-80971 | 1 |
| Niben101Scf11653 | 78186-80977 | 1 |
| Niben101Scf11653 | 78845-80955 | 1 |
| Niben101Scf11653 | 78851-80961 | 1 |
| Niben101Scf11756 | 388875-389388 | 1 |
| Niben101Scf11756 | 388881-389394 | 1 |
| Niben101Scf11756 | 389041-389156 | 1 |
| Niben101Scf11756 | 389046-389161 | 1 |
| Niben101Scf12084 | 91376-92980 | 1 |
| Niben101Scf12084 | 91414-93034 | 1 |
| Niben101Scf12084 | 91421-93127 | 1 |
| Niben101Scf12582 | 356637-357442 | 1 |
| Niben101Scf12694 | 25778-26419 | 1 |
| Niben101Scf12694 | 25783-26424 | 1 |
| Niben101Scf12735 | 84893-85661 | 1 |
| Niben101Scf13180 | 223868-224976 | 1 |
| Niben101Scf13180 | 223868-224977 | 1 |
| Niben101Scf13180 | 223868-224978 | 1 |
| Niben101Scf13180 | 223869-224978 | 1 |
| Niben101Scf13180 | 223872-224830 | 1 |
| Niben101Scf13180 | 223874-224982 | 1 |
| Niben101Scf13180 | 223878-224836 | 1 |
| Niben101Scf13180 | 224078-224938 | 1 |
| Niben101Scf13180 | 224083-224943 | 1 |
| Niben101Scf13180 | 224165-224953 | 1 |
| Niben101Scf13180 | 224168-224956 | 1 |
| Niben101Scf13180 | 224202-224951 | 1 |
| Niben101Scf13180 | 224207-224956 | 1 |
| Niben101Scf13777 | 15107-16237 | 1 |
| Niben101Scf14115 | 100779-102011 | 1 |
| Niben101Scf14115 | 100782-102014 | 1 |
| Niben101Scf14320 | 114861-115697 | 1 |
| Niben101Scf14320 | 115049-115610 | 1 |
| Niben101Scf14320 | 115052-115613 | 1 |
| Niben101Scf14320 | 133723-134825 | 1 |
| Niben101Scf14320 | 133726-134828 | 1 |
| Niben101Scf14859 | 39054-41168 | 1 |
| Niben101Scf15187 | 11004-12550 | 1 |
| Niben101Scf15187 | 11007-12553 | 1 |
| Niben101Scf15187 | 11088-12315 | 1 |
| Niben101Scf15689 | 48521-49332 | 1 |
| Niben101Scf15809 | 161992-163380 | 1 |
| Niben101Scf16939 | 57217-58480 | 1 |
| Niben101Scf18068 | 130770-131422 | 1 |
| Niben101Scf18902 | 15115-15622 | 1 |
| Niben101Scf23843 | 80587-81213 | 1 |
| Niben101Scf23843 | 80590-81216 | 1 |
| Niben101Scf23843 | 80746-81285 | 1 |
| Niben101Scf24679 | 71439-72626 | 1 |
| Niben101Scf27324 | 1438-11811 | 1 |
| Niben101Scf29235 | 7121-7562 | 1 |
| Niben101Scf29276 | 7236-7663 | 1 |
| Niben101Scf29276 | 7243-7670 | 1 |

**Table S4: List of *N. tabacum* miRNAs with their number of binding sites on 2099 *N. benthamiana* circRNAs**

| *N. tabacum* miRNA | No. of binding sites |
| --- | --- |
| nta-miR1446 | 13 |
| nta-miR156a | 77 |
| nta-miR156b | 77 |
| nta-miR156c | 77 |
| nta-miR156d | 77 |
| nta-miR156e | 77 |
| nta-miR156f | 63 |
| nta-miR156g | 12 |
| nta-miR156h | 12 |
| nta-miR156i | 12 |
| nta-miR156j | 12 |
| nta-miR159 | 53 |
| nta-miR160a | 3 |
| nta-miR160b | 3 |
| nta-miR160c | 3 |
| nta-miR160d | 5 |
| nta-miR162a | 6 |
| nta-miR162b | 6 |
| nta-miR164a | 32 |
| nta-miR164b | 32 |
| nta-miR164c | 73 |
| nta-miR166a | 7 |
| nta-miR166b | 7 |
| nta-miR166c | 7 |
| nta-miR166d | 7 |
| nta-miR166e | 7 |
| nta-miR166f | 7 |
| nta-miR166g | 7 |
| nta-miR166h | 7 |
| nta-miR167a | 7 |
| nta-miR167b | 7 |
| nta-miR167c | 7 |
| nta-miR167d | 9 |
| nta-miR167e | 9 |
| nta-miR168a | 11 |
| nta-miR168b | 11 |
| nta-miR168c | 11 |
| nta-miR168d | 13 |
| nta-miR168e | 13 |
| nta-miR169a | 16 |
| nta-miR169b | 16 |
| nta-miR169c | 16 |
| nta-miR169d | 16 |
| nta-miR169e | 16 |
| nta-miR169f | 16 |
| nta-miR169g | 16 |
| nta-miR169h | 16 |
| nta-miR169i | 16 |
| nta-miR169j | 16 |
| nta-miR169k | 16 |
| nta-miR169l | 16 |
| nta-miR169m | 16 |
| nta-miR169o | 16 |
| nta-miR169p | 16 |
| nta-miR169q | 15 |
| nta-miR169r | 15 |
| nta-miR169s | 15 |
| nta-miR169t | 17 |
| nta-miR171a | 34 |
| nta-miR171b | 27 |
| nta-miR171c | 7 |
| nta-miR172a | 63 |
| nta-miR172b | 27 |
| nta-miR172c | 72 |
| nta-miR172d | 72 |
| nta-miR172e | 72 |
| nta-miR172f | 72 |
| nta-miR172g | 72 |
| nta-miR172h | 72 |
| nta-miR172i | 72 |
| nta-miR172j | 25 |
| nta-miR1919 | 20 |
| nta-miR319a | 41 |
| nta-miR319b | 41 |
| nta-miR390a | 61 |
| nta-miR390b | 50 |
| nta-miR390c | 50 |
| nta-miR394 | 12 |
| nta-miR395a | 197 |
| nta-miR395b | 197 |
| nta-miR395c | 197 |
| nta-miR396a | 31 |
| nta-miR396b | 37 |
| nta-miR396c | 37 |
| nta-miR397 | 145 |
| nta-miR398 | 5 |
| nta-miR399a | 16 |
| nta-miR399b | 16 |
| nta-miR399c | 16 |
| nta-miR399d | 16 |
| nta-miR399e | 16 |
| nta-miR399f | 16 |
| nta-miR399g | 16 |
| nta-miR408 | 161 |
| nta-miR477a | 46 |
| nta-miR477b | 13 |
| nta-miR479a | 85 |
| nta-miR479b | 187 |
| nta-miR482a | 192 |
| nta-miR482b-5p | 74 |
| nta-miR482c | 192 |
| nta-miR482d | 3 |
| nta-miR5303a | 21 |
| nta-miR5303b | 21 |
| nta-miR5303c | 31 |
| nta-miR6019a | 18 |
| nta-miR6019b | 18 |
| nta-miR6020a-3p | 16 |
| nta-miR6020a-5p | 42 |
| nta-miR6020b | 192 |
| nta-miR6021 | 46 |
| nta-miR6024 | 26 |
| nta-miR6025a | 9 |
| nta-miR6025b | 18 |
| nta-miR6025c | 47 |
| nta-miR6025d | 33 |
| nta-miR6025e | 23 |
| nta-miR6144 | 185 |
| nta-miR6145a | 61 |
| nta-miR6145b | 11 |
| nta-miR6145c | 22 |
| nta-miR6145d | 49 |
| nta-miR6145e | 45 |
| nta-miR6145f | 35 |
| nta-miR6146a | 24 |
| nta-miR6146b | 33 |
| nta-miR6147 | 14 |
| nta-miR6148a | 11 |
| nta-miR6148b | 201 |
| nta-miR6149a | 14 |
| nta-miR6149b | 14 |
| nta-miR6150 | 197 |
| nta-miR6151a | 125 |
| nta-miR6151b | 125 |
| nta-miR6151c | 125 |
| nta-miR6151d | 125 |
| nta-miR6151e | 125 |
| nta-miR6151f | 140 |
| nta-miR6151g | 119 |
| nta-miR6151h | 119 |
| nta-miR6151i | 119 |
| nta-miR6152a | 12 |
| nta-miR6152b | 12 |
| nta-miR6153 | 41 |
| nta-miR6154a | 135 |
| nta-miR6154b | 135 |
| nta-miR6155 | 125 |
| nta-miR6156 | 68 |
| nta-miR6157 | 30 |
| nta-miR6158a | 49 |
| nta-miR6158b | 49 |
| nta-miR6158c | 49 |
| nta-miR6159 | 16 |
| nta-miR6160 | 11 |
| nta-miR6161a | 11 |
| nta-miR6161b | 11 |
| nta-miR6161c | 38 |
| nta-miR6161d | 37 |
| nta-miR6162 | 72 |
| nta-miR6163 | 10 |
| nta-miR6164a | 198 |
| nta-miR6164b | 198 |
| nta-miR827 | 189 |

**Supplemental Figures**

**a**
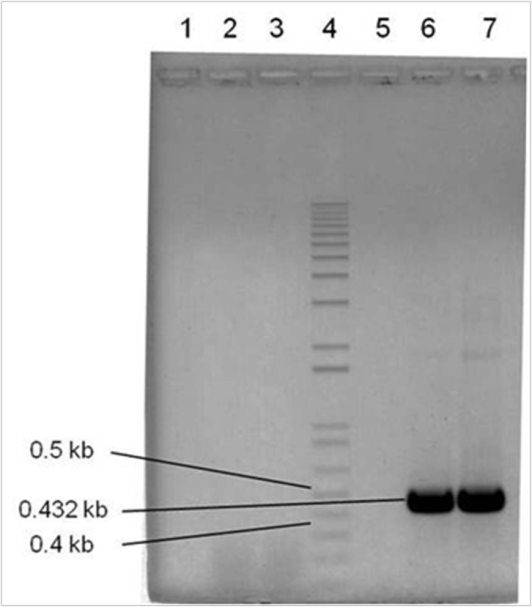
 **b**
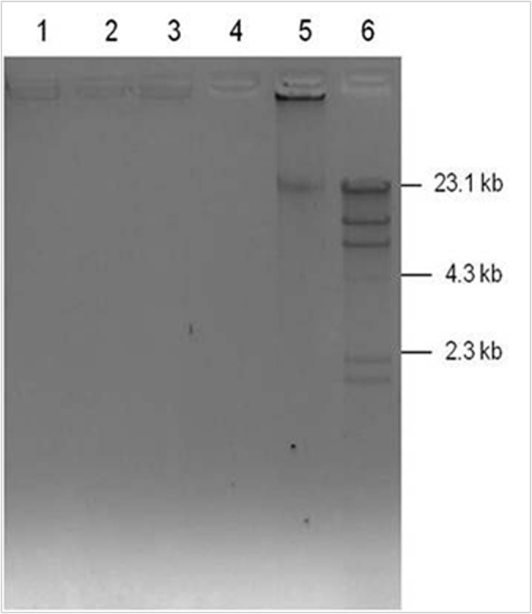


**Figure S1.** Absence of TIA in MDA**. a** PCR was carried out with BYVMV C2 primer shows the amplicon of 432bp in total RNA+APTR9 (lane6) and APTR9 (lane7) whereas water control (lane1), total RNA (lane2), DNase treated RNA (lane3) and DNase treated (RNA+APTR9) (lane5) do not show any amplification shows the efficient degradation of DNA from extracted RNA and plasmid DNA (APTR9). **b** MDA was performed without any template with 50μm (lane1), 100μm (lane2), 150μm (lane3) and 200μm (lane4) exo-resistant random pentamer with blocked 5’ ends do not show any product whereas 50μm of same primer with 50ng APTR9 template (lane5) shows amplicon confirms td-MDA. Generuler 1kb plus DNA ladder is loaded in lane4 of **a** and λHindIII marker in lane6 of **b**.

**a b**


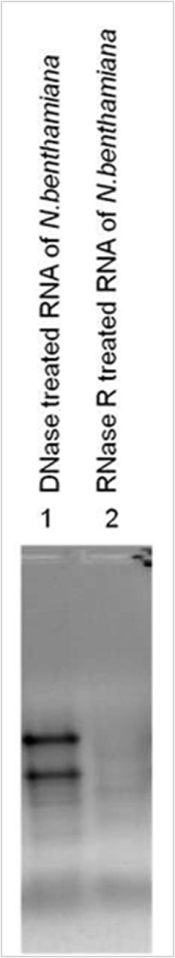

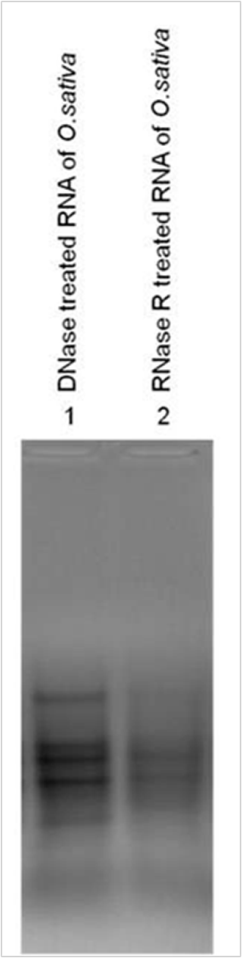


**Figure S2. RNA Extraction from *N. benthamiana* and *O. sativa* Plants.** Total RNA was extracted from *N. benthamiana* (**a**) and *O. sativa* (**b**) and treated with DNase (lane1) to remove DNA contamination and RNase R (lane2) to remove linear RNAs.

**a**


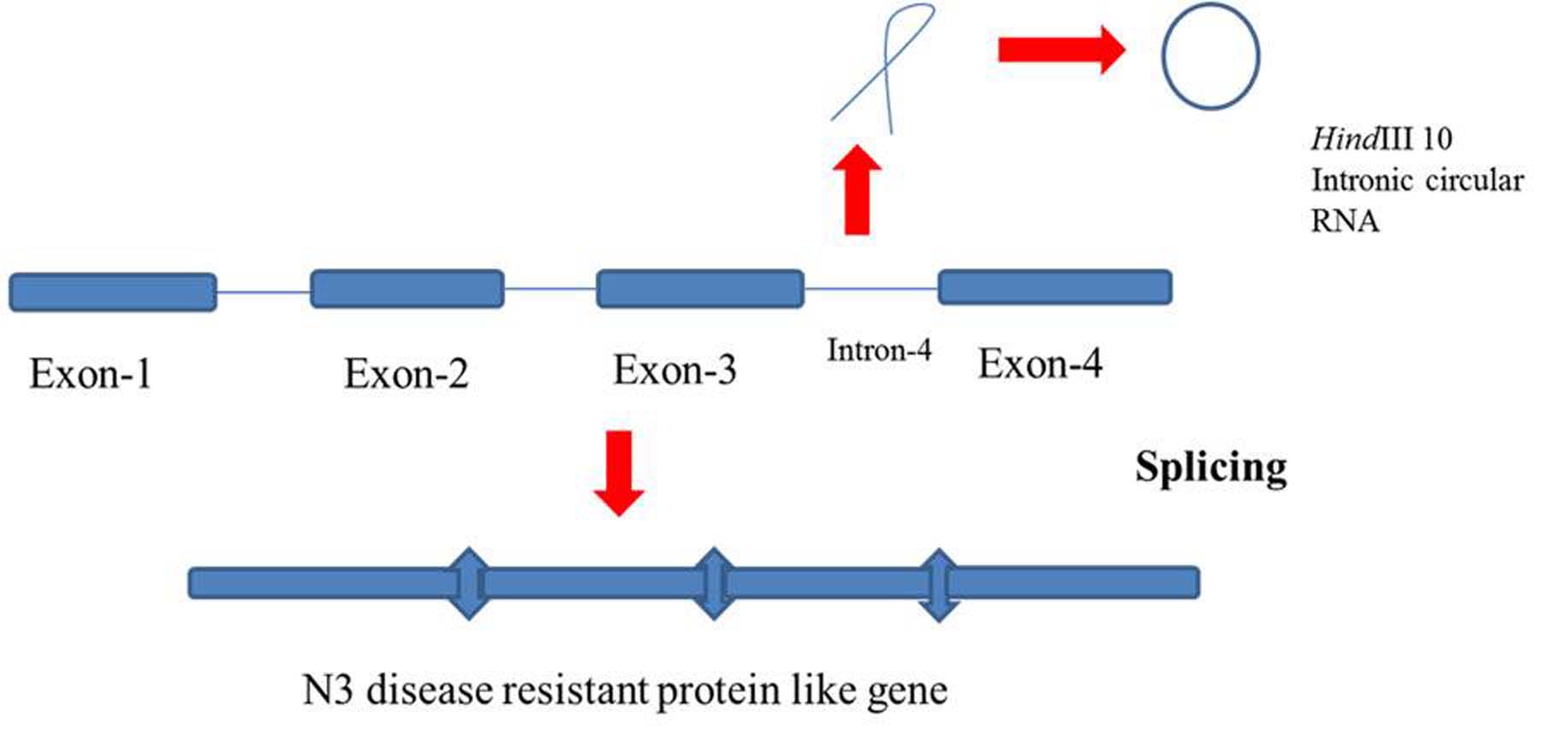


**b**

**
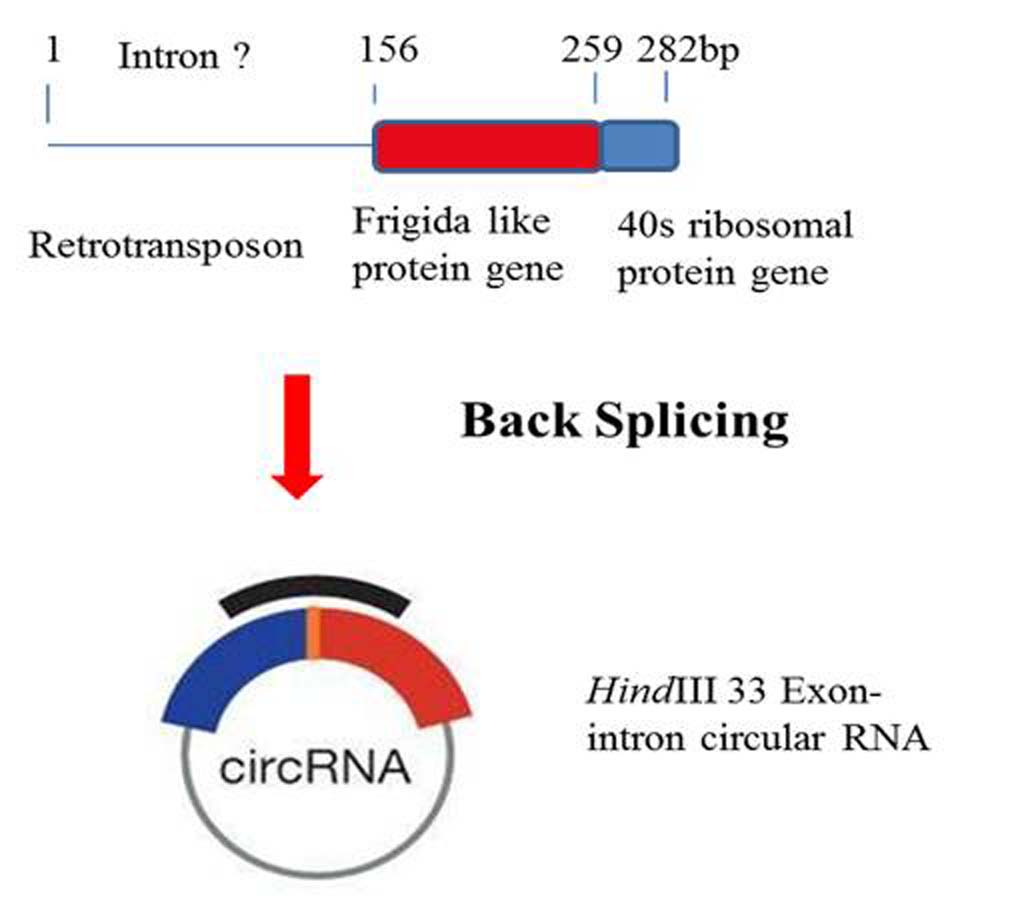
**

**c d**

**
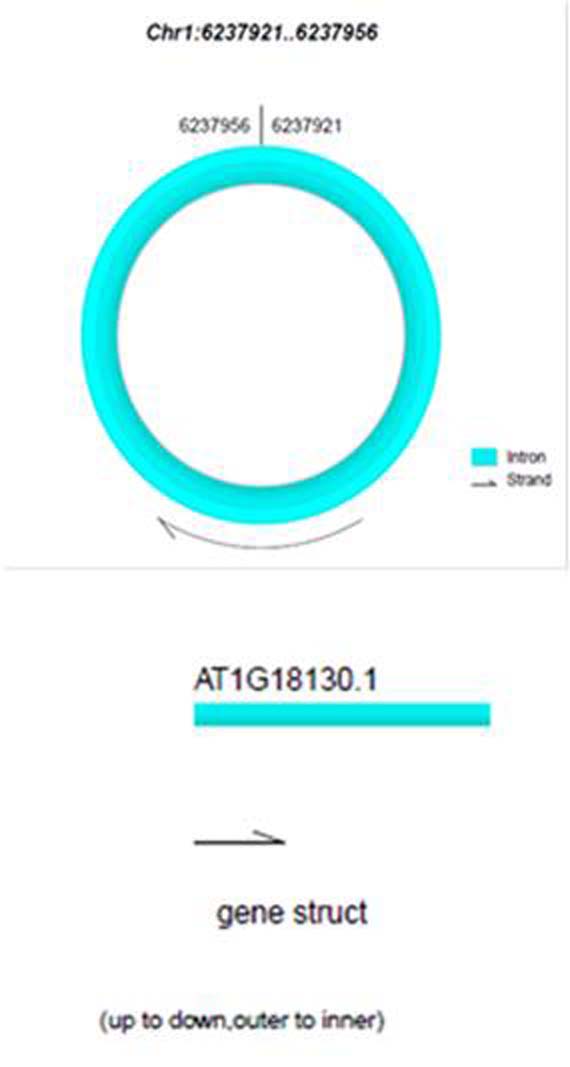
**
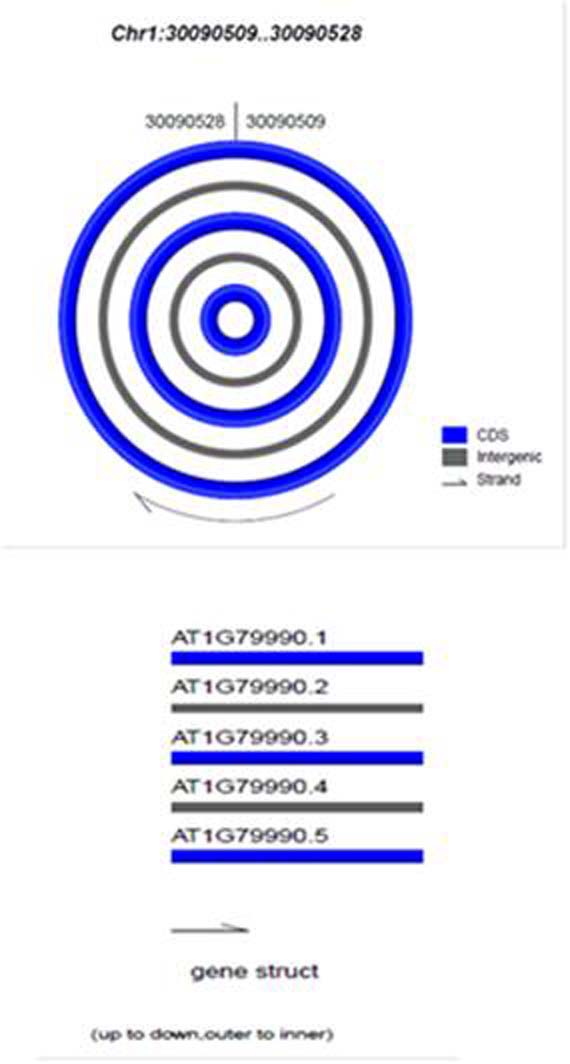


**Figure S3.** **Linear and predicted Circular maps of the putative circRNAs of *N. benthamiana.*** **a** The intronic circRNA, *Hind*III 10 is produced from the intron sequences of N3 disease resistant protein like gene. **b** The possible intronic-exonic circRNA, *Hind*III 33 is partially derived from retrotransposon, Frigida like protein gene and from 40S ribosomal protein gene. Two of the cloned sequences showed the possibility of forming circRNAs when checked with PlantcircBase by mapping with the *Arabidopsis* plant. **c** The clone *Hind*III 10 was predicted to be an intronic circRNA. **d** The clone *Hind*III 33 was predicted to be an intronic-exonic circRNAs.

**a b**

**
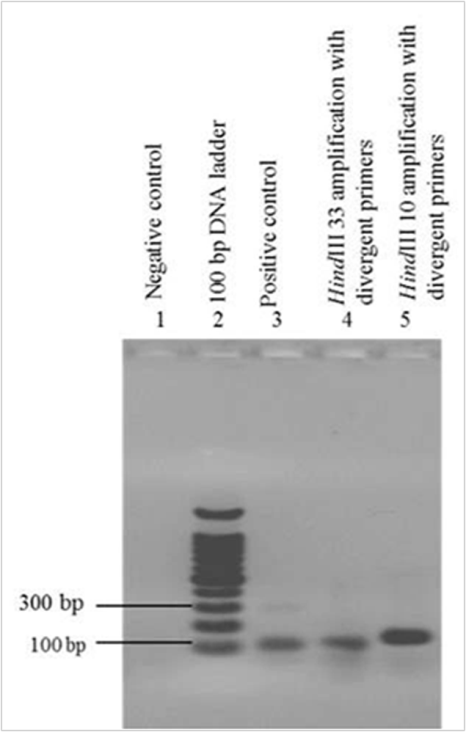

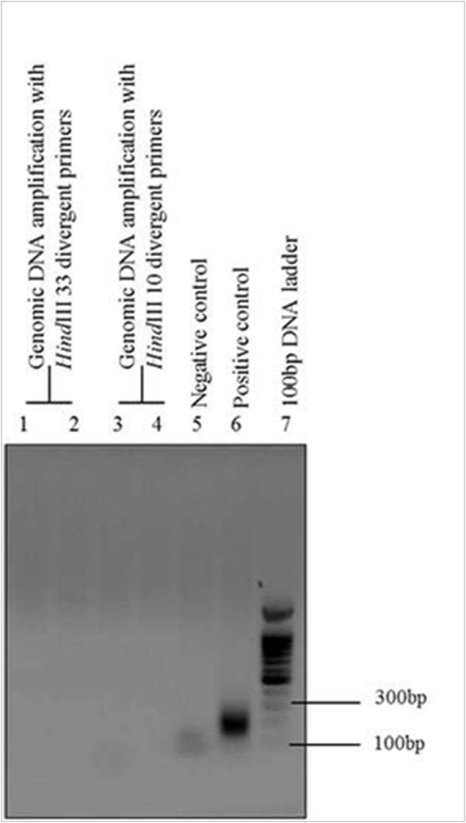
**

**Figure S4. Divergent PCR for circRNA Confirmation.** **a** The two clones (*Hind*III 10 and *Hind*III 33) of *N. benthamiana* are predicted as possible putative circRNAs by PlantcircBase. To confirm their circular nature, divergent primers were designed and PCR was performed for *Hind*III 10 (lane 5) and *Hind*III 33 (lane 4) along with negative (lane 1) and PCR positive controls (18srRNA, lane 3). Lane 2 is 100bp DNA ladder. **b** Divergent PCR with genomic DNA yielded no amplification (lane1, 2 for *Hind*III 33 primer) (lane 3, 4 for *Hind*III 10 primer) along with amplification in positive control (lane 6) and nil amplification in negative control (lane 5) confirming the circRNA detection. Lane 7 is 100bp DNA ladder.

**a *Sac*I 11**

**
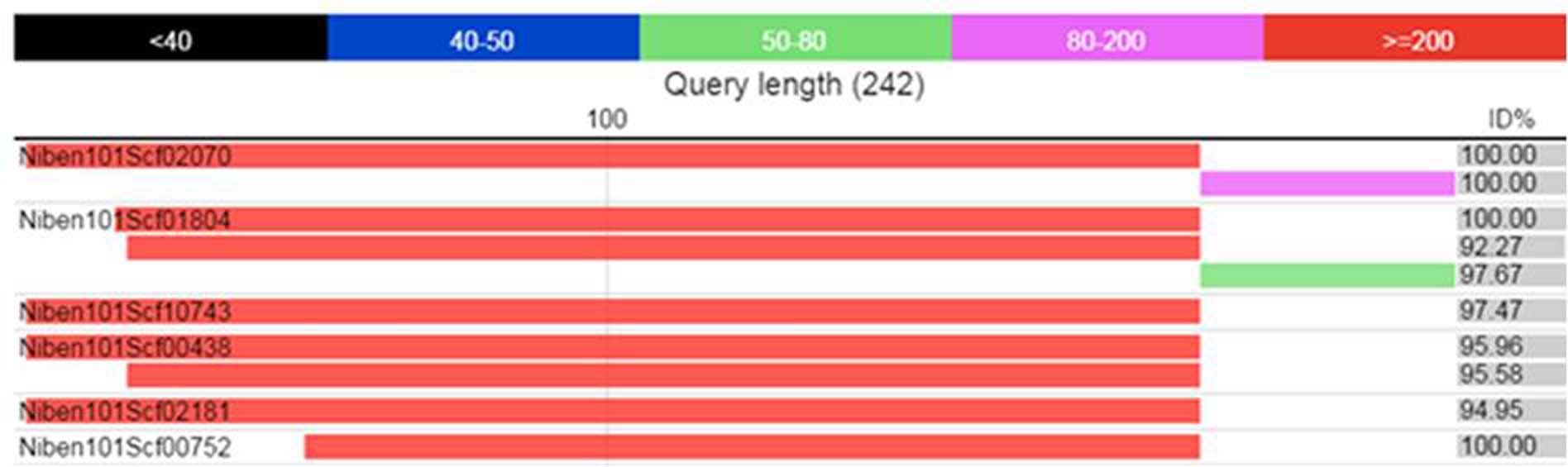
**

**
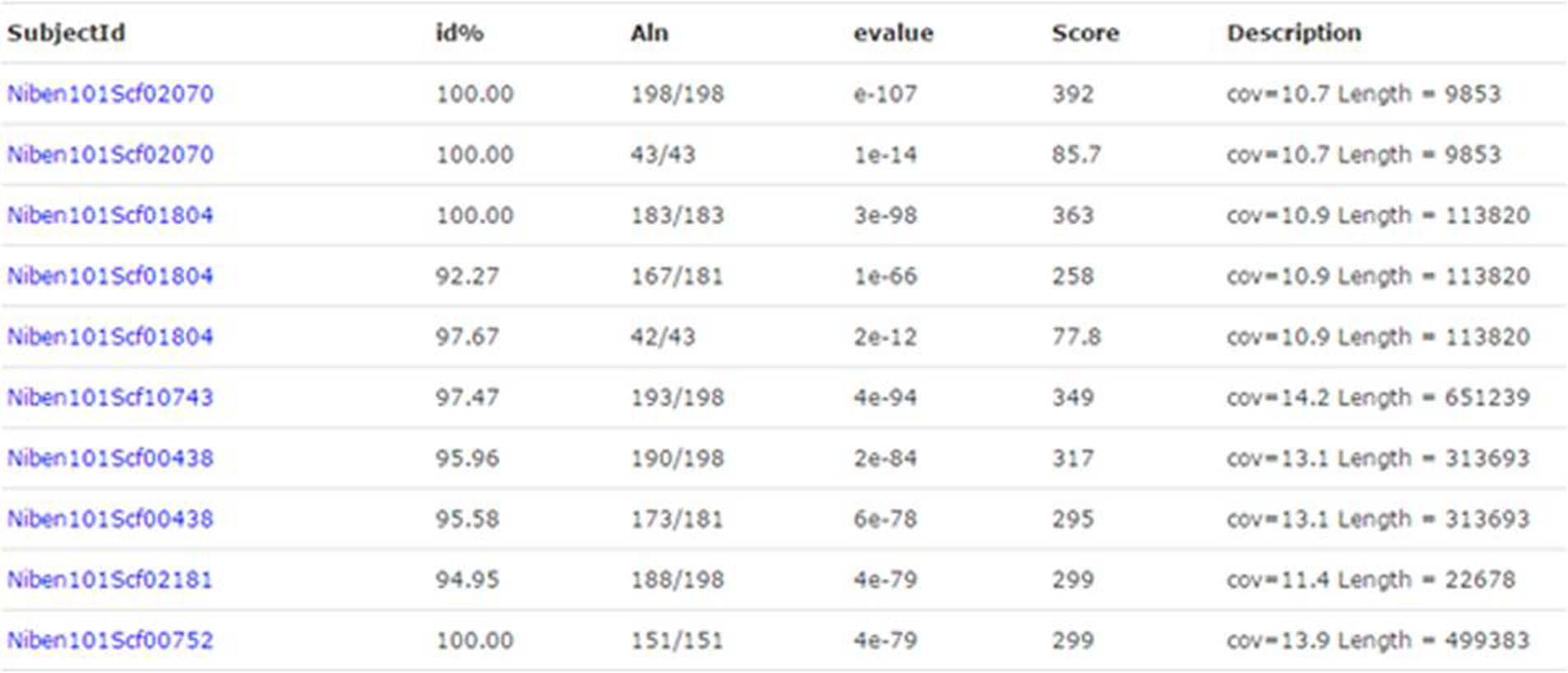
**

**
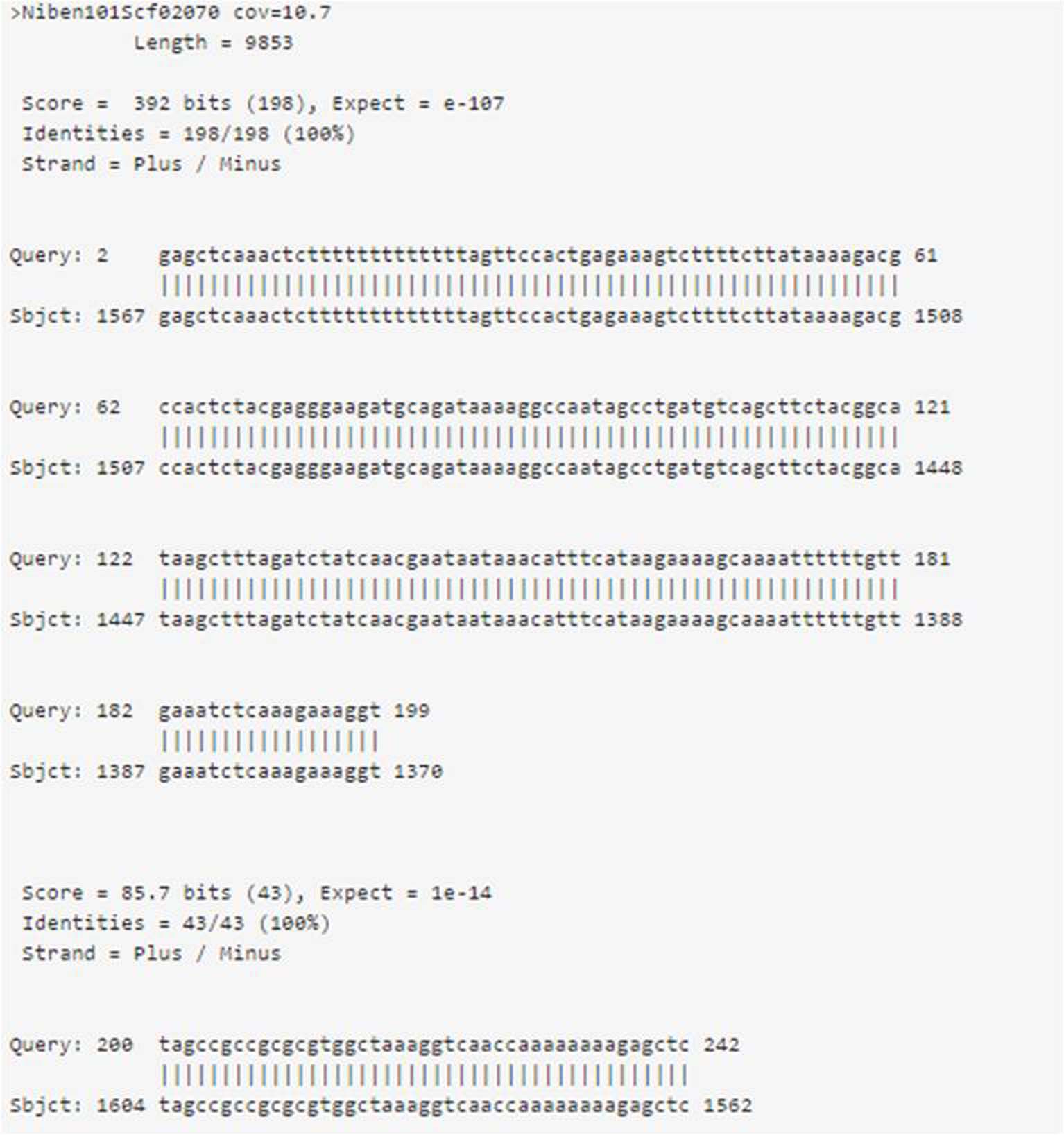
**

**b *Hind*III 10**

**
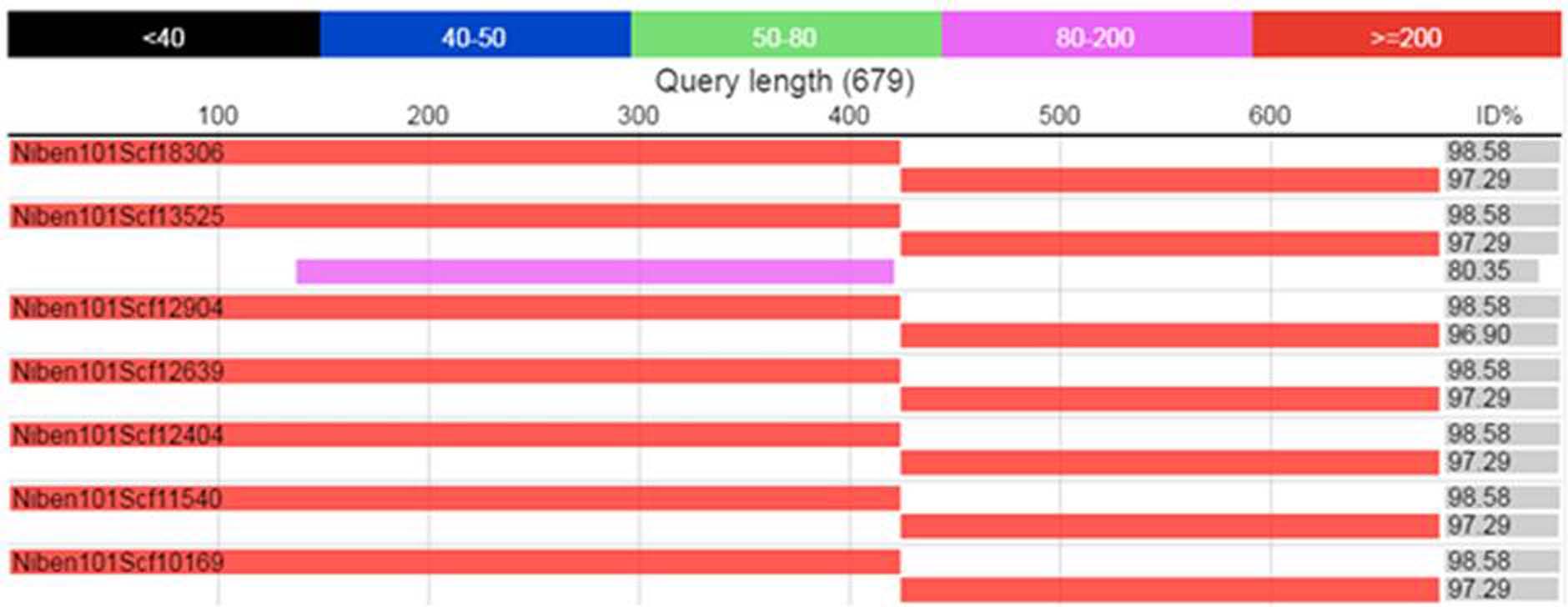
**

**
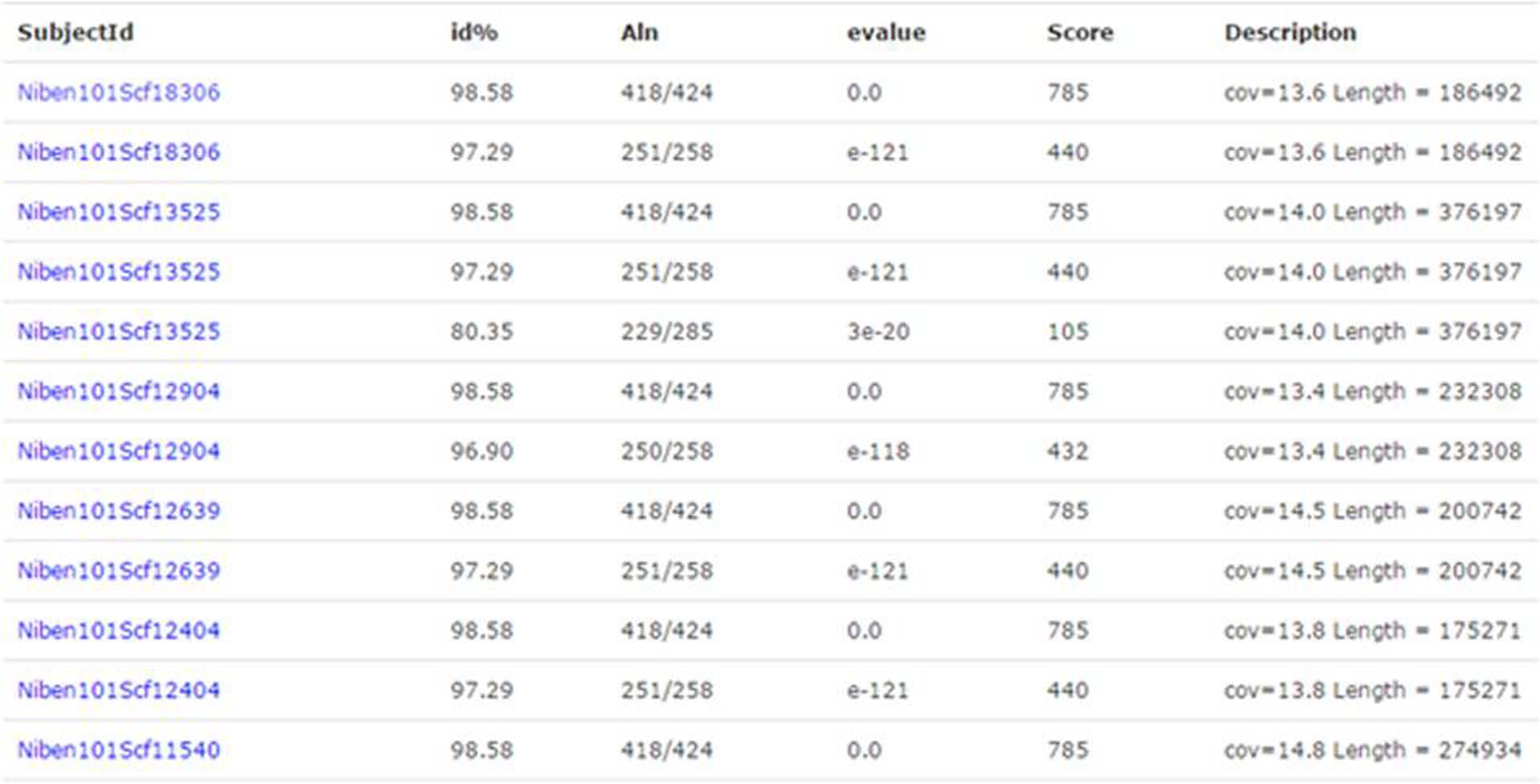
**

**
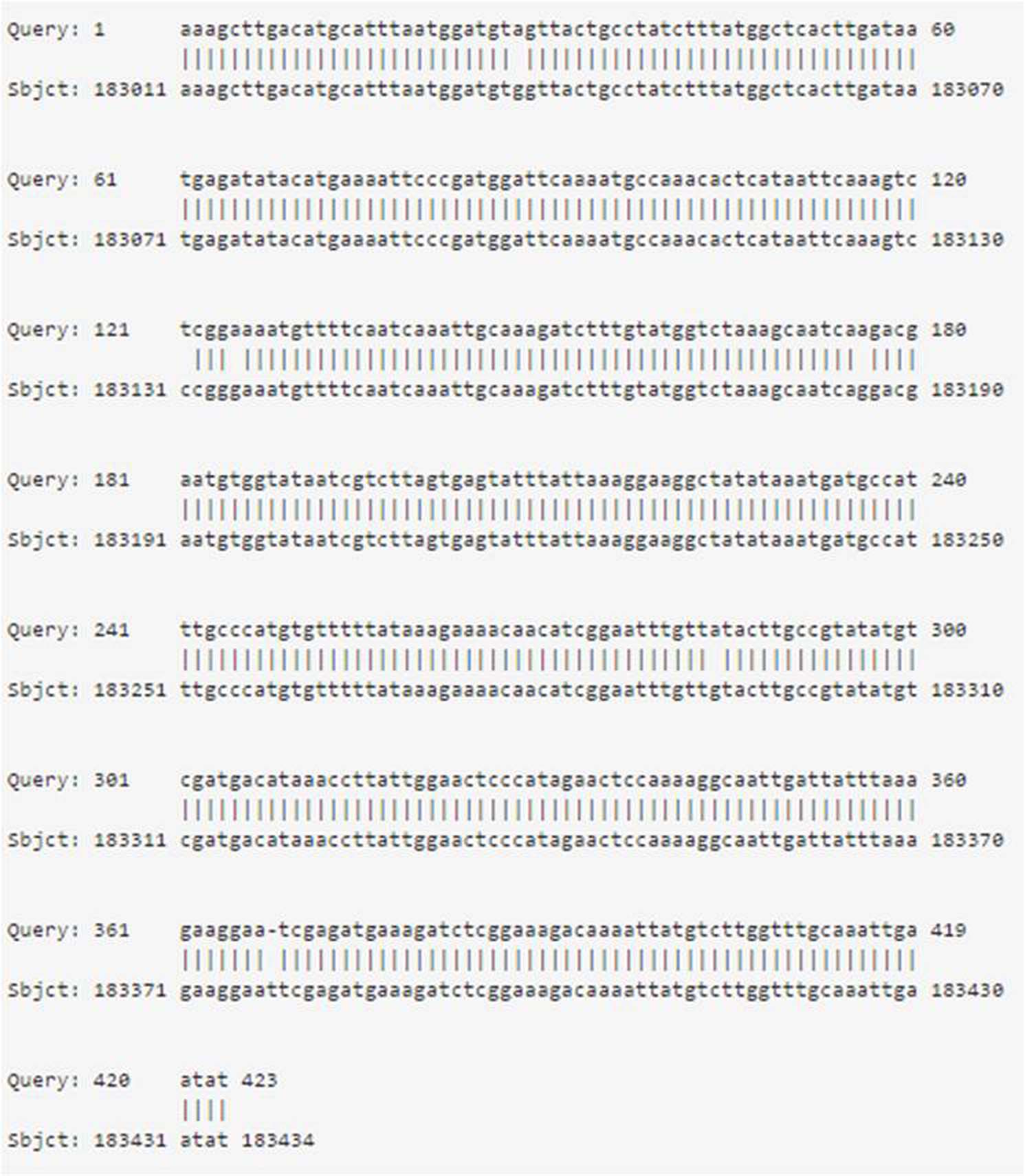
**

**
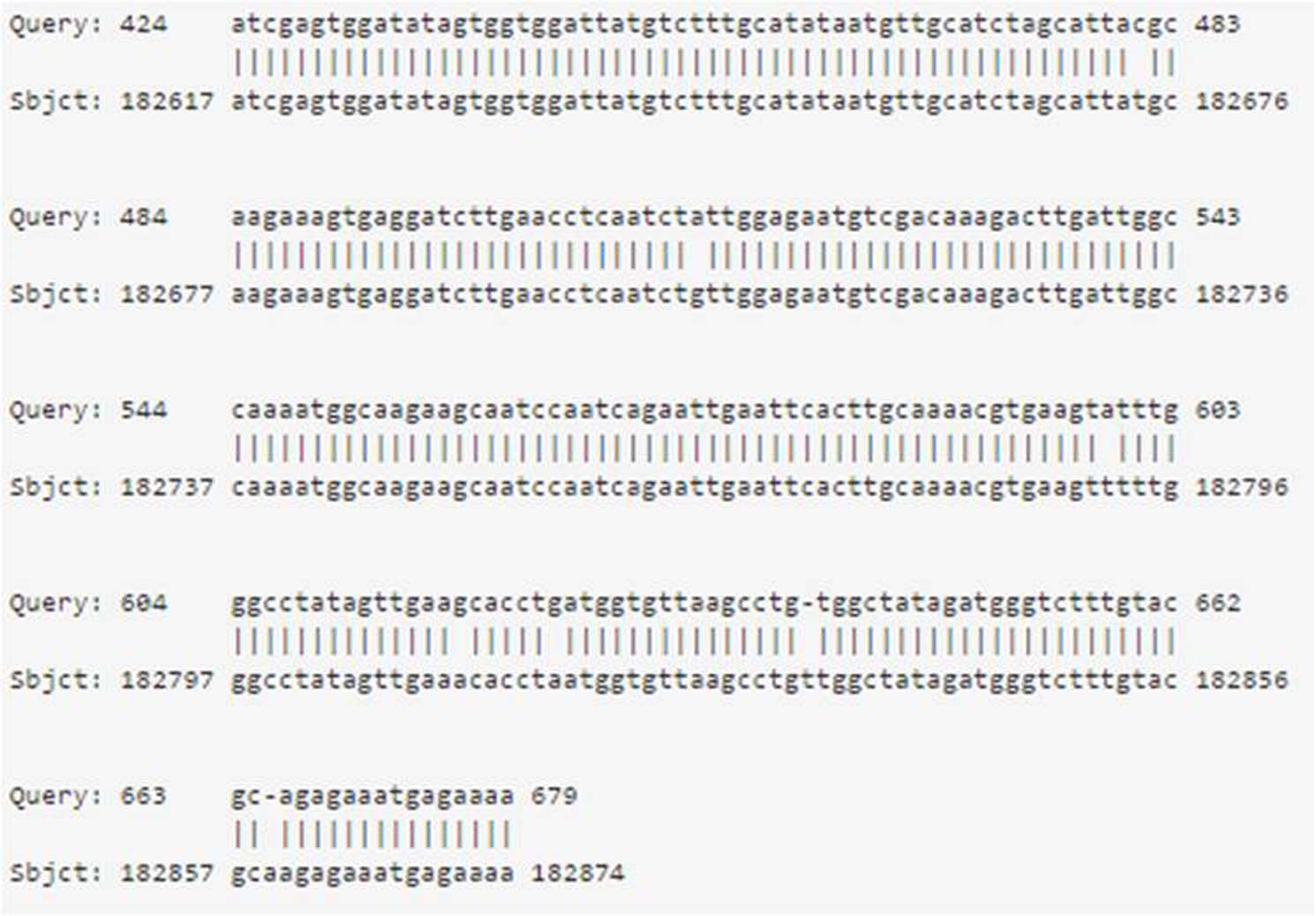
**

**c *Hind*III 33**

**
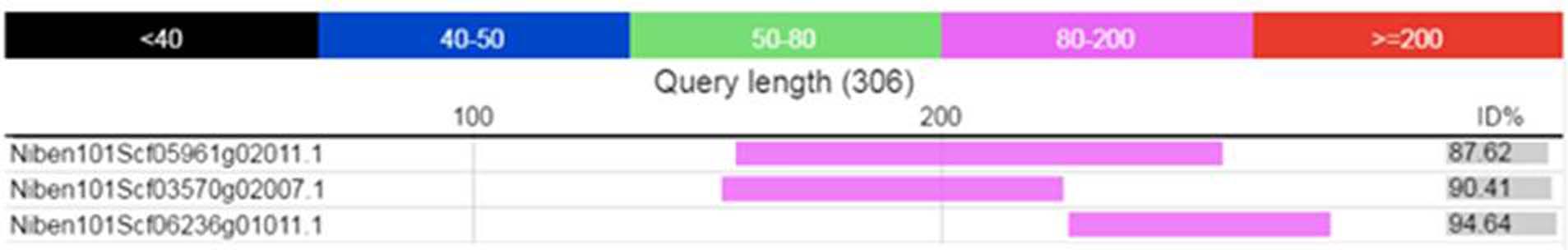
**

**
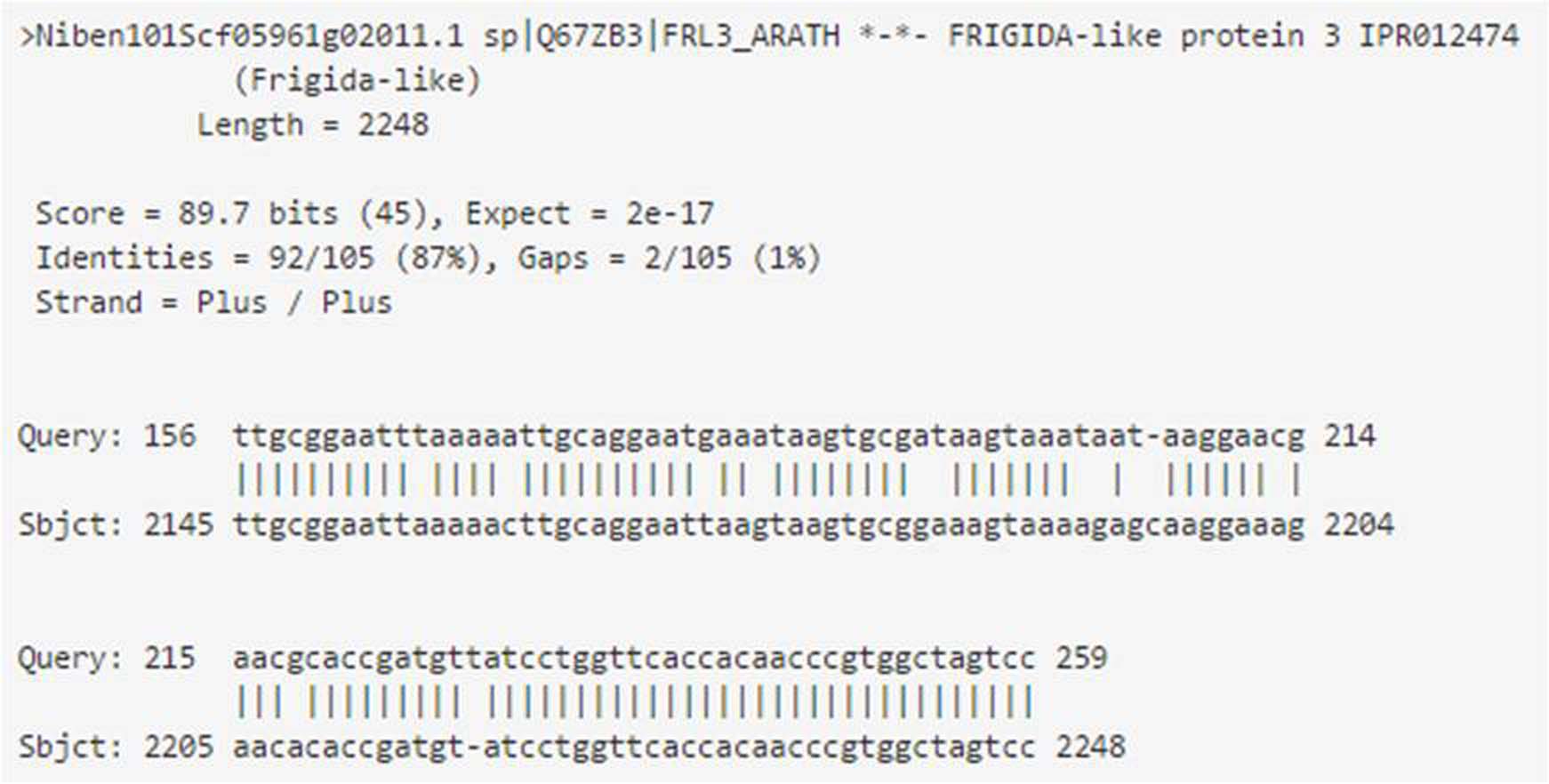
**

**d *Hind*III 33**

**
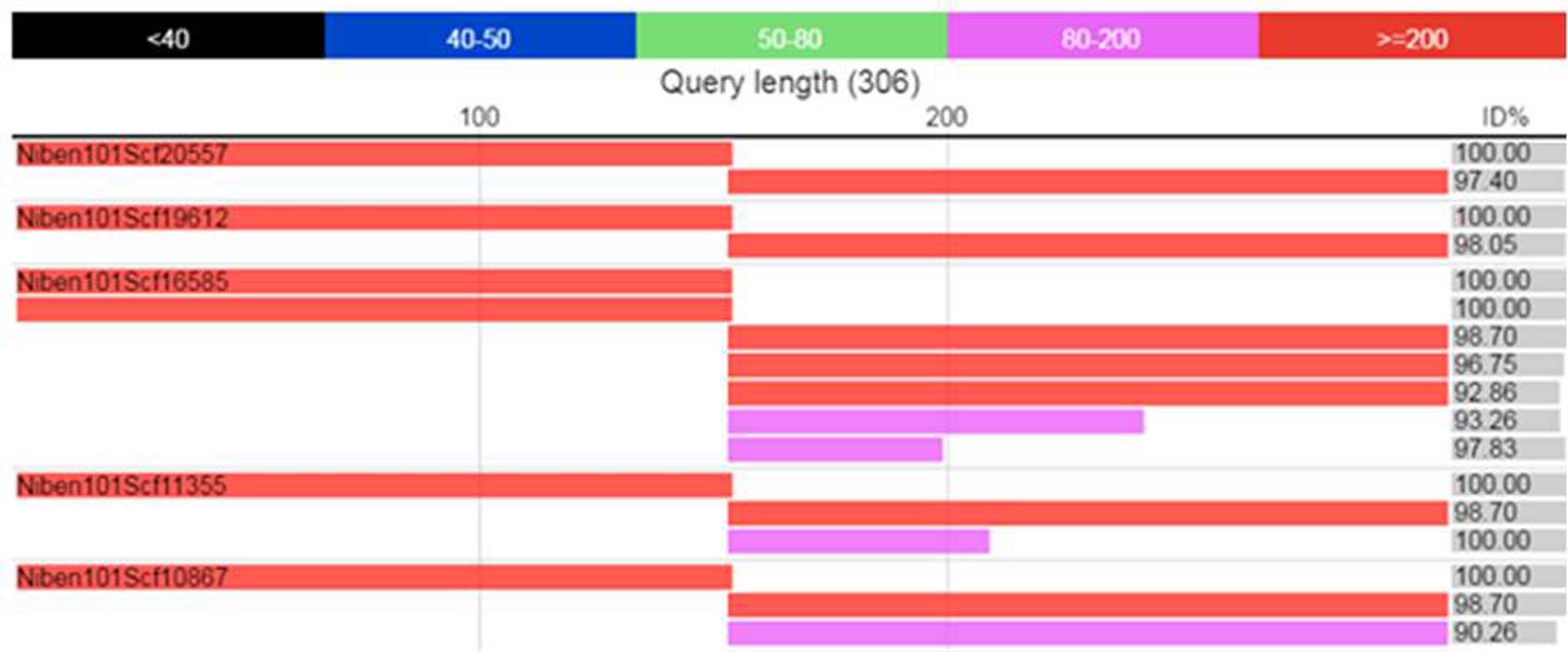
**

**
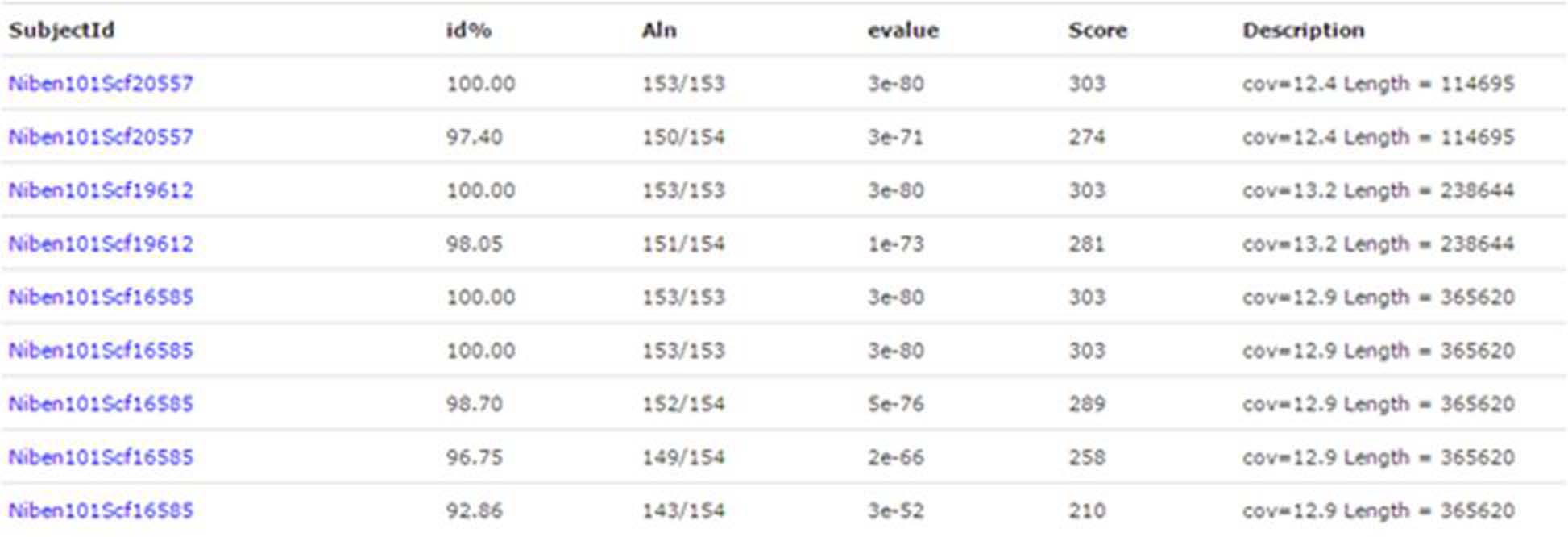
**

**
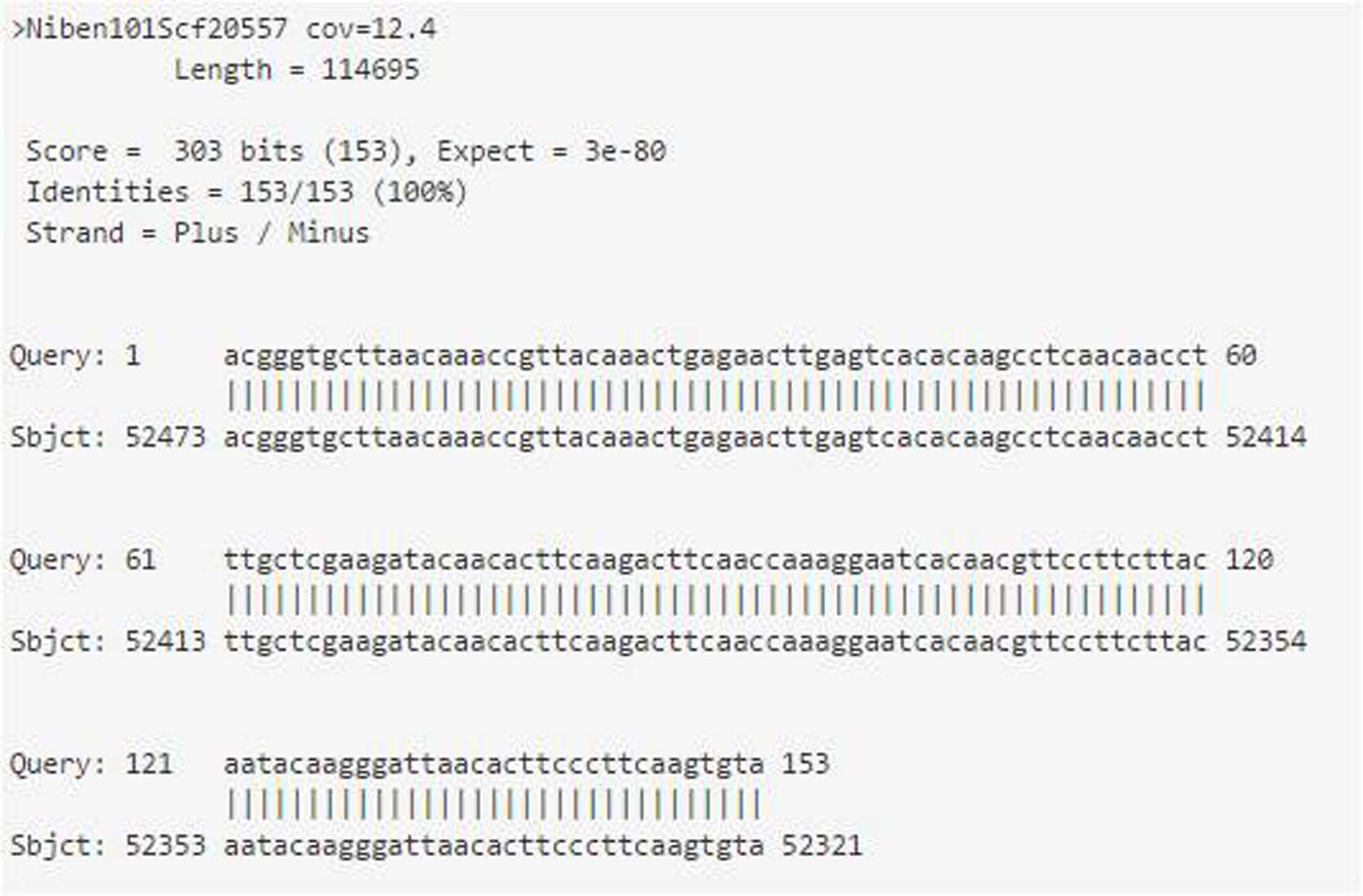
**

**
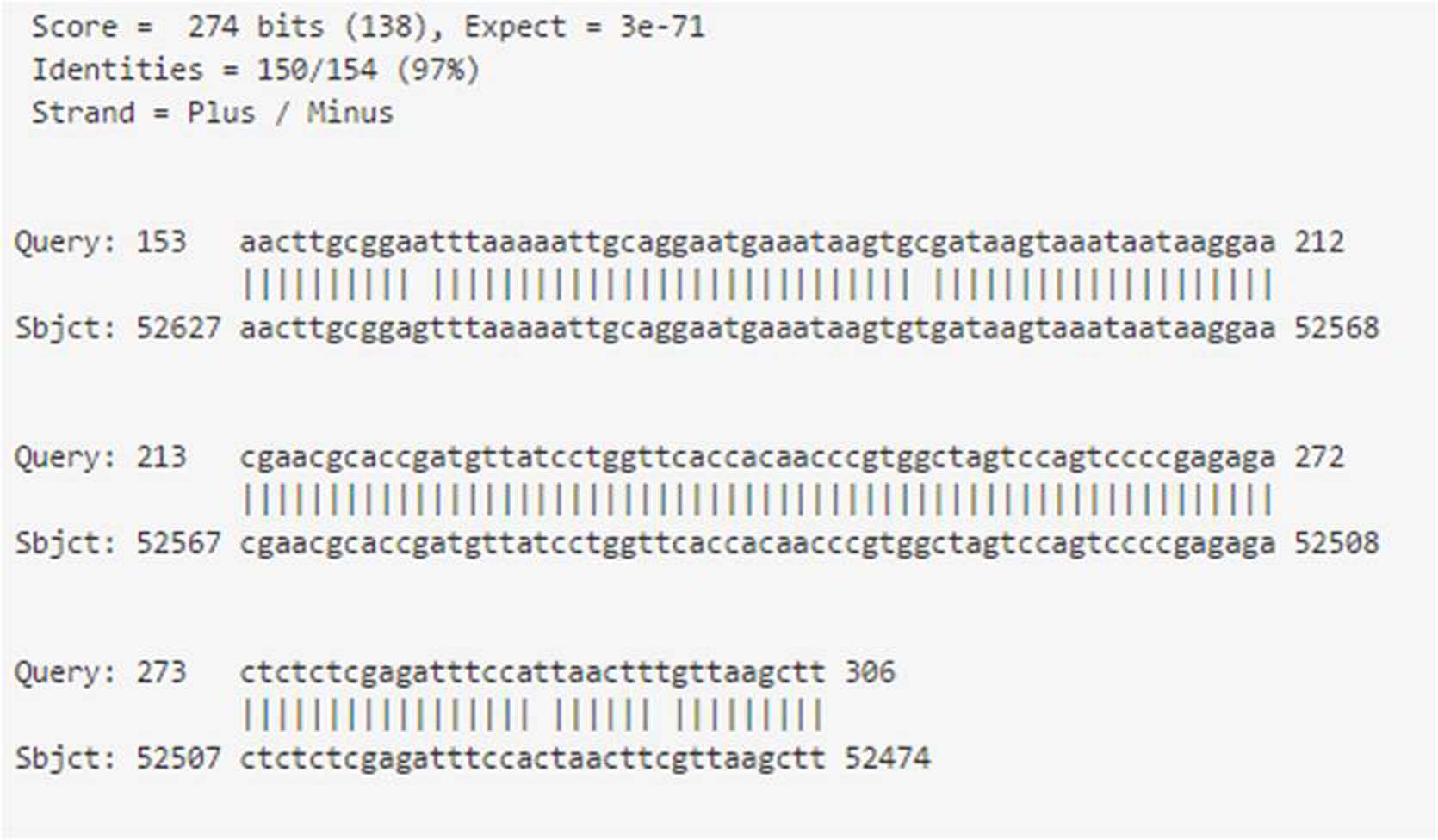
**

**e *Hind*III 38**

**
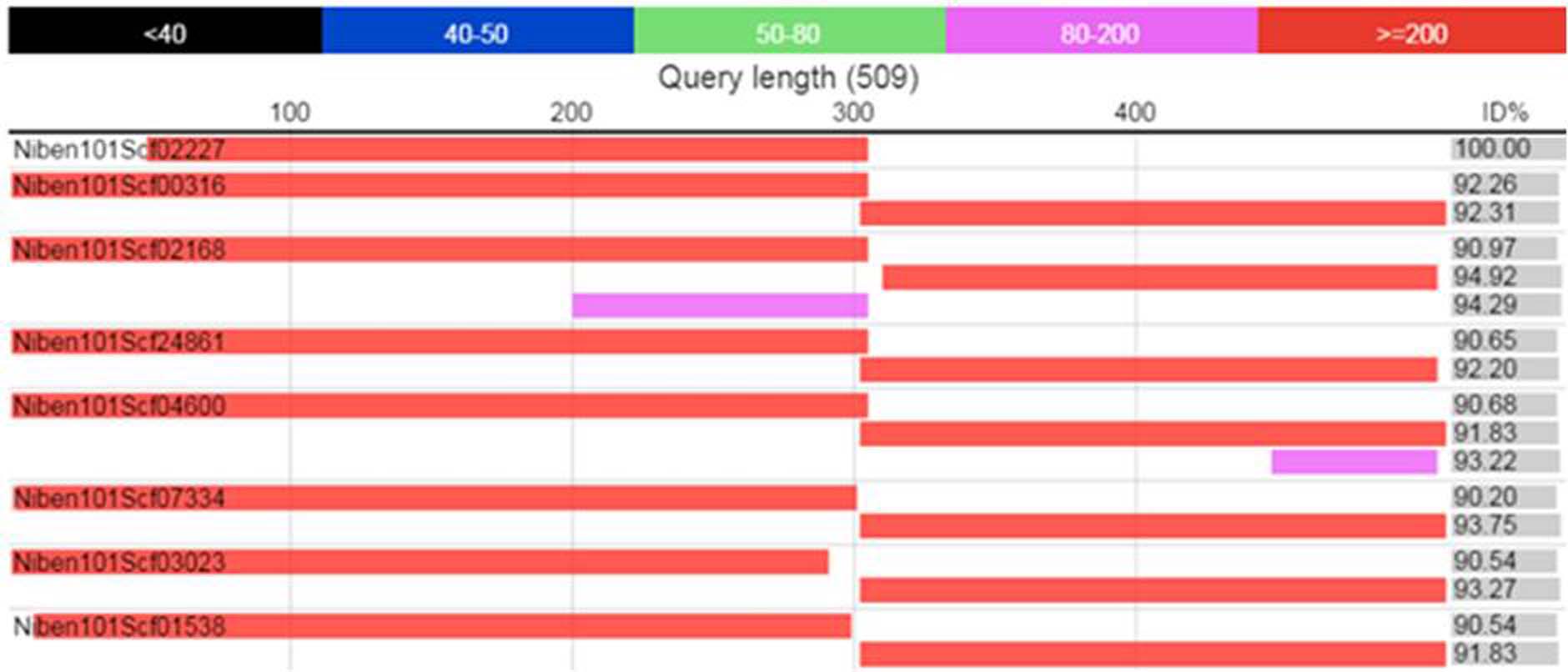
**

**
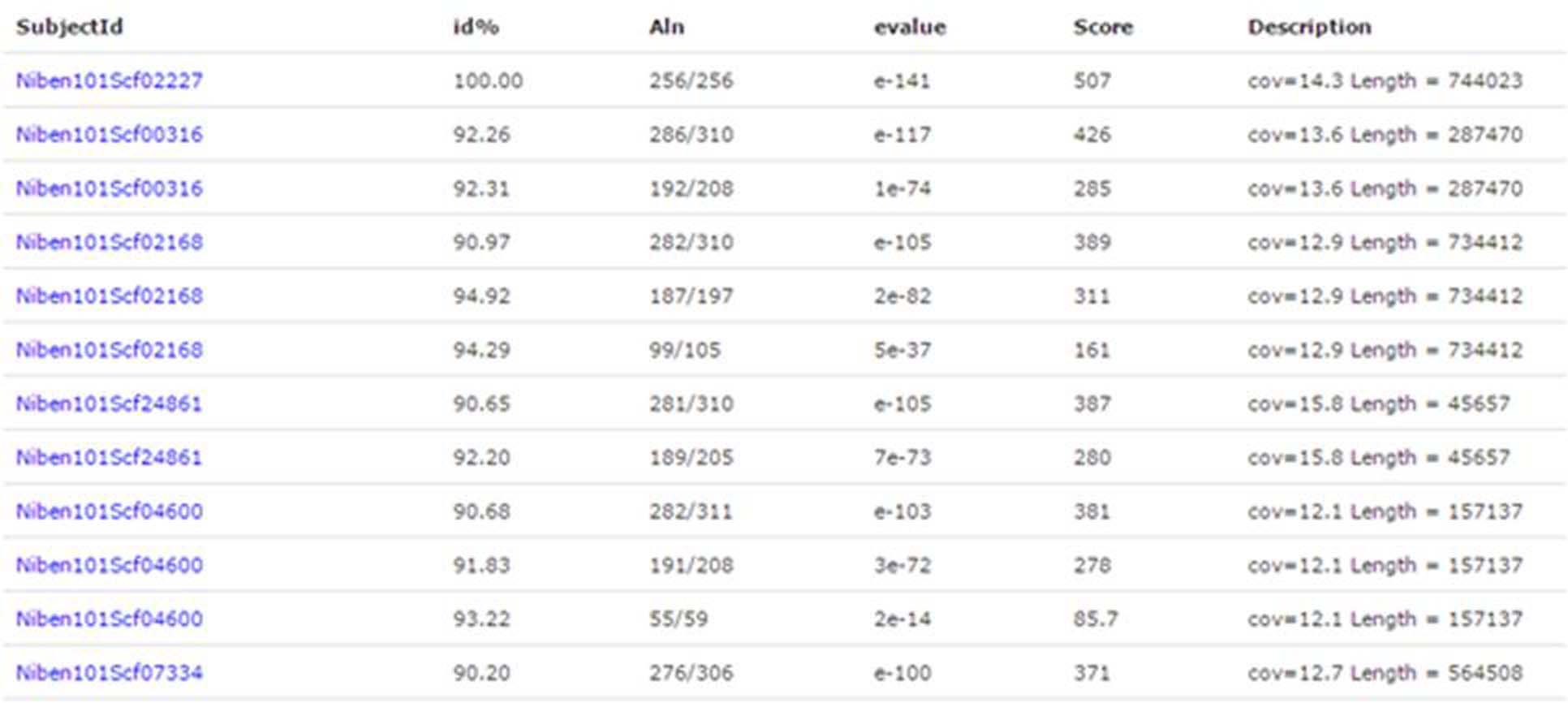
**

**
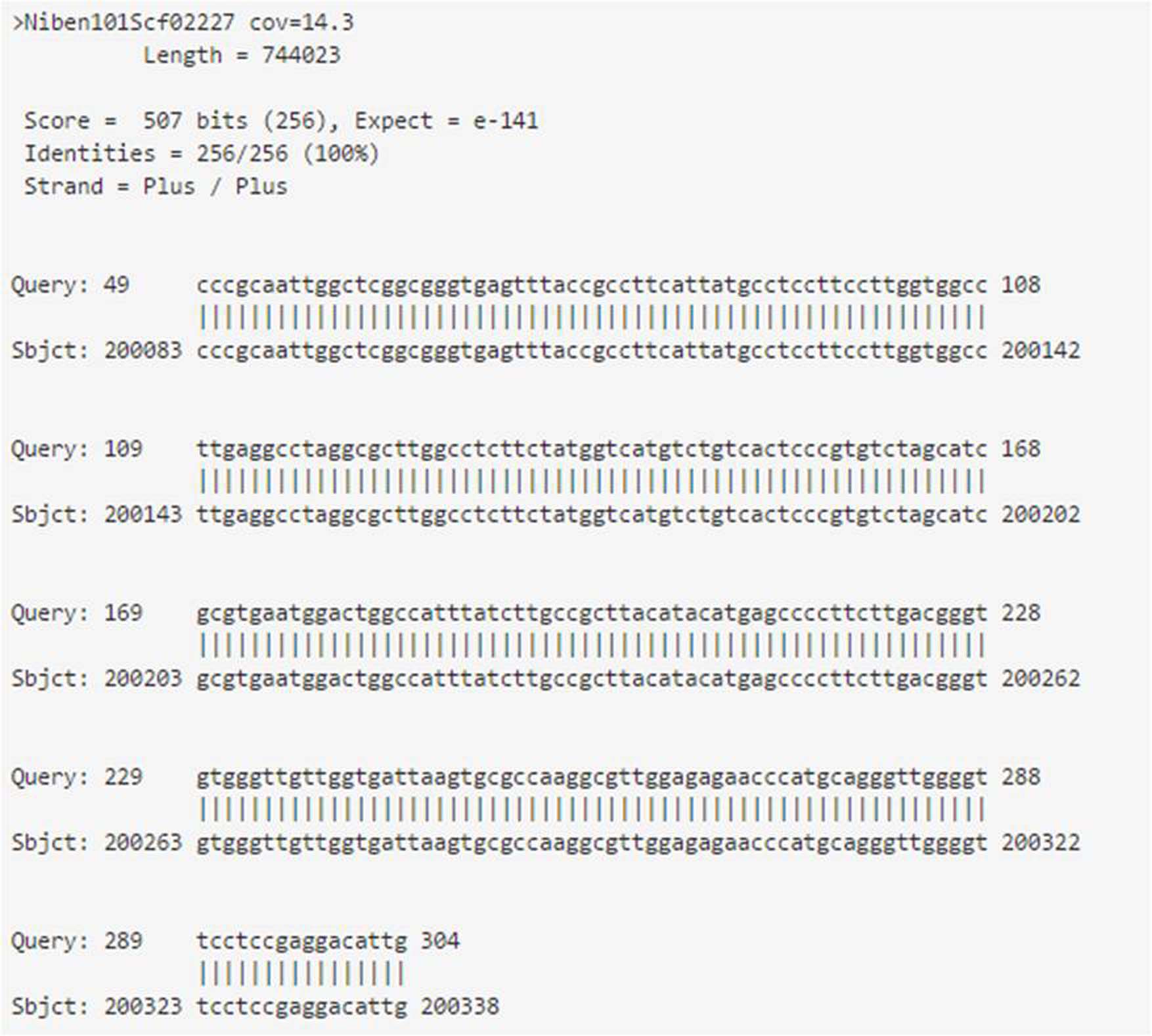
**

**Figure S5. BLAST Analysis of *N. benthamiana* cloned sequences in Sol Genomics network.** *Hind*III and *Sac*I digested RCA products of *N. benthamiana* are cloned and sequenced. The sequences were subjected to BLAST analysis in Sol Genomics Network against predicted cDNA sequences (**c**) and scaffolds and Nr contigs (**a, b, d, e**) of *N. benthamiana*. The sequences showing alignment with the *N. benthamiana* sequences are *Hind*III 10 (**b**), *Hind*III 33 (**c** and **d**), *Hind*III 38 (**e**) and *Sac*I 11 (**a**).

**a *Sac*I 11**


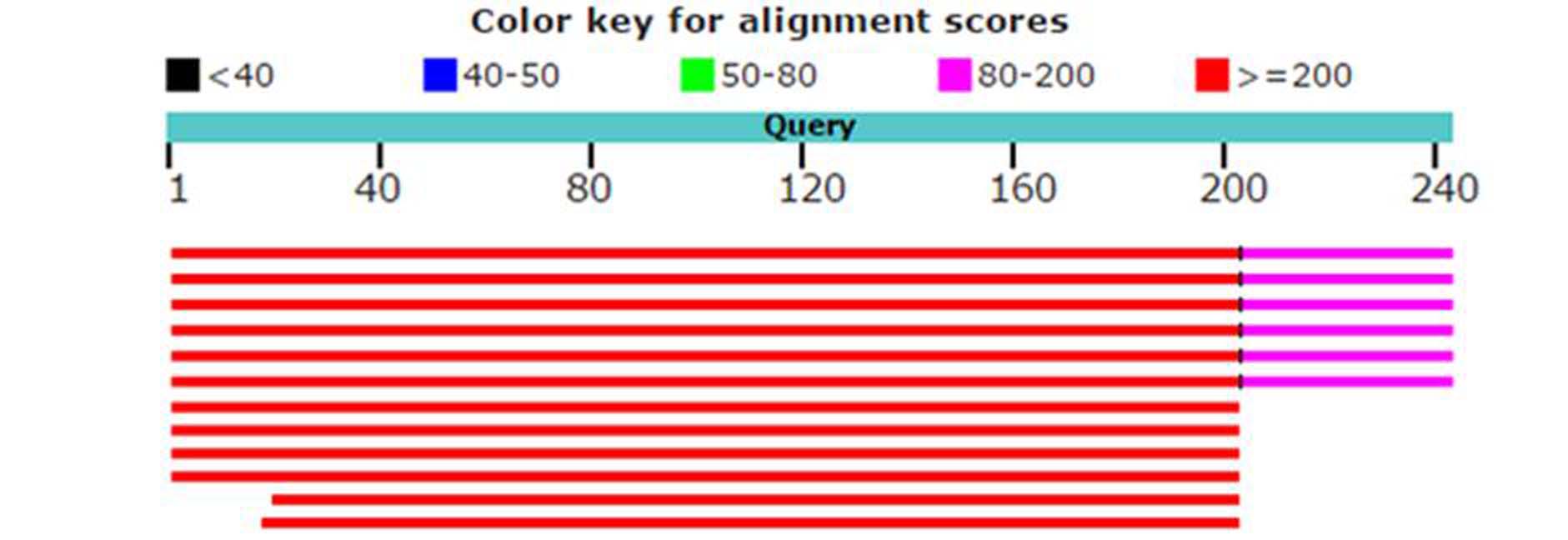


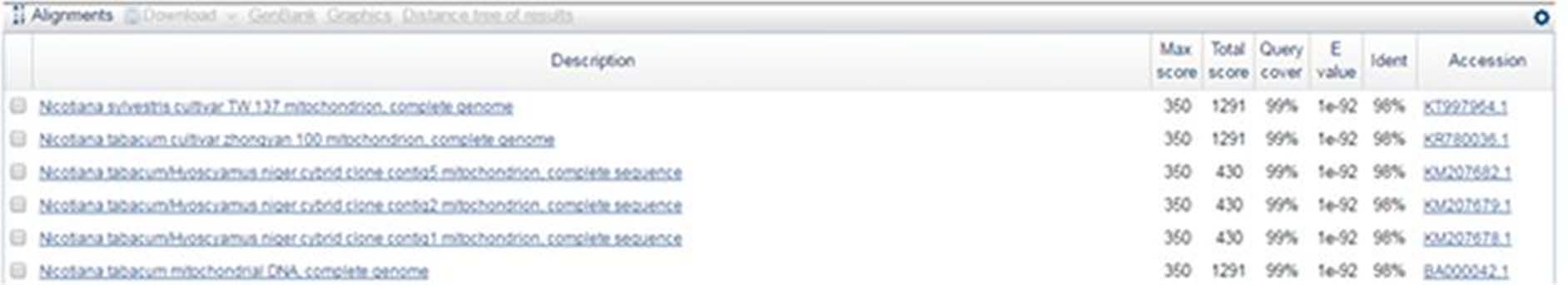


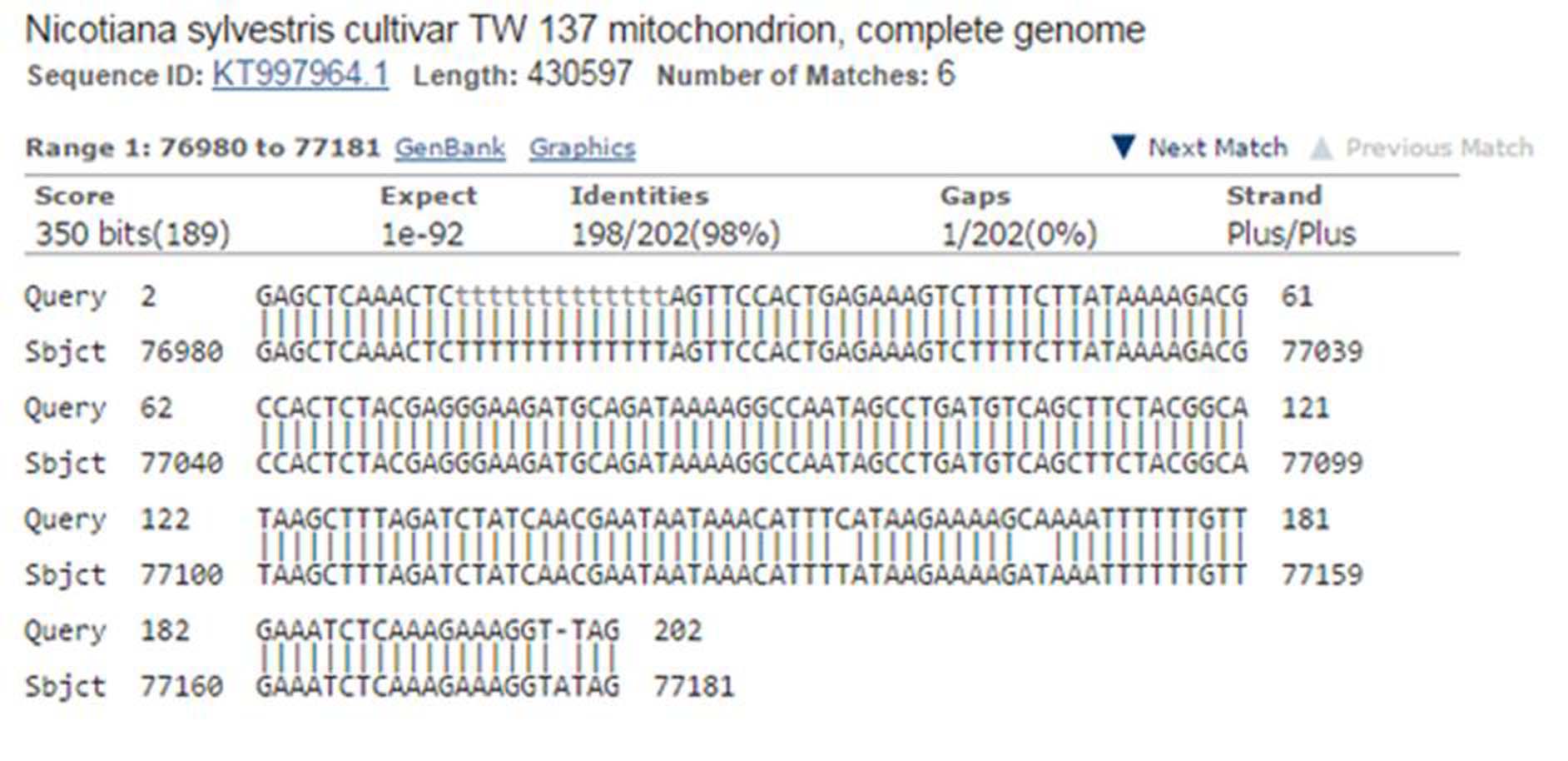


**b *Hind*III 10**


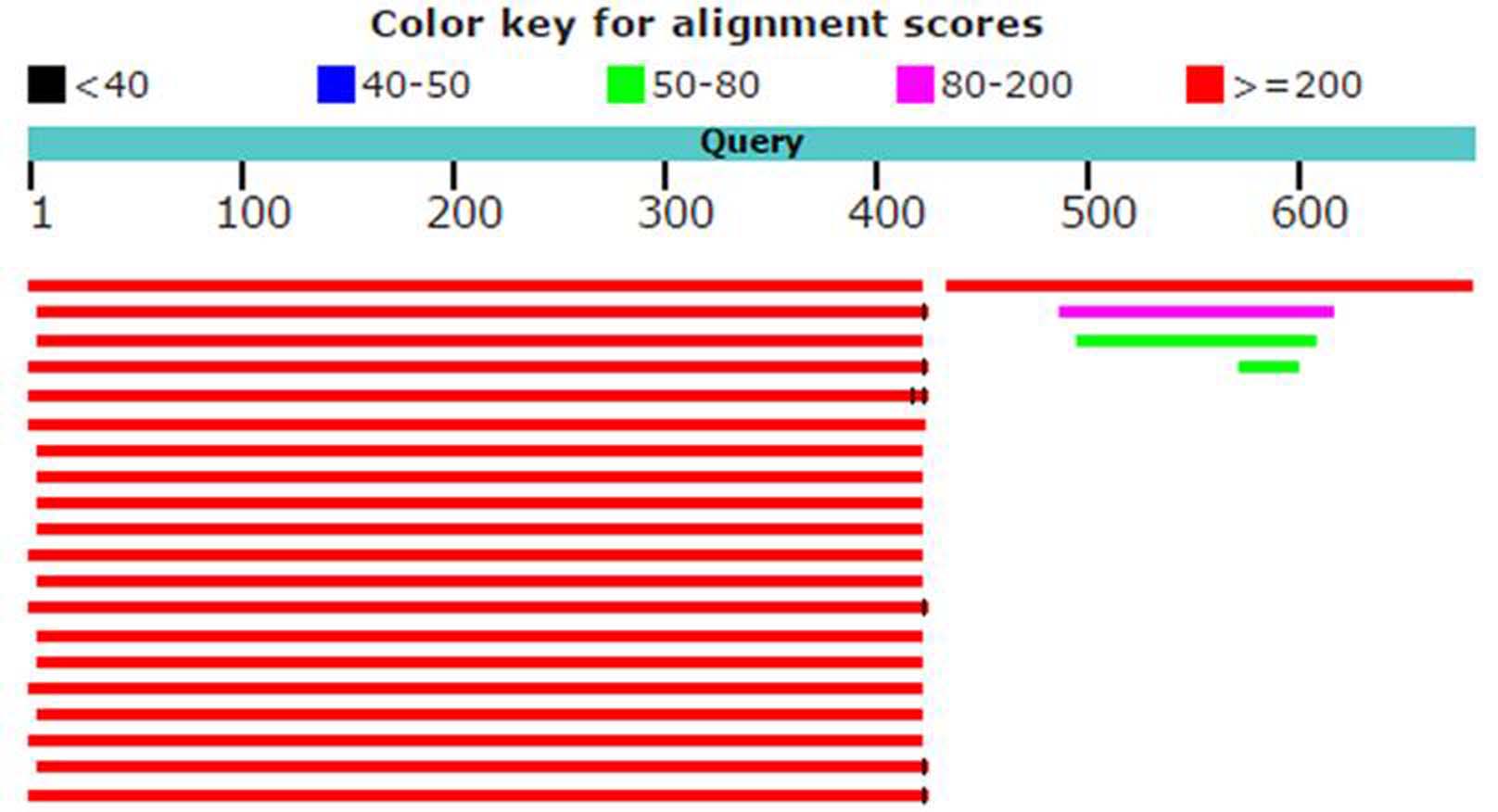


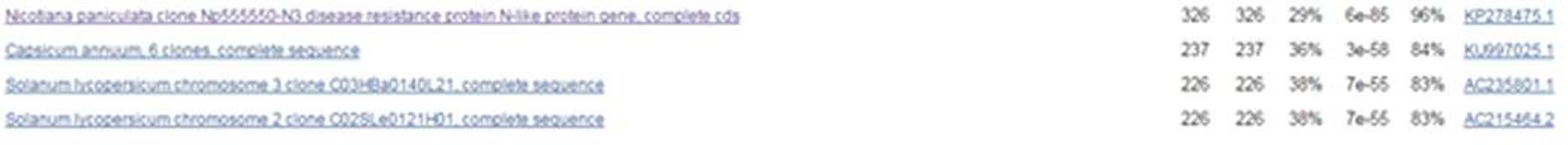


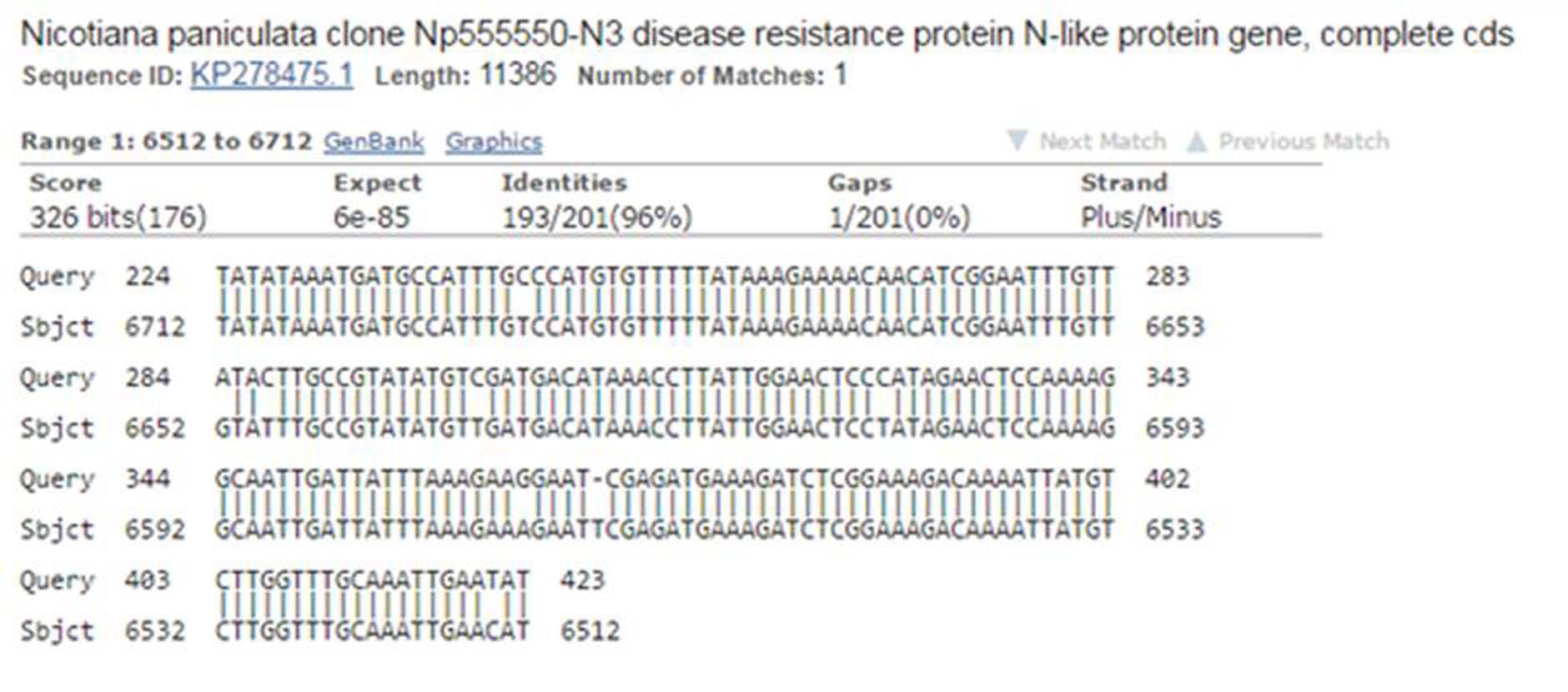


**c *Hind*III 33**


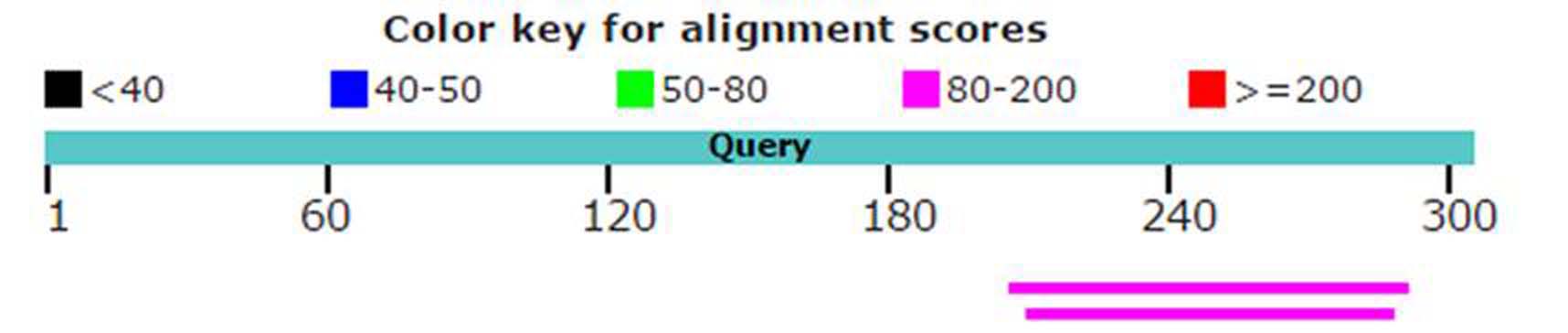


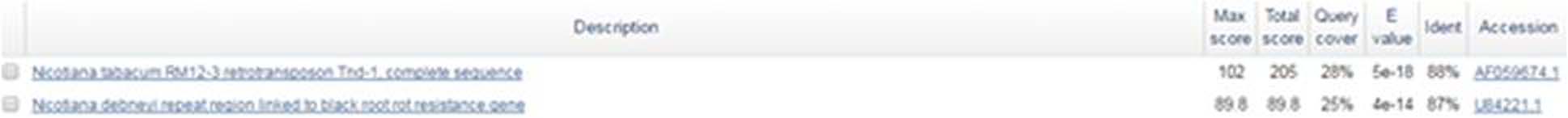


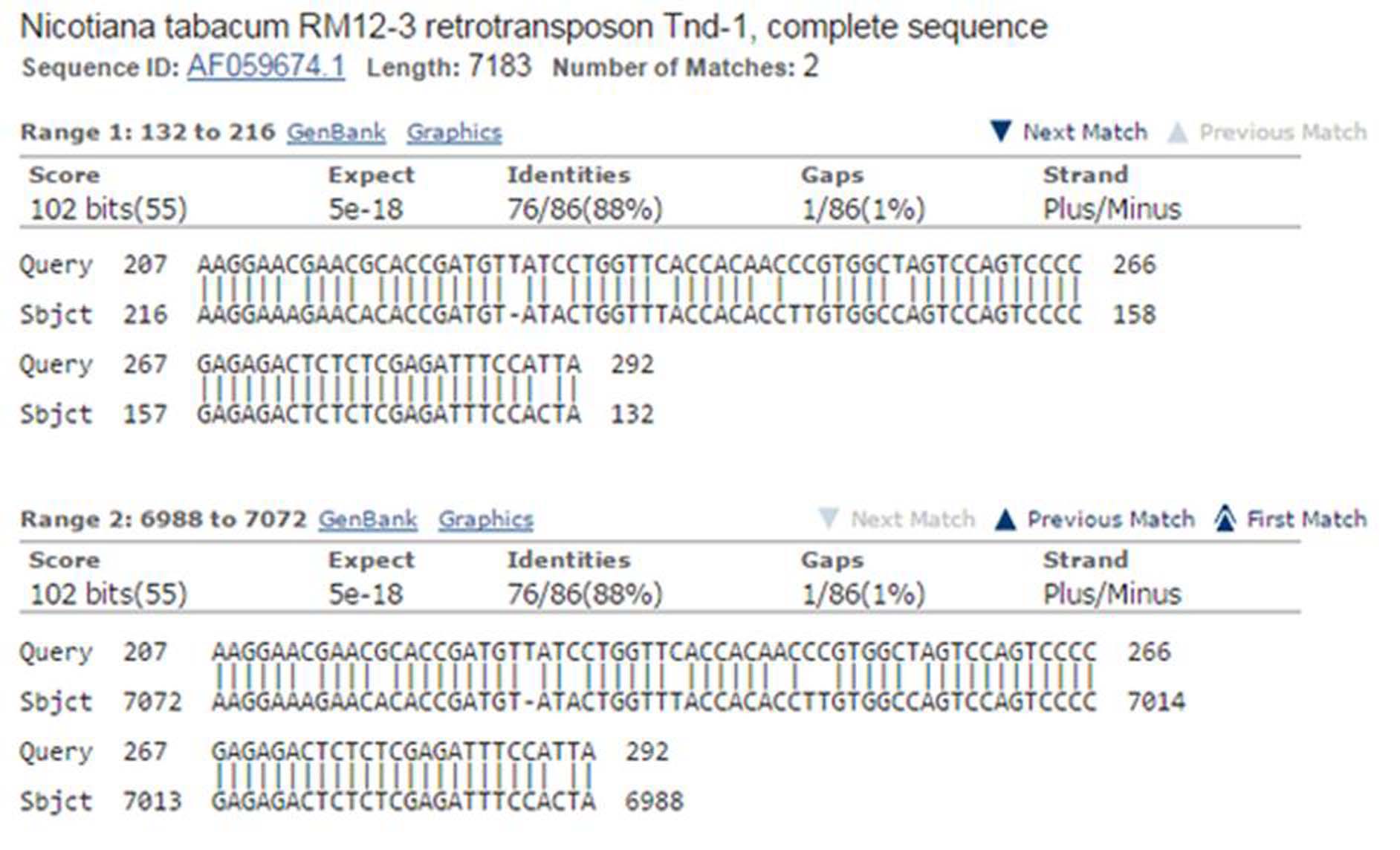


**d *Hind*III 38**


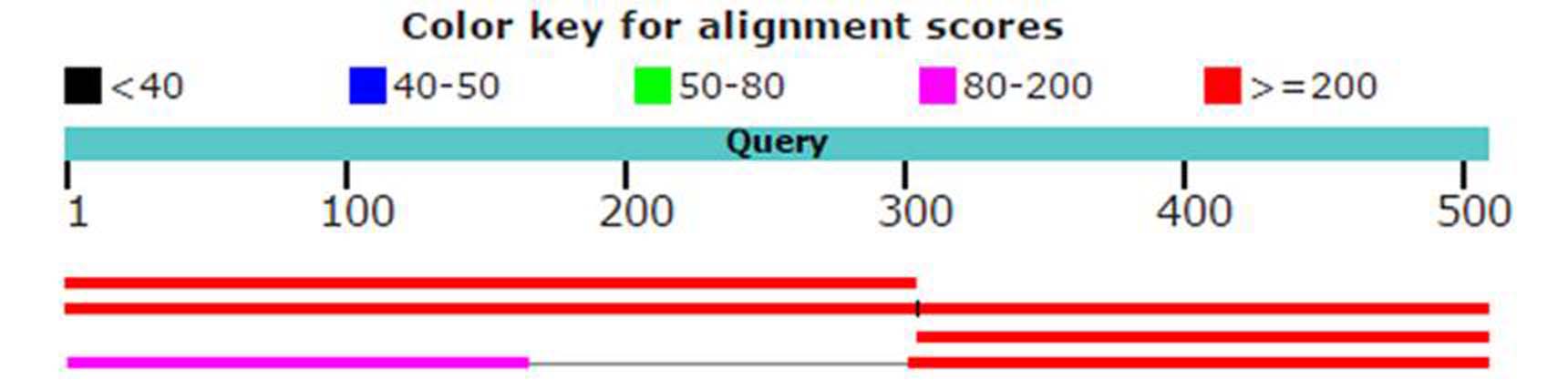


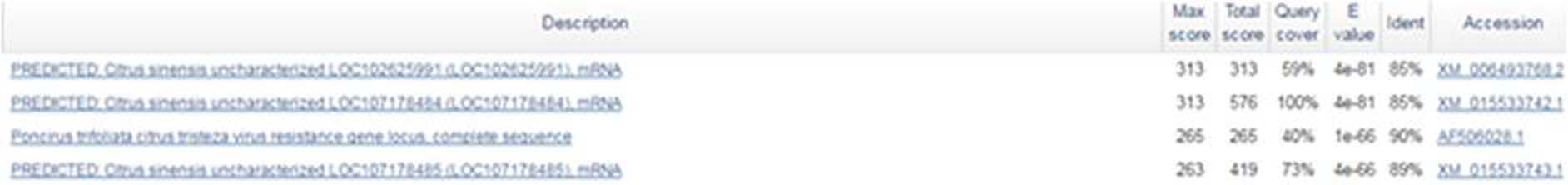


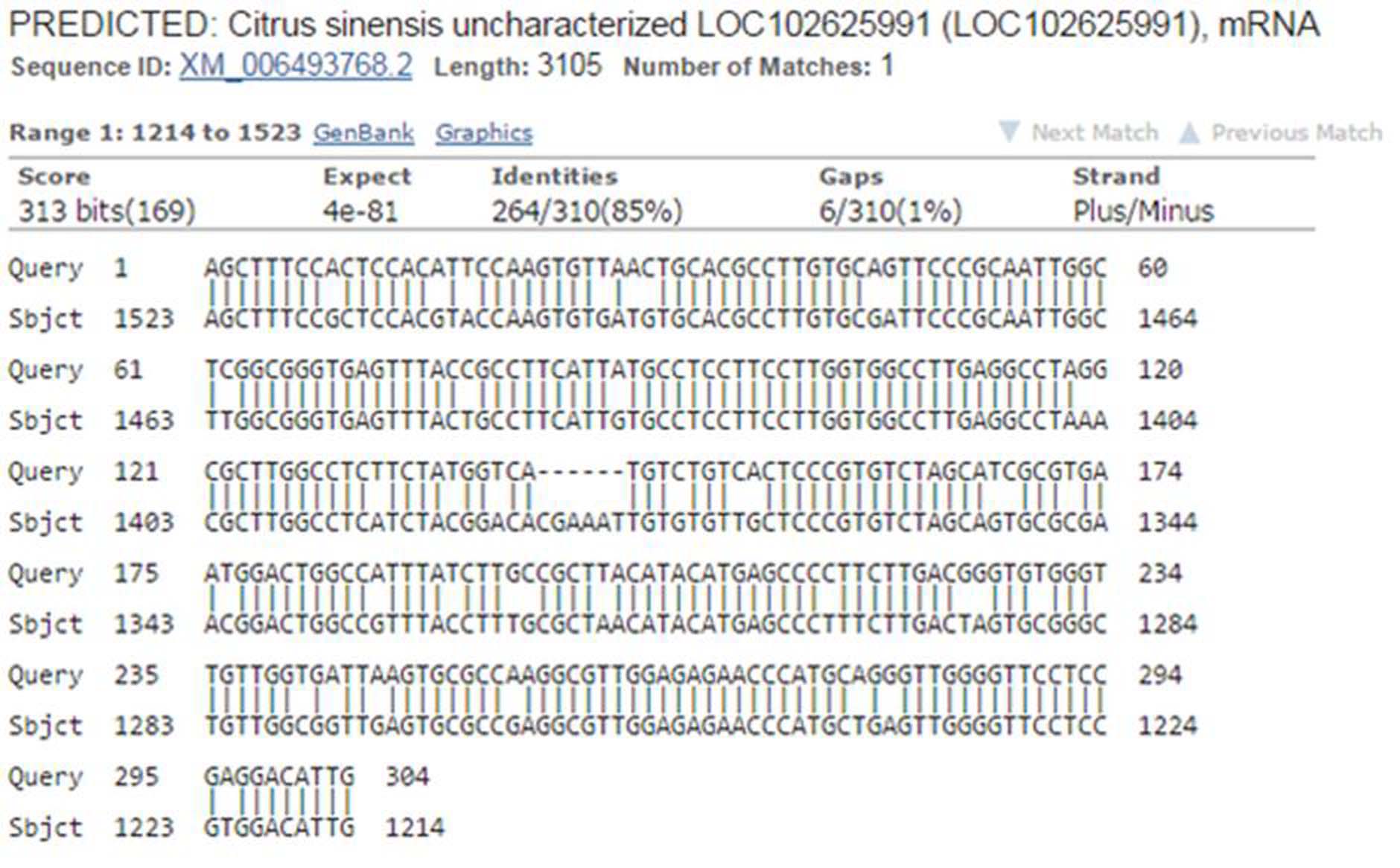


**e *Hind*III 40**


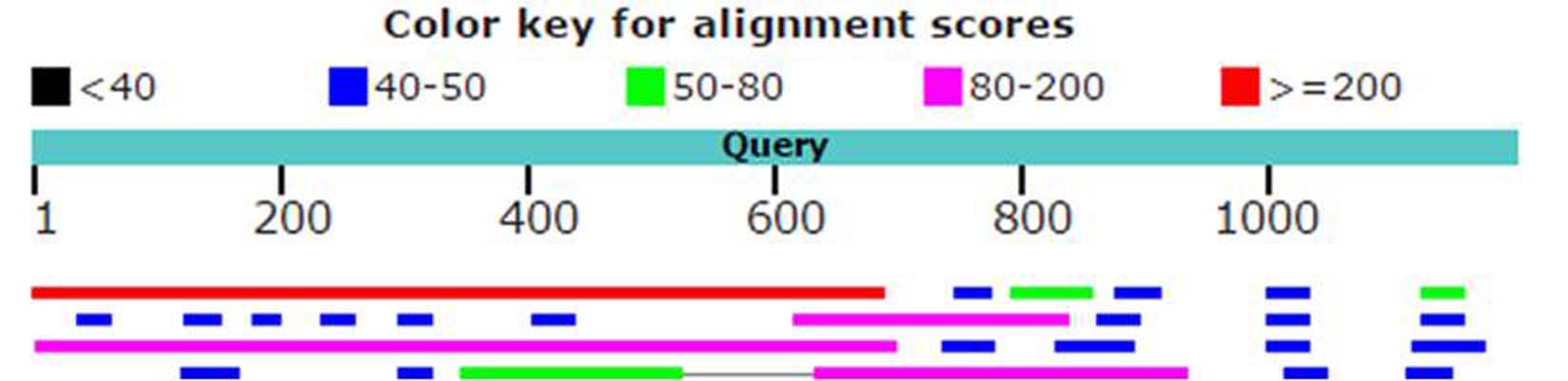


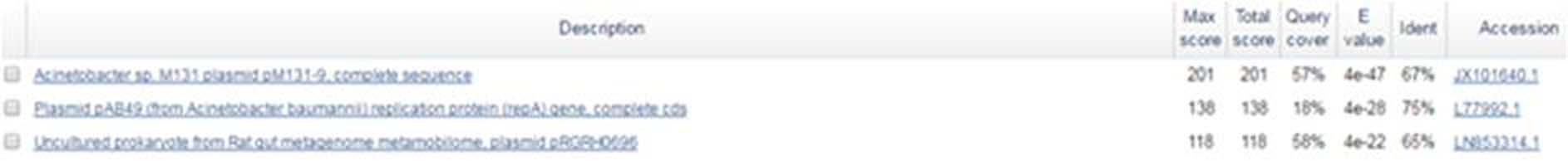


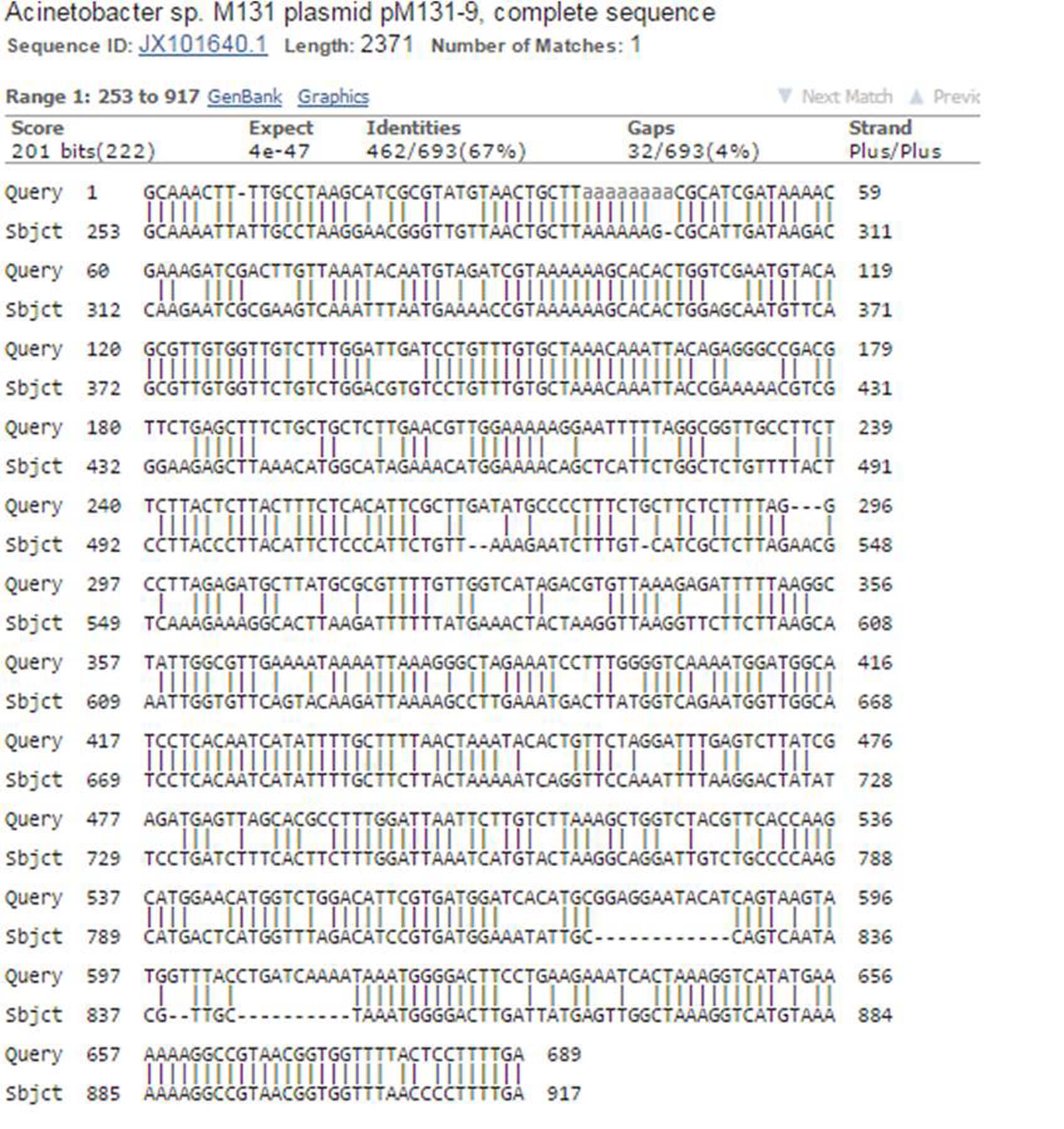


**f *Sac*I 450**


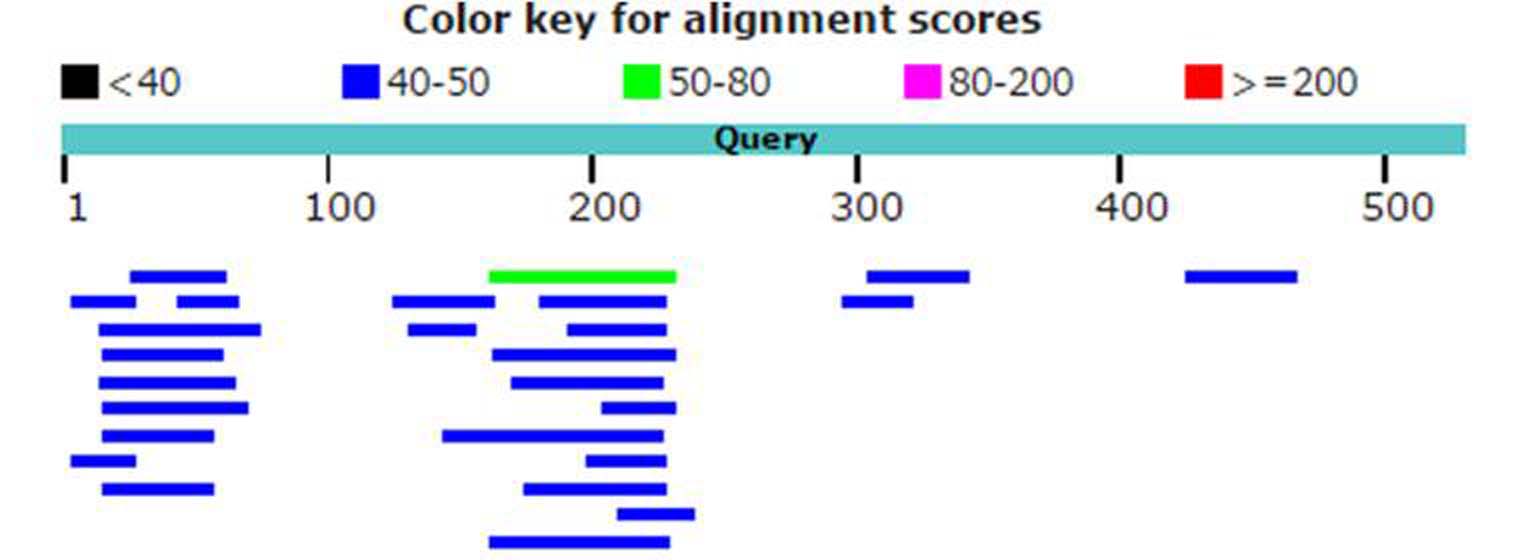


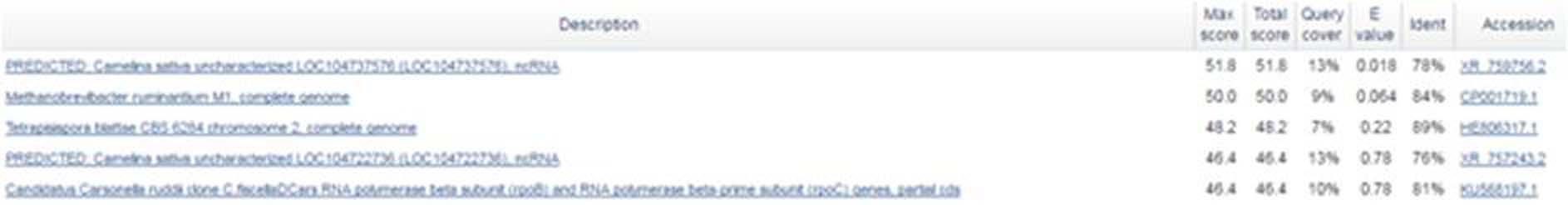


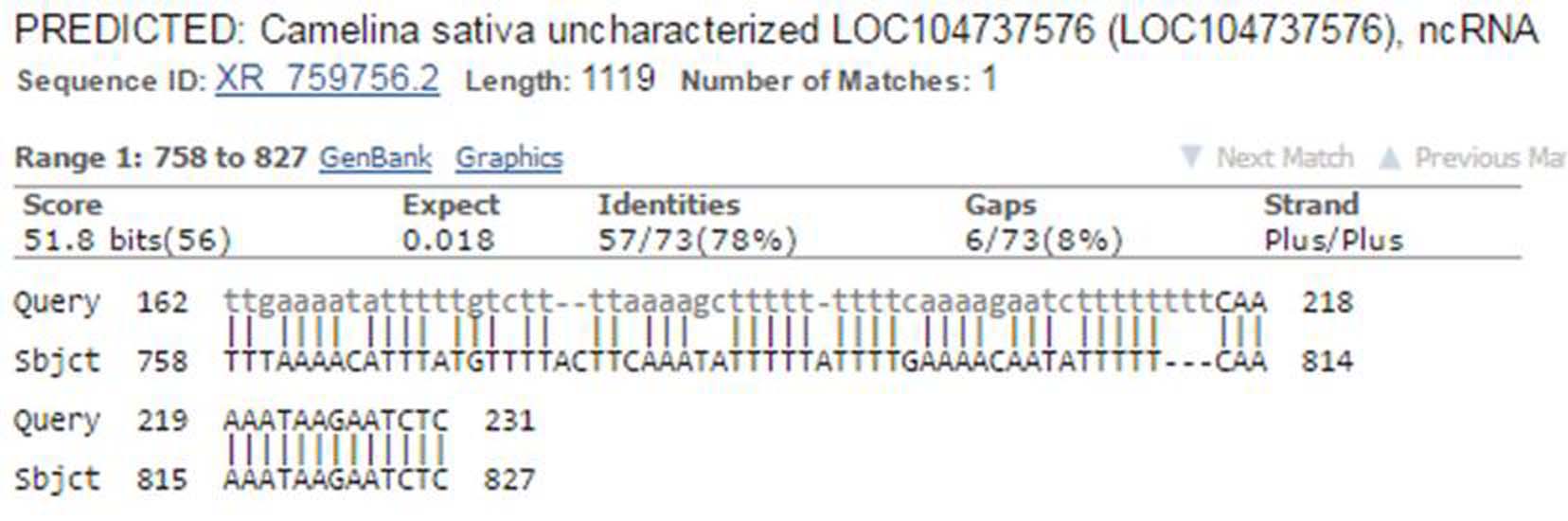


**g *Sac*I 1250**


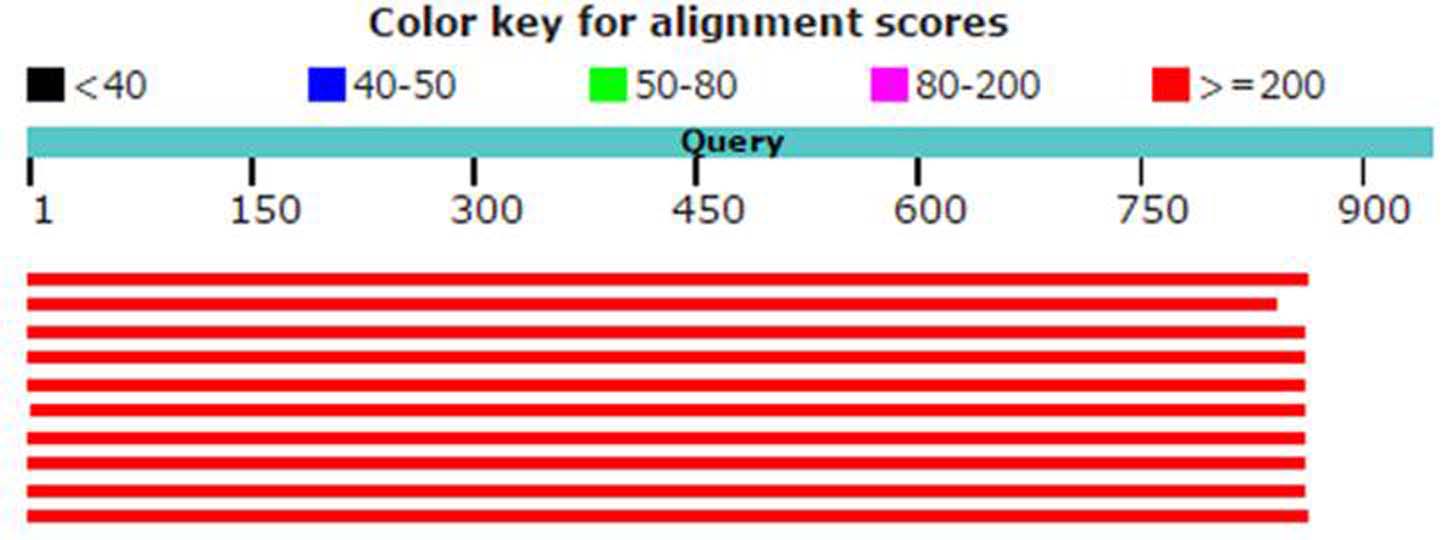


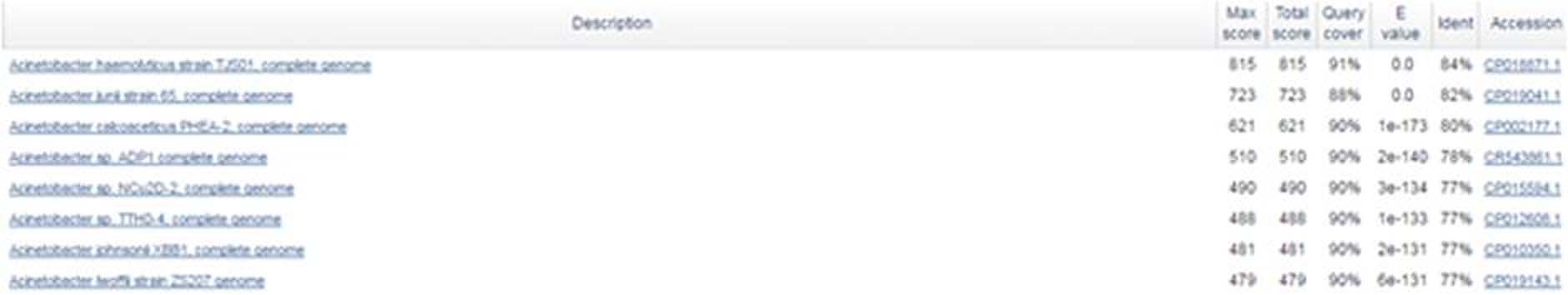


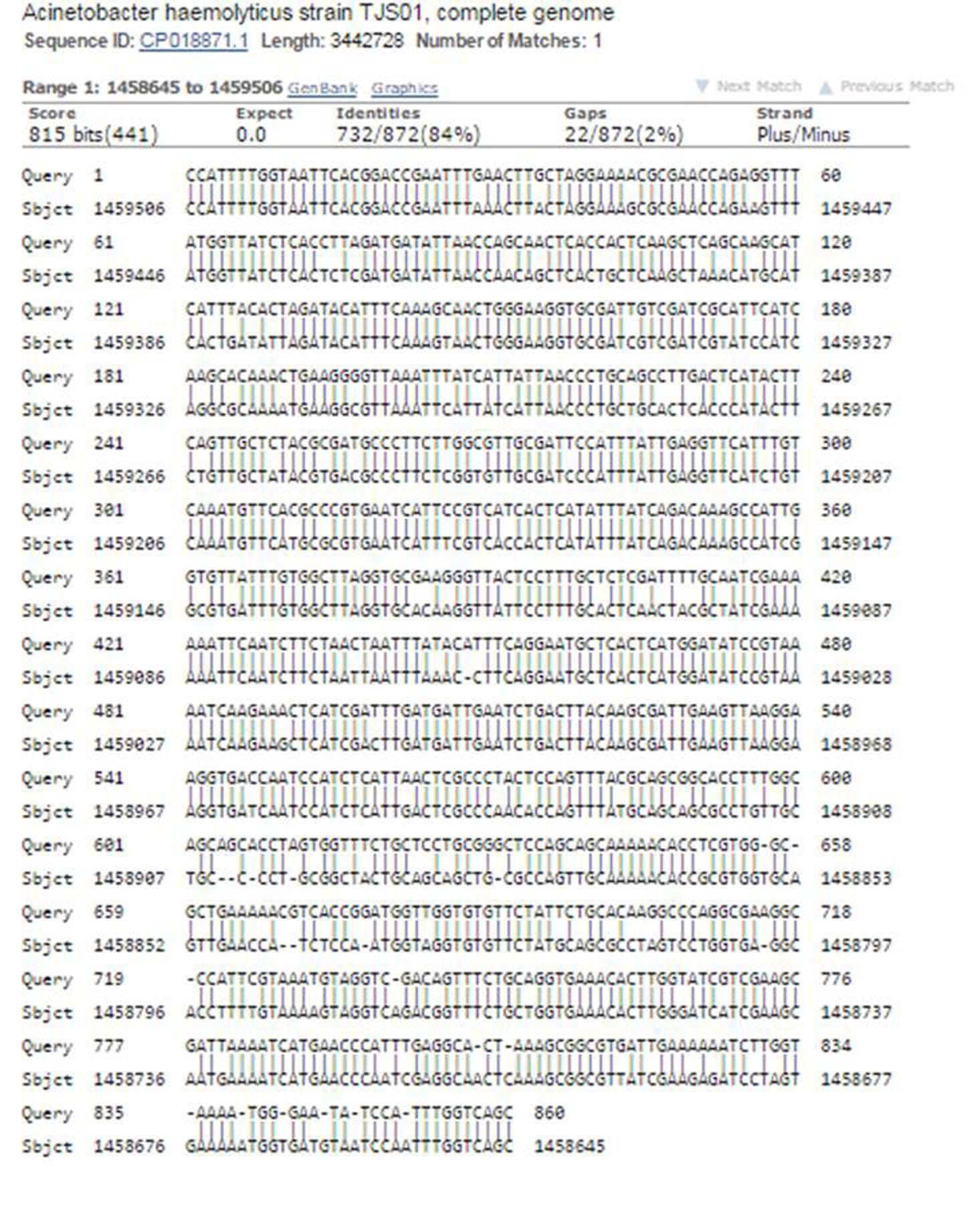


**Figure S6. BLAST Analysis of *N. benthamiana* cloned sequences in NCBI.** *Hind*III and *Sac*I digested RCA products of *N. benthamiana* are cloned and seven clones obtained were sequenced. The sequences were subjected to BLAST analysis in NCBI. The sequence alignment results of the cloned fragments; (**a**) *Sac*I 11, (**b**) *Hind*III 10, (**c**) *Hind*III 33, (**d**) *Hind*III 38, (**e**) *Hind*III 40, (**f**) *Sac*I 450 and (**g**) *Sac*1250 are shown.

**a**

circRNA Niben101Scf27324 – part of the sequence containing the junction point (in yellow), forward primer (in green) and reverse complementary of reverse primer (in blue).

TGAGCCATTCGCAGTTTCACAGTCTGAATTTGTTCATACTTACACATGCATGGCTT.CTTTGAGCACTCTAATTTCTTCAAAGTAACAGCGCCGGAGGCACGACCCGGCCAATTAAGGCCAGGAGCGCATCGCCGGCAGAAGGGACGAGACGACC

Obtained sanger sequence of the divergent PCR product with forward primer

GCATGGCTTAATCTTTGAGACAAGCATATGACTACTGGCAGGATCAACCAGGTAGAGCGTAGGCTT.GCTTTGAGCACTCTAATTTCTTCAAAGTAACAGCGCCGGAGGCACGACCCGGCCAATTAAGGCCAGGAGCGCATCGCCGGCAGAAGGGACGAGACGACC

Green: reverse complementary sequence of reverse primer Red: exact matching sequence Grey: extra base in between black: extra sequence yellow: junction point

Obtained sanger sequence of the divergent PCR product with reverse primer

GCCGGGTCGTGCCTCCGGCGCTGTTACTTTGAAGAAATTAGAGTGCTCAAAGCAAGCCTACGCTCTACCTGGTTGATCCTGCCAGTAGTCATATGCTTGTCTCAAAGATTAAGCCATGCATGTGTAAGTATGAACAAATTCAGACTGTGAAACTGCGAATGGCTCA

Green: reverse complementary sequence of forward primer Red: exact matching sequence

Grey: extra base in between black: extra sequence in between yellow: junction point

After reverse complementing

TGAGCCATTCGCAGTTTCACAGTCTGAATTTGTTCATACTTACACATGCATGGCTTAATCTTTGAGACAAGCATATGACTACTGGCAGGATCAACCAGGTAGAGCGTAGGCTTGCTTTGAGCACTCTAATTTCTTCAAAGTAACAGCGCCGGAGGCACGACCCGGC

Green: forward primer Red: exact matching sequence Grey: extra base in between

black: extra sequence in between yellow: junction point

**b**


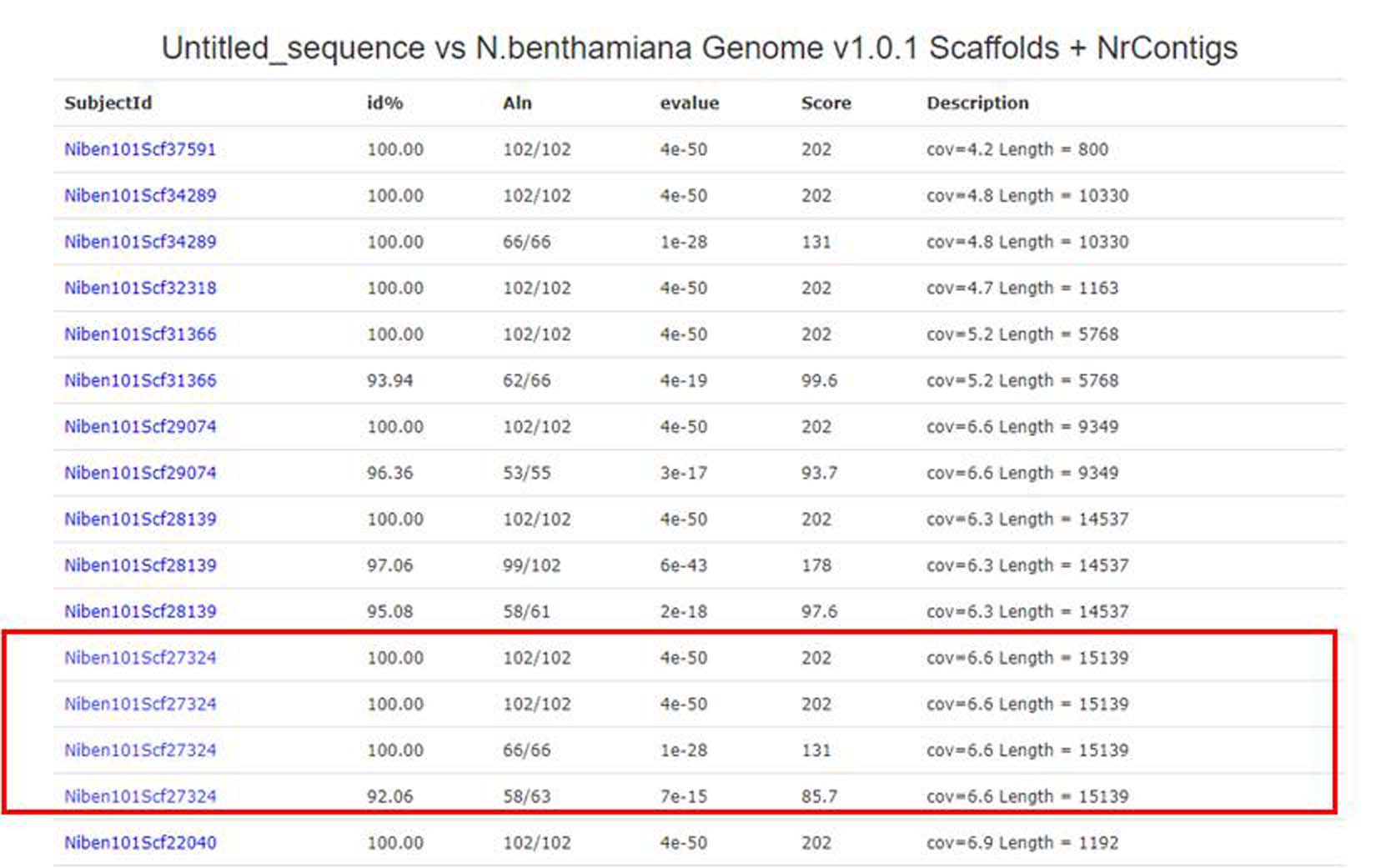


**c**

**
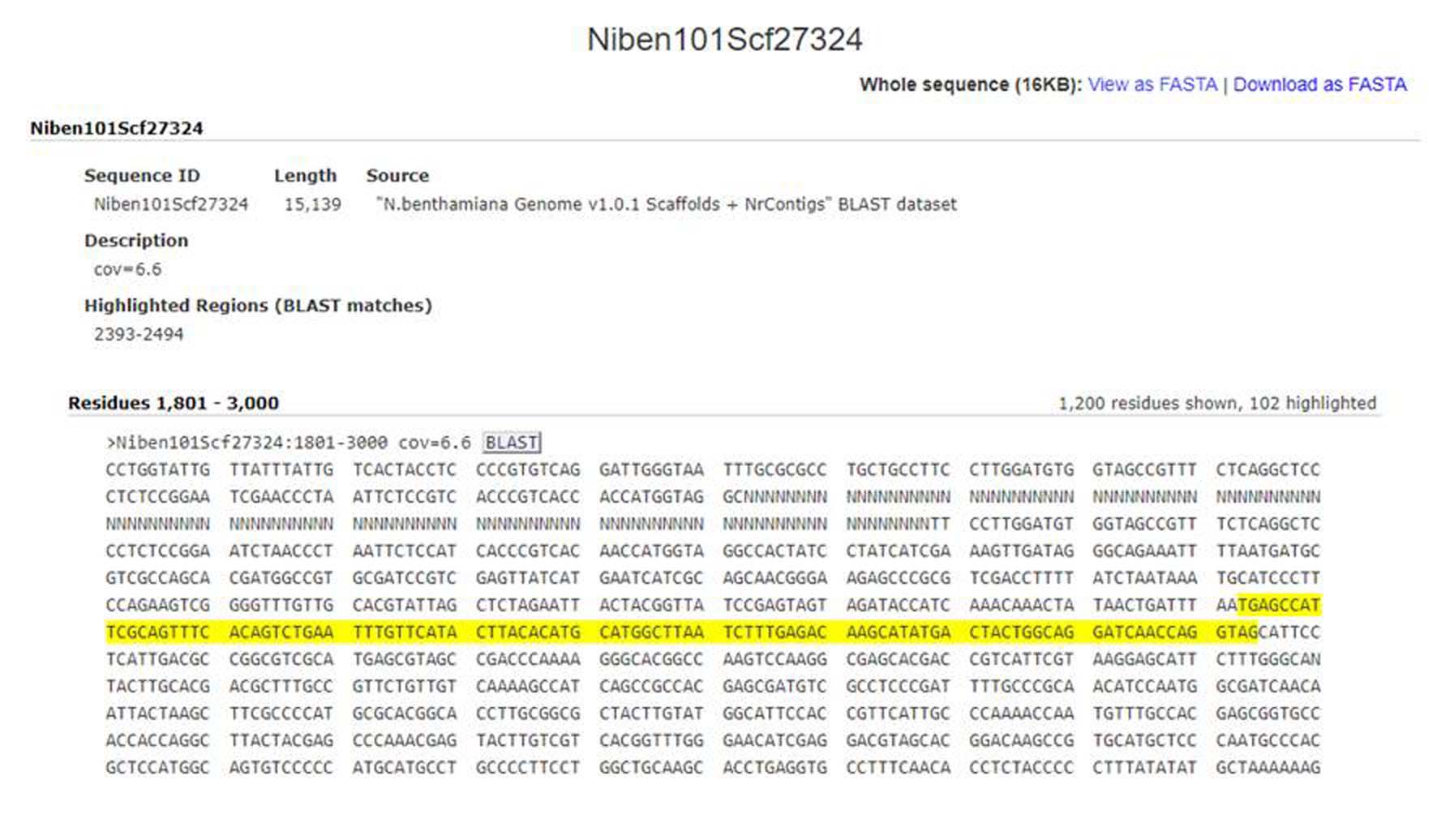
**

**d**

Range 1: 1455 to1556

| **Score** | **Expect** | **Identities** | **Gaps** | **Strand** |
| --- | --- | --- | --- | --- |
| 189 bits(102) | 2e-49 | 102/102(100%) | 0/102(0%) | Plus/Minus |

Query 1 TGAGCCATTCGCAGTTTCACAGTCTGAATTTGTTCATACTTACACATGCATGGCTTAATC 60

||||||||||||||||||||||||||||||||||||||||||||||||||||||||||||

Sbjct 1556 TGAGCCATTCGCAGTTTCACAGTCTGAATTTGTTCATACTTACACATGCATGGCTTAATC 1497

Query 61 TTTGAGACAAGCATATGACTACTGGCAGGATCAACCAGGTAG 102

||||||||||||||||||||||||||||||||||||||||||

Sbjct 1496 TTTGAGACAAGCATATGACTACTGGCAGGATCAACCAGGTAG 1455

Range 2: 2180 to 2245

| **Score** | **Expect** | **Identities** | **Gaps** | **Strand** |
| --- | --- | --- | --- | --- |
| 122 bits(66) | 2e-29 | 66/66(100%) | 0/66(0%) | Plus/Minus |

Query 101 AGAGCGTAGGCTTGCTTTGAGCACTCTAATTTCTTCAAAGTAACAGCGCCGGAGGCACGA 160

||||||||||||||||||||||||||||||||||||||||||||||||||||||||||||

Sbjct 2245 AGAGCGTAGGCTTGCTTTGAGCACTCTAATTTCTTCAAAGTAACAGCGCCGGAGGCACGA 2186

Query 161 CCCGGC 166

||||||

Sbjct 2185 CCCGGC 2180

**e**
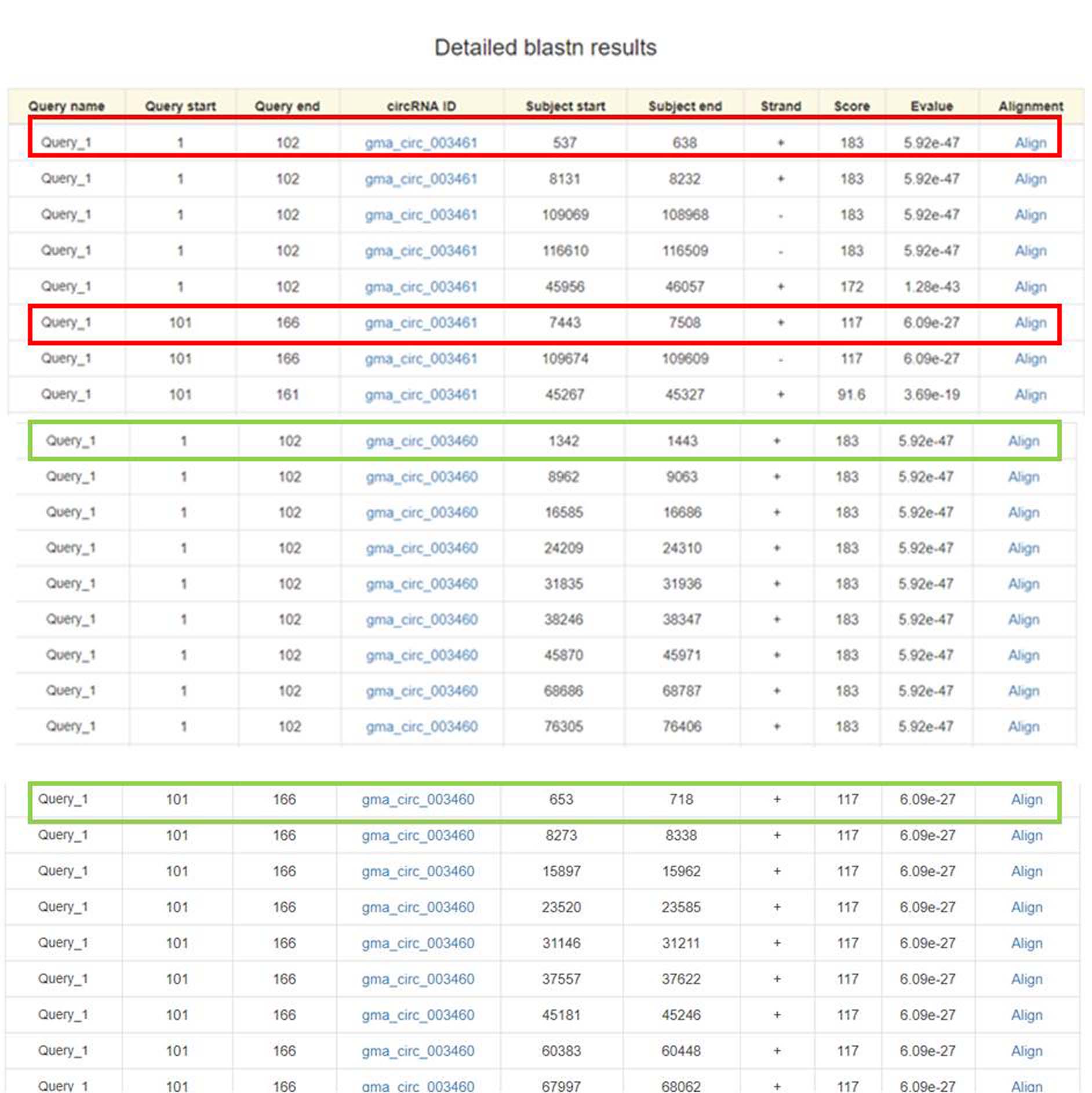


**f**


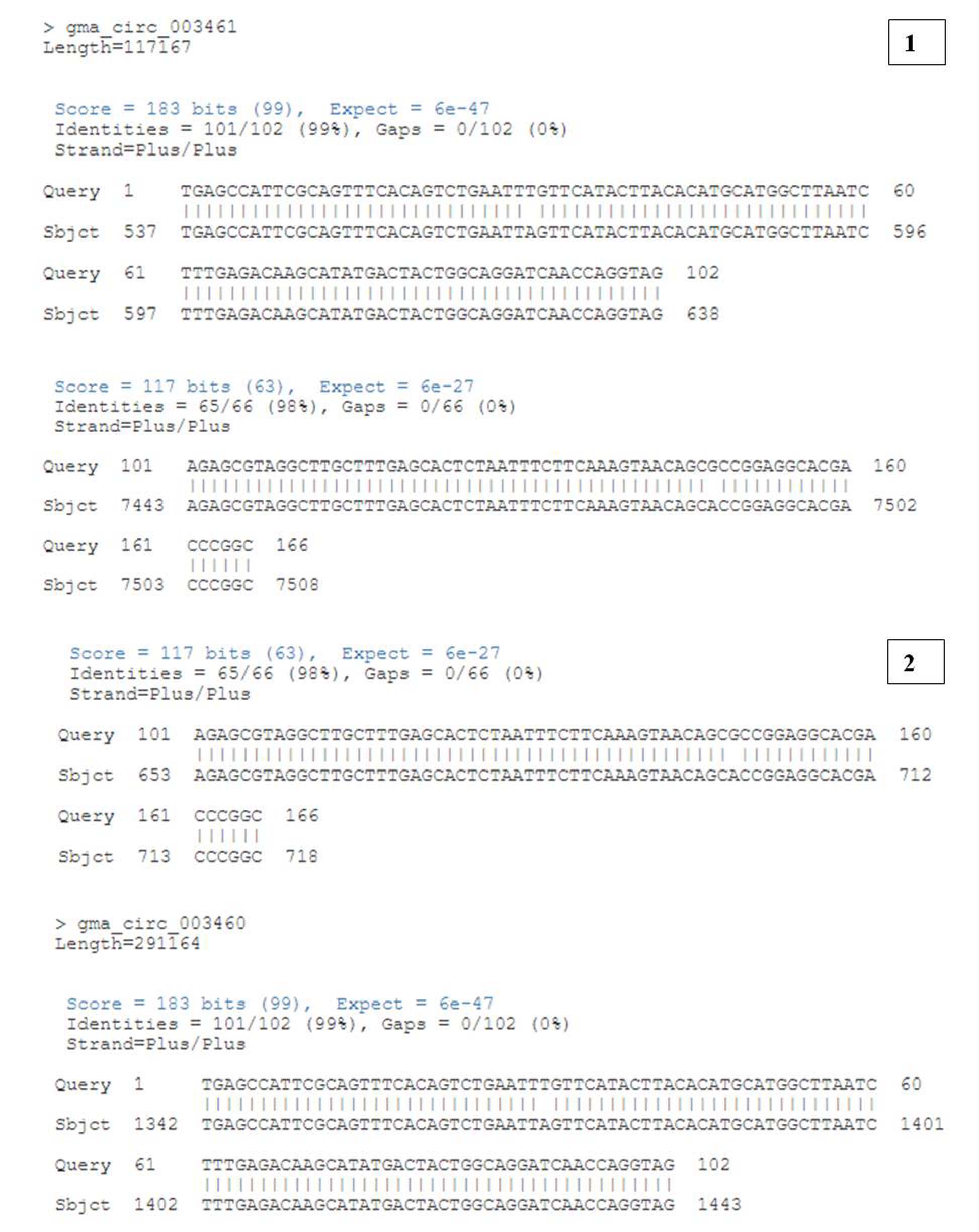


**Figure S7.** **Mapping and validation of *N. benthamiana* circRNA.** **a** CircRNA sequence Niben101Scf27324 obtained by NGS-tdMDA and sanger sequencing. **b** Summary of BLAST (Sol Genomics Network) results; query sequence v/s *N. benthamiana* Genome v1.0.1 Scaffolds + NrContigs. **c** Individual BLAST results showing the matching sequence. **d** BLAST results showing sequences producing significant alignments. **e** Summary of blastn results against *Glycine max* circRNAs, grna-circ_003460 and grna-circ_003461. **f** Blastn results showing significant alignments against grna-circ_003461 (1) and grna-circ_003460 (2).


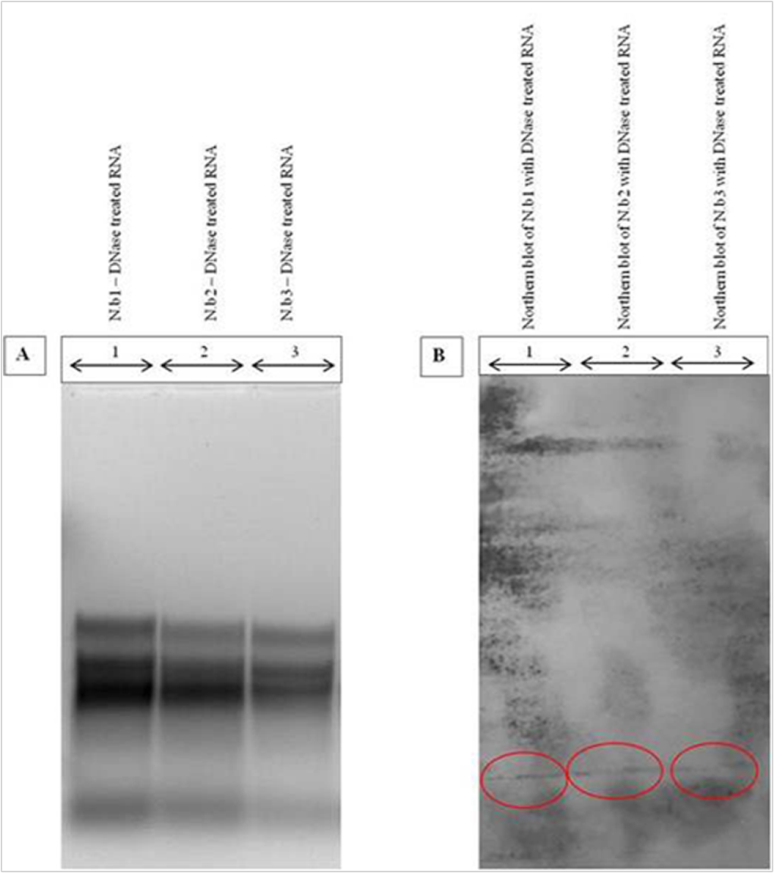


**Figure S8. Northern blotting for confirmation of circRNA.** **a** Total RNA from three *N. benthamiana* plants were run on 1% MOPS-formaldeyde gel. **b** Faint signal was detected from all the samples after hybridised with nb_circ7 primer derived probe specifically designed for circRNA ID, Niben101Scf27324 predicted from NGS-tdMDA.
